# RUNX3 inactivates oncogenic MYC through disruption of MYC/MAX complex and subsequent recruitment of GSK3β-FBXW7 cascade

**DOI:** 10.1038/s42003-023-05037-0

**Published:** 2023-07-03

**Authors:** Vincent Oei, Linda Shyue Huey Chuang, Junichi Matsuo, Supriya Srivastava, Ming Teh, Yoshiaki Ito

**Affiliations:** 1 Cancer Science Institute of Singapore, National University of Singapore, Singapore, Singapore; 2 NUS Graduate School, Integrative Sciences and Engineering Programme, Singapore, Singapore; 3 Department of Medicine, National University of Singapore, Singapore, Singapore; 4 Department of Pathology, National University of Singapore, Singapore, Singapore

**Keywords:** Tumour-suppressor proteins, Cell growth

## Abstract

*MYC* is one of the most commonly dysregulated proto-oncogenes in cancer. *MYC* promotes cancer initiation and maintenance by regulating multiple biological processes, such as proliferation and stem cell function. Here, we show that developmental regulator RUNX3 targets MYC protein for rapid degradation through the glycogen synthase kinase-3 beta-F-box/WD repeat-containing protein 7 (GSK3β-FBXW7) proteolytic pathway. The evolutionarily conserved Runt domain of RUNX3 interacts directly with the basic helix–loop–helix leucine zipper of MYC, resulting in the disruption of MYC/MAX and MYC/MIZ-1 interactions, enhanced GSK3β-mediated phosphorylation of MYC protein at threonine-58 and its subsequent degradation via the ubiquitin-proteasomal pathway. We therefore uncover a previously unknown mode of MYC destabilization by RUNX3 and provide an explanation as to why RUNX3 inhibits early-stage cancer development in gastrointestinal and lung mouse cancer models.

## Abstract (web summary)

A novel tumor suppressing role for RUNX3 is shown where a direct interaction with MYC leads to disruption of MYC-MAX heterodimers and consequent degradation of the MYC oncoprotein.

## Introduction

The proto-oncogene MYC regulates a broad array of transcriptional programs that are critical for stem cell function, somatic cell proliferation and differentiation in normal tissues^1^. The prevalence of deregulated MYC expression in diverse cancer types suggests that heightened MYC activity contributes to the pathogenesis of most, if not all, cancers^2–4^. MYC has been shown to play pivotal roles in cancer initiation and its maintenance^5^. MYC is one of the four transcription factors (also known as Yamanaka factors) that can reprogram somatic cells to pluripotency^6^. Indeed, MYC was linked to the activation of an embryonic stem cell-like program in differentiated adult epithelial cells and thus, implicated in tumor initiation and the self-renewal capability of cancer stem cells^7^. Mechanistic links between MYC and oncogenic signaling pathways are pivotal to its oncogenic drive^8^. MYC was the earliest identified cooperating partner of RAS during tumorigenic conversion^9^. Moreover, *MYC* was identified as a target of the APC pathway – its promoter is strongly induced by Wnt effectors TCF4/β-catenin^10^. *Myc* deletion rescued *Apc* deletion in the adult mouse small intestine, thereby highlighting the importance of Myc for the activation of oncogenic Wnt signaling during early neoplasia^11^.

The role of MYC in cancer maintenance is best exemplified by the fact that inhibition of MYC led to rapid tumor regression in multiple solid tumor types^1,4^. MYC promotes cancer cell proliferation through its ability to regulate genes involved in fundamental growth processes such as cell cycle progression, ribosomal biogenesis, and metabolism^12^. More recently, persistent MYC activity was reported to drive cell extrinsic changes in host immunity and tumor microenvironment for the maintenance of pancreatic adenocarcinoma phenotype^13^. MYC addiction is therefore a cancer vulnerability that can be therapeutically exploited through targeting its activity^14^.

Perhaps the most effective and tumor-specific mode of MYC inhibition is to directly target the transcriptional properties of the MYC protein^14^. The MYC protein N-terminus region harbors a transcription regulatory domain, which effects transcriptional activation and repression. The C-terminus of MYC contains basic helix–loop–helix leucine zipper (bHLH-LZ) domain, which heterodimerizes with MYC-associated protein X (also known as MAX) to bind the DNA sequence 5′-CACGTG-3′ (also known as the E-box)^4,15–17^. MYC/MAX heterodimerization is necessary for MYC-driven tumorigenesis^18,19^. Disruption of the MYC/MAX complex using Omomyc, a MYC mutant peptide that interferes with the MYC dimerization domain, resulted in rapid regression of *Ras*-induced lung adenocarcinoma^20^. Interestingly, Omomyc treatment also led to a reduction in MYC protein levels through ubiquitination and degradation^21^. The development of small molecule inhibitors that disrupt MYC/MAX heterodimerization further emphasized the importance of promoting MYC degradation to suppress tumor growth and enhance immunotherapy^22,23^.

The RUNX family of developmental transcription factors, comprising RUNX1, 2 and 3, has been strongly implicated in tumorigenesis^24,25^. The prototype RUNX protein is characterized by the evolutionarily conserved Runt domain, which enables heterodimerization with obligate partner CBFβ and subsequent stable binding to DNA^26–28^. The *RUNX3* gene is frequently hypermethylated and epigenetically silenced in diverse solid tumors, suggesting that it plays pivotal roles in tumor suppression^24^. We found that the adenoma-prone intestines of *Runx3* deficient mice exhibited elevated *Myc* expression and attributed it to the ability of RUNX3 to attenuate oncogenic Wnt signaling^29^. Moreover, *Runx3* inactivation was crucial for the progression of adenoma to adenocarcinoma during *K-ras*-induced lung adenocarcinoma development^30^. We had earlier identified the point mutation R122C in *RUNX3* in human gastric cancer^31^. More recently, we reported that the *RUNX3*^*R122C*^ knock-in mice exhibited a precancerous phenotype, characterized by a massive increase in rapidly proliferating isthmus stem/progenitor cells and reduced differentiated cell populations, in the stomach corpus tissue^32^. Transcriptomic analyses revealed the strong enrichment of the *MYC* target gene signature in the corpus epithelial cells of *RUNX3*^*R122C/R122C*^ mice compared with its wild-type counterpart^32^. Interestingly, RUNX3 was found to promote the degradation of closely related MYC paralog MYCN in neuroblastoma^33^.

In this study, we continued our investigations into the molecular mechanisms underlying the RUNX3-MYC relationship. We examined whether RUNX3 serves as a safeguard against MYC-driven proliferation during malignant progression. The prevalence of *RUNX3* hypermethylation in cancer and the potential for pharmacological reversion suggest that reactivating the silenced *RUNX3* may be a first-line treatment for MYC addiction in such cancers.

## Results

### RUNX3 inhibits the MYC pathway

**Fig. 1:**
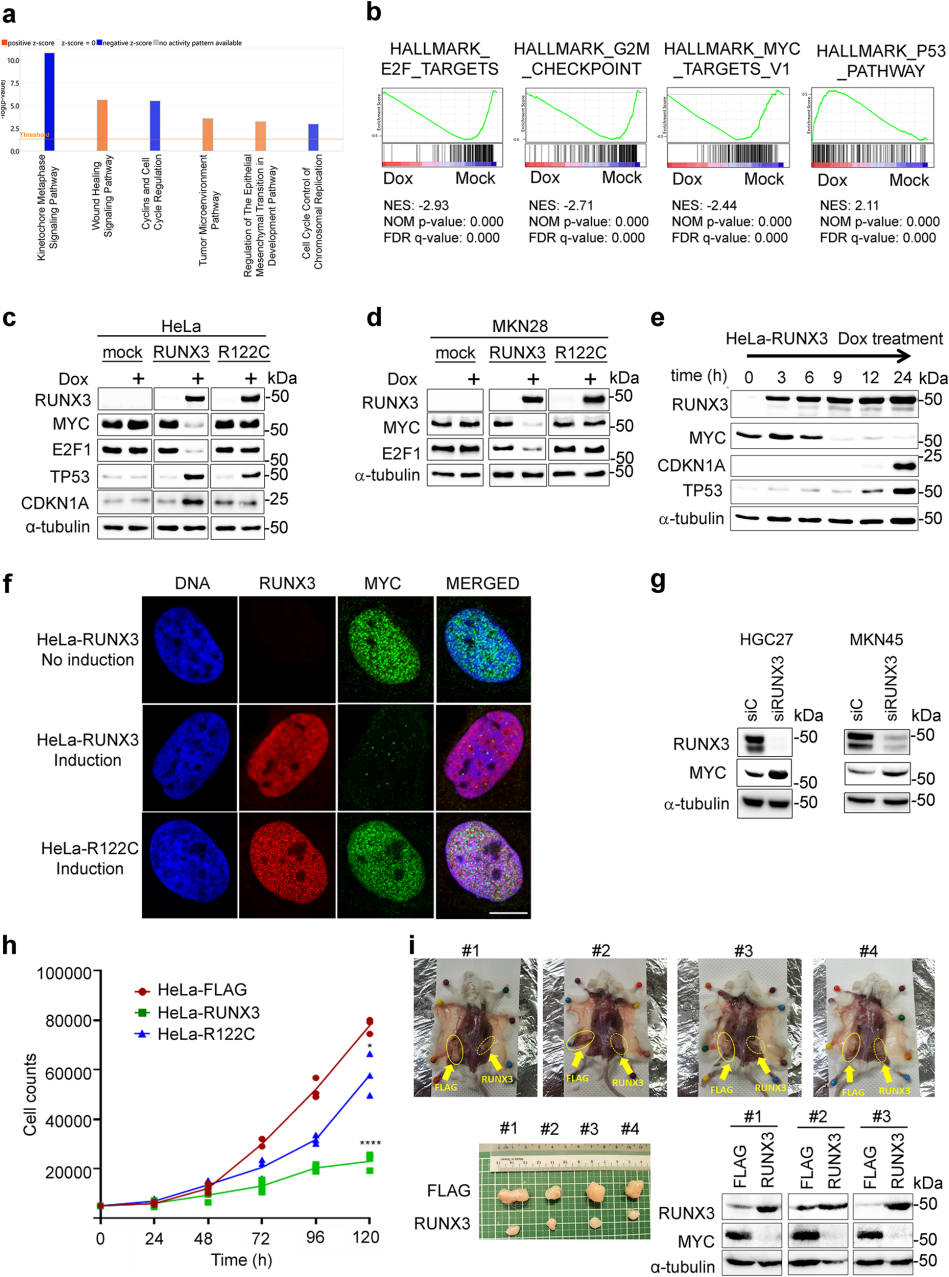
RUNX3 inhibits the MYC tumorigenic pathway. **a** Canonical pathway analysis (using Ingenuity Pathway Analysis by QIAGEN) of RNA-sequencing (RNAseq) data of HeLa-RUNX3 treated with 500 ng/ml Dox for 48 h or untreated (mock) in triplicates. Differentially expressed genes (DEGs) were filtered using adjusted p value of <0.01 and log_2_ fold change of ±0.4. Orange and blue bars represent activated and inactivated pathways respectively. **b** Gene set enrichment analysis (GSEA) of RNAseq from (**a**). **c** Immunoblot of lysates (40 µg) from HeLa-FLAG, HeLa-RUNX3, HeLa-R122C treated with 500 ng/ml doxycycline for 48 h. RUNX3 was detected by anti-FLAG antibody. α-tubulin is the loading control. The data is representative of three independent experiments. **d** Immunoblot of lysates (40 µg) from MKN28-FLAG, MKN28-RUNX3, MKN28-R122C treated with 10 ng/ml doxycycline for 48 h. The data is representative of three independent experiments. **e** Immunoblot of HeLa-RUNX3 treated with 500 ng/ml Dox for the indicated timepoints. The data is representative of two independent experiments. **f** Representative immunofluorescence staining of cells treated with 500 ng/ml Dox for 24 h. Anti-FLAG and anti-MYC antibodies were used to detect RUNX3 (red) and MYC (green) respectively. DNA was counterstained by DAPI. Scale bar, 10 µm. The data is representative of three independent experiments. **g** Immunoblot of lysates (40 µg) from HGC27 and MKN45 cells treated with control siRNA (siC) and siRNA targeting RUNX3 (siRUNX3). The data is representative of four independent experiments. **h** Proliferation assay of HeLa-FLAG, HeLa-RUNX3, HeLa-R122C treated with 500 ng/ml Dox. Cells were manually counted every 24 h up to 120 h (*n* = 3). Data is presented as mean ± standard deviation. Asterisks indicate significant differences between groups at 120 h time point, *p ≤ 0.05, ****p ≤ 0.0001. Data is representative of three independent experiments. **i** MKN28-FLAG and MKN28-RUNX3 cells were injected into NOD scid gamma (NSG) mice at the left and right flanks respectively. Mice were fed with Dox-embedded food pellets. Tumor xenografts – when one graft site reached 1.5 cm diameter – were harvested for immunoblot (70 µg of lysate). Experiment is performed once with 4 mice.

To explore the major roles of RUNX3 in cancer, we generated stably transduced doxycycline (Dox)-inducible Tet-On expression of RUNX3 in the cervical cancer cell line HeLa (henceforth designated as HeLa-RUNX3) and performed transcriptomic profiling of HeLa-RUNX3 in the presence or absence of doxycycline. RNA sequencing (RNAseq) followed by Ingenuity Pathway Analysis (IPA) indicated downregulation of proliferation-related processes and upregulation of wound healing and metastasis signaling (Fig. 1a). Our findings agree with the seminal work that RUNX3 inhibited proliferation, while promoting a metastatic program in pancreatic cancer^34^. In support of its anti-proliferation function, upstream regulators predicted to be activated (indicated as orange) by RUNX3 included *TP53, CDKN1A, CDKN2A, RB*, and *SMAD3/4*, while those predicted to be repressed by RUNX3 included proliferation-associated *E2F1, MYC, CCND1, FOXM1*, and estrogen receptor α (Supplementary Fig. 1a, left panel). Moreover, RUNX3 activity was predicted to repress multiple tumor types (Supplementary Fig. 1b, left). Gene set enrichment analysis (GSEA) revealed strong downregulation of proliferation-associated programs such as HALLMARK_E2F_TARGETS, HALLMARK_G2M_CHECKPOINT, and HALLMARK_MYC_TARGETS_V1 following the induction of RUNX3 with Dox (Fig. 1b). These findings agreed with our earlier flow cytometry analysis showing that RUNX3 induction in HeLa-RUNX3 cells strongly inhibited G1-S phase transition^35^. Conversely, RUNX3 induction was associated with upregulation of the p53 pathway in HeLa cells (Fig. 1b), in line with earlier findings that RUNX3 cooperated with p53 during DNA damage^36^.

Western blot analysis of the HeLa-RUNX3 cells revealed dramatic reductions of MYC and E2F1 proteins after Dox-induction of RUNX3 (Fig. 1c). This effect was not observed when RUNX3 mutant R122C was induced. RUNX3 induction was also associated with increases in p53 and cell cycle/CDK inhibitor CDKN1A (also known as p21^WAF/CIP1^) protein levels (Fig. 1c). While R122C induction was also associated with an increase in the p53 protein level, CDKN1A protein level was unaffected (Fig. 1c). To assess the influence of p53 on RUNX3-mediated changes in proliferation-associated proteins, we generated the stably transduced Dox-inducible Tet-On expression of RUNX3 in the p53-mutated gastric cancer MKN28 cell line (henceforth, MKN28-RUNX3). RNAseq of MKN28-RUNX3, followed by IPA and GSEA, indicated similar pathway enrichment profiles for RUNX3 induction, when compared to HeLa-RUNX3 (Supplementary Fig. 1a–c). Dox-induction of RUNX3 in MKN28-RUNX3 resulted in strong reduction of MYC and E2F1 proteins, unlike the R122C mutant (Fig. 1d). Since siRNA-mediated knockdown of MYC and E2F1 resulted in strong and moderate growth inhibition in HeLa-RUNX3, respectively (Supplementary Fig. 1d, bottom panel), we chose the RUNX3-MYC relationship for further studies. Of note, MYC mRNA level in HeLa-RUNX3 was not significantly altered following RUNX3 induction (Supplementary Fig. 1e).

Time course Dox treatment in HeLa-RUNX3 revealed that RUNX3 induction is followed by rapidly decreasing MYC protein levels, with minimal MYC protein amount by 9 h after Dox addition (Fig. 1e). The induction of p53 and CDKN1A proteins were observed at 12 h and 24 h after Dox addition. Immunofluorescence staining showed that RUNX3 overexpression corresponded with strong depletion of nuclear MYC proteins (Fig. 1f). By contrast, induction of the RUNX3 mutant R122C did not affect nuclear MYC protein levels (Fig. 1f). Overexpression of RUNX3 in an array of gastric cancer cell lines resulted in the reduction of MYC proteins in all tested cell lines (Supplementary Fig. 1f). Accordingly, siRNA-mediated depletion of endogenous RUNX3 in HGC27 and MKN45 cell lines resulted in upregulation of the MYC protein (Fig. 1g). Moreover, knockdown of RUNX3 resulted in negligible decrease in MYC mRNA and strong growth stimulation in HGC27 cells (Supplementary Fig. 2a, b). Together, the results suggest that RUNX3 targets MYC protein directly to suppress proliferation. Indeed, cell proliferation, NOD SCID gamma (NSG) mice tumorigenesis and tumoroid formation assays showed that overexpression of RUNX3, accompanied by reduction of MYC, is strongly associated with inhibition of proliferation and tumorigenic growth in HeLa and MKN28 cell lines (Fig. 1h, i and Supplementary Fig. 2c–i). The RUNX3-R122C mutant, which was not associated with reduced MYC protein levels, was impaired in anti-tumorigenic activities, when compared to wild-type RUNX3 (Fig. 1h and Supplementary Fig. 2c–f, i).

### RUNX3 promotes MYC degradation via the GSK3β-FBXW7 pathway

**Fig. 2:**
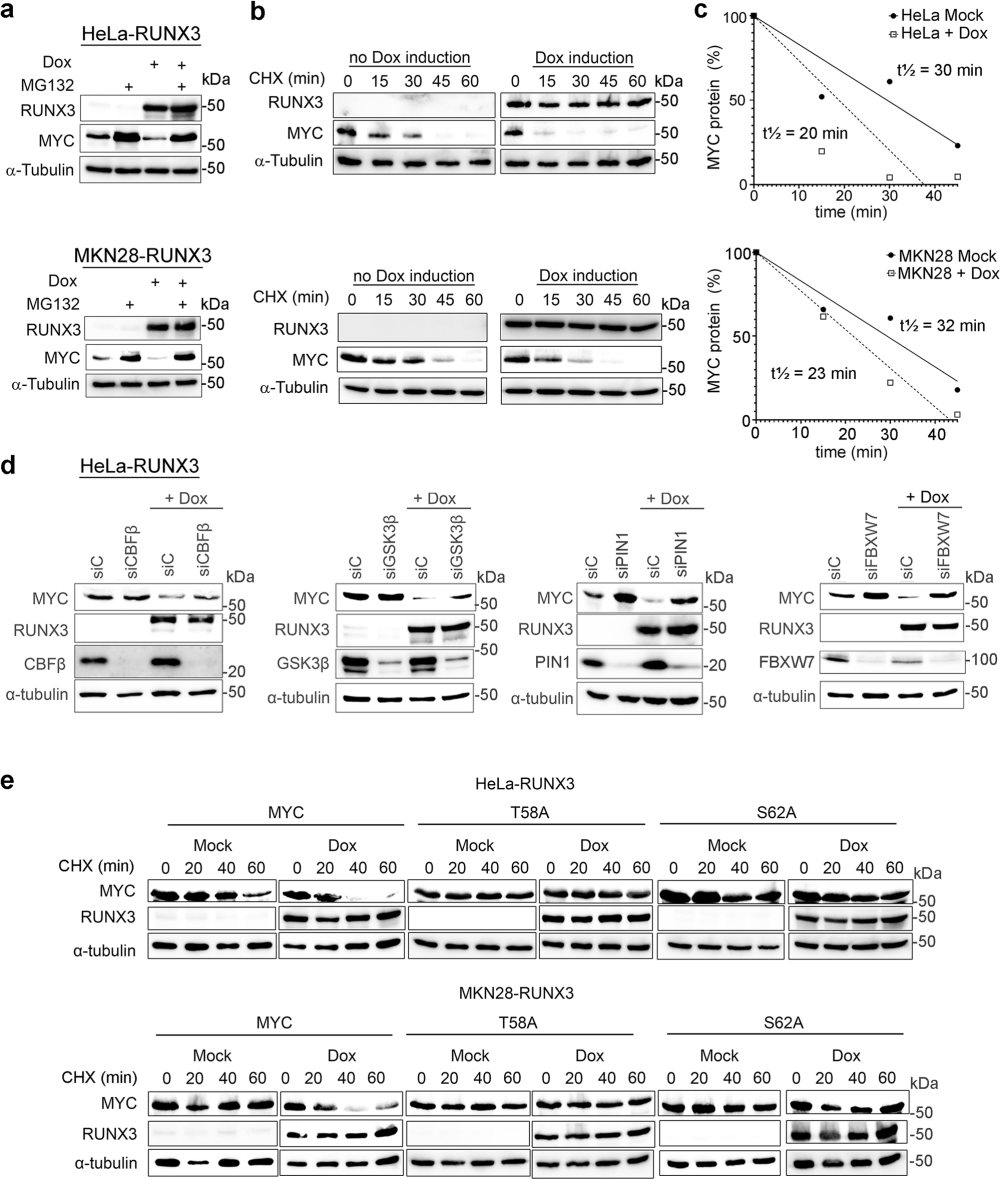
RUNX3 promotes MYC protein degradation via the GSK3β-FBXW7 pathway. **a** HeLa-RUNX3 and MKN28-RUNX3 were treated with 500 ng/ml and 20 ng/ml Dox, respectively. 18 h after Dox addition, 10 µM MG132 was added where indicated for 6 h. Immunoblot was performed on 40 µg lysate. RUNX3 was detected by anti-FLAG antibody. α-tubulin is the loading control. The data is representative of 3 independent experiments. **b** HeLa-RUNX3 and MKN28-RUNX3 were treated with 500 ng/ml and 20 ng/ml Dox, respectively. 12 h after Dox addition, cycloheximide (CHX) was added at 50 µg/ml and 100 µg/ml for HeLa-RUNX3 and for MKN28-RUNX3, respectively. Immunoblot was performed on 40 µg lysate. The data is representative of three independent experiments. **c** Densitometric quantification of band intensity from **b** using ImageJ software. Lines are fitted by linear regression using GraphPad Prism. **d** HeLa-RUNX3 was transfected with control siRNA (siC), and siRNA targeting CBFβ (siCBFβ), GSK3β (siGSK3β), PIN1 (siPIN1), and FBXW7 (siFBXW7). After 12 h, 500 ng/ml Dox was added where indicated. Cells were harvested 48 h after Dox addition for immunoblot. The data is representative of three independent experiments. **e** Cells were transfected with 2× HA-tagged MYC, 2× HA-tagged-T58A MYC, and 2x HA-tagged-S62A-MYC for 24 h. Immediately following transfection, Dox was added. Dox and CHX treatments were as described in **b**. The data is representative of two independent experiments.

RNAseq of HeLa-RUNX3 revealed negligible change in MYC mRNA abundance, suggesting that RUNX3 directly affects MYC protein stability. RUNX3-associated reduction of MYC protein level was abolished by the proteasome inhibitor Z-Leu-Leu-Leu-al (MG132; Fig. 2a), indicating that RUNX3 promotes ubiquitin-mediated proteasomal degradation of MYC. To assess the half-life of MYC in the presence of RUNX3, we performed cycloheximide chase experiments on HeLa-RUNX3 and MKN28-RUNX3 in the presence and absence of Dox (Fig. 2b). Dox-induction of RUNX3 in HeLa-RUNX3 and MKN28-RUNX3 reduced the half-life of MYC from 30 to 20 min and 32 to 23 min respectively (Fig. 2b, c).

The main mechanism of MYC proteasomal degradation is well characterized – extracellular signal-regulated kinases (ERKs) and cyclin-dependent kinases (CDKs) phosphorylate MYC at Serine 62 (S62); S62 phosphorylation leads to glycogen synthase kinase-3 beta (GSK3β) mediated phosphorylation of Threonine 58 (T58); prolyl isomerase PIN1 facilitates protein phosphatase 2A (PP2A) in the dephosphorylation of phospho-S62; phosphorylation of MYC at T58 results in MYC ubiquitination by E3 ligase complex SKP1-CUL1- F-box/WD repeat-containing protein 7 (SCF^Fbxw7^) and subsequent degradation by 26S proteasome^4,37^. To understand the relationship of RUNX3 with the components of the MYC degradation pathway, we performed siRNA-mediated knockdown of GSK3β, FBXW7 (a component of E3 ubiquitin ligase SCF^Fbxw7^), PIN1 as well as the obligate partner of RUNX3 CBFβ in HeLa-RUNX3 and MKN28-RUNX3 (Fig. 2d and Supplementary Fig. 3a). Surprisingly, depletion of CBFβ partially diminished RUNX3-mediated MYC degradation (Fig. 2d and Supplementary Fig. 3a). CBFβ may therefore enhance the action of RUNX3 on MYC. Knockdown of GSK3β, FBXW7 and PIN1 strongly reduced the ability of RUNX3 to mediate MYC degradation, indicating that RUNX3 functions in the main MYC degradation pathway, possibly by influencing MYC phosphorylation (Fig. 2d and Supplementary Fig. 3a).

We next generated MYC phosphorylation mutants, namely the non-phosphorylatable T58A and S62A, by site directed mutagenesis. The mutants were overexpressed in the HeLa-RUNX3 and MKN28-RUNX3 cell lines and assessed for their stability following Dox treatment in cycloheximide chase experiments (Fig. 2e). While the wild-type MYC protein was rapidly depleted upon Dox-induction of RUNX3, the levels of both T58A and S62A MYC mutant proteins were unchanged (Fig. 2e and Supplementary Fig. 3b). This observation supports the notion that RUNX3 promotes GSK3β-FBXW7-mediated degradation of MYC by modulating MYC phosphorylation.

### RUNX3 directly interacts with MYC through the evolutionarily conserved Runt domain

**Fig. 3:**
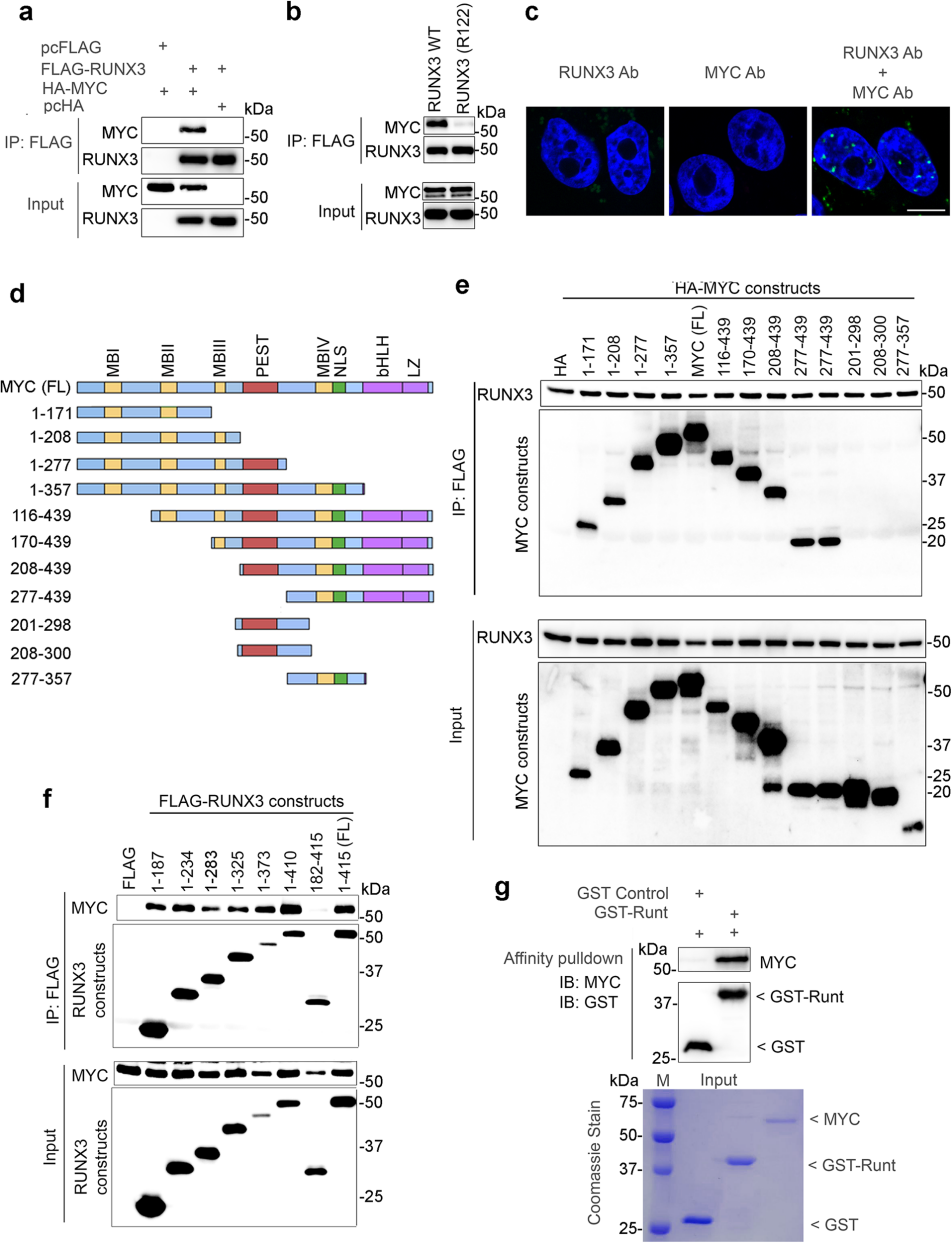
RUNX3 directly interacts with MYC through the evolutionarily conserved Runt domain. **a**, **b** Immunoblot of HEK293T cells transfected with the indicated plasmids. pcFLAG and pcHA denote pcDNA-FLAG and pcDNA-HA empty vector controls, respectively. Input is 5% of amount used in IP. The data is representative of four independent experiments. **c** Proximity ligation assay (PLA) of HGC27 cells using anti-MYC and anti-RUNX3 antibodies. Green signals represent MYC and RUNX3 proteins in close proximity. DAPI (blue) indicates nucleus. Scale bar, 10 µm. The data is representative of three independent experiments. **d** Schematic diagram of the structural motifs of MYC protein and the MYC truncation constructs used. MB MYC boxes, PEST peptide sequence enriched with E, P, S, and T amino acids, NLS nuclear localization signal, bHLH basic helix loop helix, LZ leucine zipper. **e** IP of HEK293T cells transfected with FLAG-tagged RUNX3 and HA-tagged MYC constructs. Anti-FLAG and anti-HA antibodies were used for the immunoblot. FL denotes full-length. The data is representative of 3 independent experiments. **f** IP of HEK293T cells transfected with MYC and FLAG-tagged RUNX3 truncations. The data is representative of three independent experiments. **g** GST pulldown using purified recombinant proteins GST-tagged RUNX3 aa 49–187 (denoted as GST-Runt) and MYC. GST is the negative control. The input proteins are shown by Coomassie blue staining. The data is representative of two independent experiments.

To understand how RUNX3 promotes MYC degradation, we next investigated the interaction between RUNX3 and MYC. FLAG-tagged RUNX3 and HA-tagged MYC were exogenously expressed in HEK293T cells. FLAG-tagged RUNX3 and bound proteins were immunoprecipitated using FLAG affinity beads. Western blot analysis revealed strong co-purification of MYC with RUNX3 (Fig. 3a). Interestingly, the RUNX3 R122C mutant did not interact with MYC (Fig. 3b), in line with its inability to promote MYC degradation (Fig. 1c, d). Importantly, MYC co-precipitated with RUNX3 from HGC27 cell lysates at endogenous protein levels (Supplementary Fig. 4a). Proximity ligation assay using MYC- and RUNX3-specific antibodies revealed focal formation, which indicated that endogenous interaction of both proteins mainly occurred in the nucleus of the HGC27 cell line (Fig. 3c). To map the domains of MYC that interact with RUNX3, a series of MYC truncation constructs (Fig. 3d) were co-expressed with full-length RUNX3. Immunoprecipitation showed that MYC bound to RUNX3 at two regions – the N-terminal amino acids (aa) 1–171, encompassing the transcription regulatory domain, and aa 357–439, which contains the bHLH-LZ domain (Fig. 3d, e). Of note, ARF, a downstream transcriptional target of MYC, RUNX1 and 3^30,38,39^, also bound MYC protein via the transcription regulatory and bHLH-LZ domains^40^. Next, immunoprecipitation using RUNX3 truncation constructs with full-length MYC showed that RUNX3 N-terminus domain aa 1–187, which contains the DNA-binding Runt domain, bound to MYC (Fig. 3f and Supplementary Fig. 4b). A GST pulldown assay using purified recombinant GST-Runt fusion protein and recombinant full-length MYC protein further showed that RUNX3 bound directly to MYC (Fig. 3g). Since the Runt domain is highly conserved among human RUNX paralogs, we checked whether RUNX1 and RUNX2 interacted with MYC. Full-length RUNX1, 2 and 3 were individually co-expressed with MYC in HEK293T cells. Immunoprecipitation revealed that RUNX1 and 2 also bound MYC (Supplementary Fig. 4c), indicating that MYC binding is a conserved feature of human RUNX family members. Transfection of RUNX3 (aa 1–187) into HGC27 cells resulted in strong depletion of MYC protein, suggesting that the Runt domain alone is necessary and sufficient for MYC degradation (Supplementary Fig. 4d).

### RUNX3 interacts with GSK3β and FBXW7 to promote ubiquitin-proteasomal degradation of MYC

**Fig. 4:**
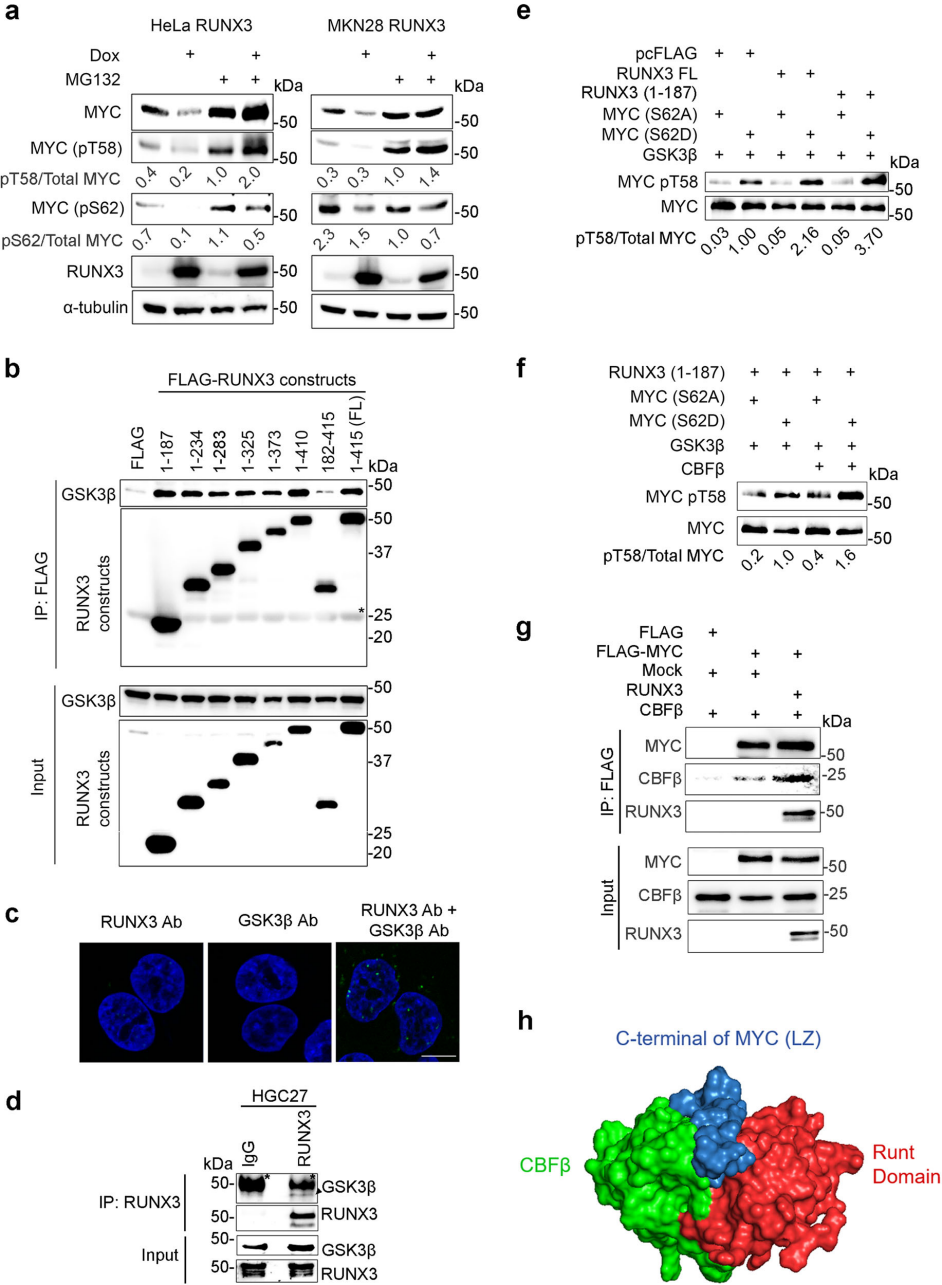
RUNX3 interacts with GSK3β to promote GSK3β-mediated phosphorylation at Threonine 58. **a** Immunoblot of HeLa-RUNX3 and MKN28-RUNX3 cells treated with Dox and/or MG132 (as indicated). α-tubulin is the loading control. Relative MYC phosphorylation at T58 and S62 were calculated by the densitometric values of pT58 and pS62 versus total MYC. The data is representative of two independent experiments. **b** Immunoprecipitation (IP) of HEK293T transfected with FLAG-tagged RUNX3 constructs and GSK3β expression vector. FL denotes full-length. Immunoblot was performed using anti-FLAG and anti-GSK3β antibodies. Asterisk indicates FLAG antibody band. Input is 5% of amount used in IP. The data is representative of three independent experiments. **c** Proximity ligation assay (PLA) of HGC27 cells using anti-RUNX3 and anti-GSK3β antibodies (Ab). Green signals represent endogenous RUNX3 and GSK3β proteins in close proximity. DAPI (blue) indicates nucleus. Scale bar, 10 µm. The data is representative of three independent experiments. **d** Co-immunoprecipitation of endogenous proteins from HGC27 cell lysate. Anti-RUNX3 antibody is used for precipitation. IgG is the control. Arrowhead indicates GSK3β protein that is co-precipitated with RUNX3. Asterisk indicates antibody band. The data is representative of three independent experiments. **e**, **f** In vitro GSK3β kinase assay was performed by adding recombinant GSK3β to in vitro translated proteins from the indicated plasmids. pcFLAG denotes FLAG empty vector (negative control). Samples are subjected to HA affinity IP before SDS-PAGE and immunoblot. Relative MYC phosphorylation at T58 was determined by the densitometric values of pT58 compared to total MYC. The data is representative of two independent experiments. **g** IP and immunoblot of HEK293T cells transfected with the indicated plasmids. The data is representative of two independent experiments. **h** ClusPro server prediction of the three-dimensional structure for complex containing Runt domain/CBFβ and the bHLH LZ domain of MYC.

We had earlier shown that mutations T58A and S62A in MYC resulted in abolishment of RUNX3’s ability to destabilize MYC protein. Using MG132 to inhibit proteasomal degradation of MYC, we observed that T58 phosphorylation of MYC was increased 2-fold in Dox-treated HeLa-RUNX3 and 1.4-fold in Dox-treated MKN28-RUNX3, when compared to non-Dox-treated cells (Fig. 4a). Yet, RUNX3 bound to MYC mutants T58A, T58D, S62A and S62D with similar affinities to wild-type MYC (Supplementary Fig. 4e), suggesting that the phosphorylation status of T58 and S62 did not affect RUNX3 interaction with MYC. Since GSK3β phosphorylates S62-phosphorylated MYC at T58, we next ascertained the relationship of RUNX3 with GSK3β. Immunoprecipitation studies in HEK293T cells showed that the N-terminus of RUNX3 (aa 1–187) bound to GSK3β (Fig. 4b). Furthermore, interaction of endogenous RUNX3 and endogenous GSK3β in HGC27 cells were observed by proximity ligation assay and co-immunoprecipitation (Fig. 4c, d). In vitro kinase assays using a combination of recombinant GSK3β, its MYC substrate phosphomimetic S62D and negative control S62A, and full-length RUNX3 or RUNX3 (aa 1–187) showed that RUNX3 and RUNX3 (aa 1–187) enhanced the ability of GSK3β to phosphorylate MYC (S62D) at T58 by 2.16 and 3.70 times respectively (Fig. 4e). Moreover, GSK3β-mediated phosphorylation of T58 was further accentuated by 1.6-fold when CBFβ was included (Fig. 4f). It would seem that the Runt domain, in complex with CBFβ, tethers MYC to GSK3β for effective phosphorylation. Indeed, co-immunoprecipitation assays revealed that the MYC-CBFβ interaction is enhanced by RUNX3 overexpression (Fig. 4g).

The crystal structure of RUNX1 Runt domain/CBFβ complex bound to DNA has been reported^26,28^. The crystal structures of MYC:MAX bHLH-LZ complex bound to DNA and in its absence have also been reported^41–43^. Moreover, MYC has been shown to interact with CBFβ^44^. We therefore used the computational docking tool ClusPro server (https://cluspro.org) to generate a feasible interpretation of our Runt-CBFβ-bHLH-LZ interaction studies. We show here one of the top 2 predicted low energy docking three-dimensional structures for the ternary complex containing the Runt domain, CBFβ and the bHLH LZ domain of MYC (Fig. 4h).

**Fig. 5:**
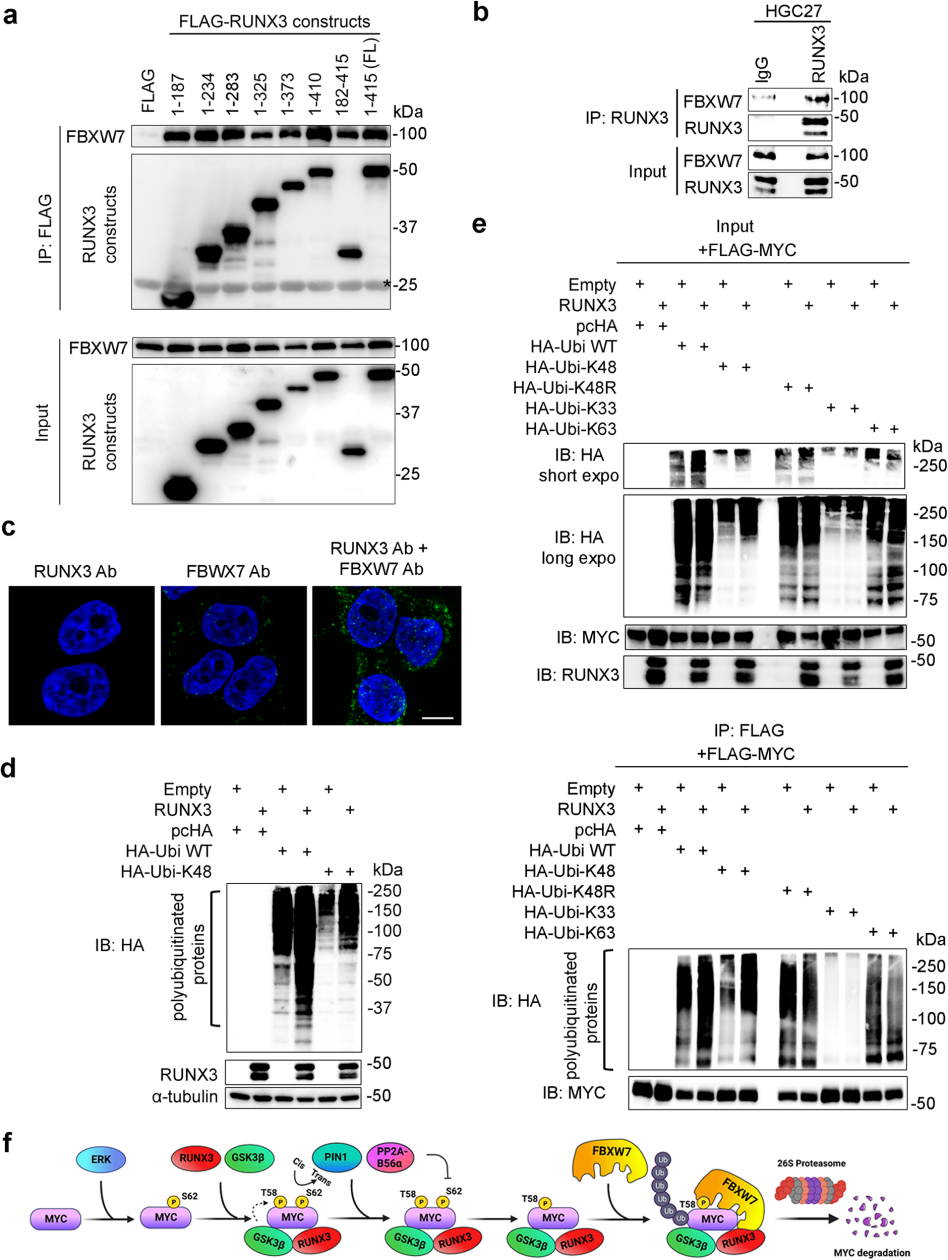
RUNX3 enhances FBXW7-mediated ubiquitin-proteasomal degradation of MYC. **a** Immunoprecipitation (IP) of HEK293T cells transfected with plasmids encoding FBXW7 and FLAG-tagged RUNX3 constructs. Input is 5% of amount used in IP. Asterisk indicates antibody bands. The data is representative of three independent experiments. **b** Co-immunoprecipitation of endogenous RUNX3 and FBXW7 from HGC27 cell lysate. Anti-RUNX3 antibody is used for precipitation. IgG is the control. The data is representative of three independent experiments. **c** Proximity ligation assay (PLA) of HGC27 cells using anti-RUNX3 and anti-FBXW7 antibodies (Ab). Green signals represent endogenous RUNX3 and FBXW7 proteins in close proximity. DAPI (blue) indicates nucleus. Scale bar, 10 µm. The data is representative of two independent experiments. **d** Ubiquitination levels were assessed in HEK293T cells transfected with indicated combinations of RUNX3, HA-tagged wild-type ubiquitin (HA-Ubi WT), and its K48 mutant counterpart (HA-Ubi-K48) constructs. Immunoblot was performed with anti-HA, anti-RUNX3 and anti-α-tubulin antibodies. The data is representative of two independent experiments. **e** IP of HEK293T cells co-transfected with FLAG-MYC and various ubiquitin constructs. For immunoblot of input (5% of lysate used for IP), long and short expo denote long and short exposure during chemiluminescence. For IP using FLAG affinity beads, Empty refers to the empty vector control added when RUNX3 is not overexpressed. IB denotes immunoblot. The data is representative of two independent experiments. **f** Proposed model of how RUNX3 regulates MYC proteasomal degradation. Created with BioRender.com.

We observe that FBXW7 interacts with RUNX3 but were unable to pinpoint the interface; both the N-terminus and C-terminus regions of RUNX3 bound to FBXW7 (Fig. 5a). Interaction of endogenous RUNX3 and FBXW7 were observed by proximity ligation and co-immunoprecipitation assays (Fig. 5b, c). To ascertain whether RUNX3 promotes ubiquitination, we performed an in vivo ubiquitination assay. HEK293T cells were co-transfected with indicated combinations of RUNX3, HA-tagged ubiquitin. Since K48-linked polyubiquitination is a signal for proteasome degradation, we included the ubiquitin K48 construct (i.e. ubiquitin with only lysine-48, other lysines mutated to arginines), which allows only ubiquitin conjugation via K48 linkage. Western blot analysis with anti-HA antibody revealed increased amounts of ubiquitinated products (ranging from 37 to 250 kD) when RUNX3 was introduced (Fig. 5d). Moreover, the K48-linked ubiquitinated proteins were substantially increased in the presence of RUNX3, suggesting that RUNX3 promoted the formation of ubiquitinated products, which were targeted for proteasomal degradation (Fig. 5d). Next, we co-expressed FLAG-MYC, RUNX3 with various combinations of HA-tagged mutant ubiquitin K48R (K48 mutated to R), K63 (ubiquitin with only K63) and K33 (ubiquitin with only K33) (Fig. 5e, see input). The lysates were boiled in 1% SDS and diluted in 0.1% SDS before immunoprecipitation to ensure that the FLAG-affinity pulldown consisted mainly of MYC. While the presence of RUNX3 increased K48-ubiquitination of MYC, RUNX3 was unable to promote K63- and K33-ubiquitination of MYC. Accordingly, the K48R ubiquitin mutant abolished the ability of RUNX3 to promote MYC ubiquitination (Fig. 5e, see IP).

Taken together, our data indicate the scenario where CDK/ERK phosphorylation of MYC at S62 promotes RUNX3-GSK3β cooperation in the phosphorylation of MYC at T58 (Fig. 5f). Following subsequent actions by PIN1 and PP2A-B56α, RUNX3 facilitates FBXW7-mediated ubiquitination of MYC, which results in its proteasomal degradation (Fig. 5f). Intriguingly, PIN1 has previously been shown to interact and mediate ubiquitin-degradation of RUNX3^45^. It is unclear how this interaction affects the RUNX3-MYC relationship. Nevertheless, the multiple interactions of RUNX3 with components of the MYC-degradation pathway indicate the multi-level regulation of MYC activity by RUNX3.

### RUNX3 and Omomyc compete for binding to the bHLH-LZ domain of MYC

**Fig. 6:**
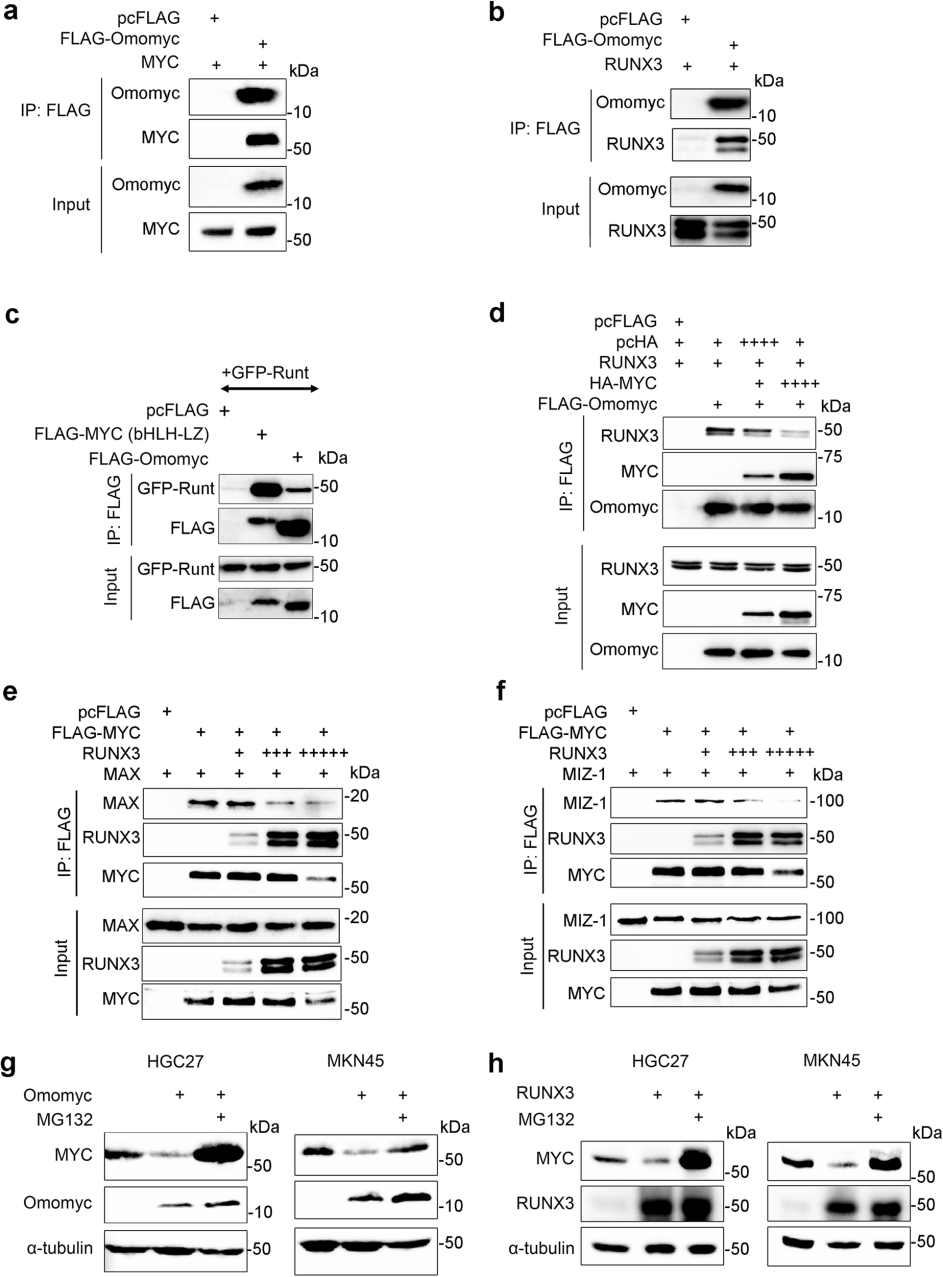
RUNX3 and Omomyc compete for binding to the bHLH-LZ domain of MYC. **a** Immunoprecipitation (IP) of lysates from HEK293T cells transfected with the indicated plasmid combinations. The data is representative of two independent experiments. **b** IP of lysates from HEK293T cells transfected with indicated plasmid combinations. Untagged RUNX3 was detected by anti-RUNX3 antibodies. The data is representative of two independent experiments. **c** IP of lysates from HEK293T cells transfected with the indicated plasmids. GFP-Runt refers to GFP-tagged RUNX3 aa 1–187. The data is representative of two independent experiments. **d** IP of lysates from HEK293T cells transfected with indicated plasmids for 24 h. + and ++++ represent 1 µg and 4 µg DNA respectively. The data is representative of two independent experiments. **e**, **f** IP of lysates from HEK293T cells transfected with the indicated plasmids for 24 h. +, +++, +++++ represent 1 µg, 4 µg, and 7 µg DNA respectively. The data is representative of two independent experiments. **g** Immunoblot of cells transfected with pcFLAG (empty vector control) or FLAG-Omomyc for 42 h, followed by addition of MG132 for another 6 h. The data is representative of two independent experiments. **h** Immunoblot of cells transfected with pcFLAG (empty vector control) or FLAG-RUNX3 for 42 h, followed by addition of MG132 for another 6 h. The data is representative of two independent experiments.

The ability of RUNX3 to bind to the bHLH-LZ domain of MYC is reminiscent of the MYC/Omomyc interaction. Interestingly, similar to the Omomyc/MYC interaction, RUNX3 bound to Omomyc (Fig. 6a, b). Moreover, the Runt domain of RUNX3 is responsible for interaction with Omomyc (Fig. 6c). We therefore compared the interactions of RUNX3 and Omomyc with MYC. HA-tagged MYC, FLAG-tagged Omomyc and RUNX3 were co-transfected into HEK293T cells at various combinations. Increasing MYC concentration, resulted in increase in MYC/Omomyc complex and a corresponding decrease in RUNX3/Omomyc complex formation, suggesting that RUNX3 and MYC bound to the 90 amino acid Omomyc in a mutually exclusive manner (Fig. 6d).

The bHLH-LZ domain is responsible for MYC heterodimerization with MAX and ZBTB17 (also known as MIZ-1)^46,47^. Moreover, Omomyc is known to disrupt the MYC/MAX complex, but not the MYC/MIZ-1 complex^20,48^. We therefore examined the effects of RUNX3 on the MYC/MAX complex and the MYC/MIZ-1 complex. RUNX3, MAX, MIZ-1 and FLAG-tagged MYC were introduced into HEK293T cells at the indicated combinations for co-immunoprecipitation assays. FLAG affinity pull-down of MYC revealed that RUNX3 disrupted the MYC/MAX complex and the MYC/MIZ-1 complex in a concentration-dependent manner (Fig. 6e, f). At the highest concentration of RUNX3 tested, total MAX and MIZ-1 protein levels remained constant while MYC degradation becomes apparent, which suggests that RUNX3 first disrupts MYC/MAX and MYC/MIZ-1 interactions, before destabilizing MYC (Fig. 6e, f). We next investigated the interaction of RUNX3 with MAX and MIZ-1. RUNX3, MYC (bHLH-LZ), MAX and MIZ-1 were introduced into HEK293T cells at the indicated combinations. As expected, MAX co-precipitated with Omomyc and MYC; RUNX3, however, failed to bind MAX (Supplementary Fig. 5a). Conversely, RUNX3 interacted with MIZ-1 via the Runt domain (Supplementary Fig. 5b). Co-immunoprecipitation showed interaction of endogenous RUNX3 and MIZ-1 in HGC27 cells (Supplementary Fig. 5c). Proximity ligation assay revealed endogenous RUNX3/MIZ-1 complex in the nuclei of HGC27 cells (Supplementary Fig. 5d). The absence of RUNX3/MAX complex in the proximity ligation assay confirmed that RUNX3 and MAX do not interact (Supplementary Fig. 5d). Similar to Omomyc overexpression, RUNX3 overexpression in HGC27 and MKN45 cell lines, led to reduced MYC protein levels, which could be rescued by proteasome inhibitor MG132 (Fig. 6g, h). It would seem that RUNX3 inhibits MYC-dependent transactivation by (1) disrupting MYC heterodimerization with MAX and MIZ-1 and (2) promoting its degradation.

Omomyc was previously shown to exert a strong dominant negative effect on MYC promoting *Ras*-induced lung adenocarcinoma development^20^. *Runx3* knockout mouse models showed RUNX3 to be a critical barrier for adenoma to adenocarcinoma progression in the intestine and lung^29,30^. Although our earlier interpretations were that RUNX3 attenuates Wnt signaling and induces p53 to restrain oncogenic Ras signaling, the data presented here suggest that RUNX3 mimics part of Omomyc’s activities, including disruption of MYC dimerization domain and enhancement of MYC degradation, to impede MYC’s oncogenic drive in the lung and intestine. The fact that RUNX3 has the ability to regulate MYC at two different levels – through attenuation of Wnt signaling and MYC protein destabilization – may explain why RUNX3 functions as a potent tumor suppressor in the intestine and lung.

We compared RUNX3 and MYC protein levels in human gastric tumor tissue arrays by immunohistochemistry (IHC) (Supplementary Fig. 6). The lymphocytes, which are known for strong expression of RUNX3 relative to epithelial cells^49,50^, showed strong nuclear RUNX3 staining, and thus served as internal positive controls (Supplementary Fig. 6a, d, see lymphoid aggregates). As expected, the tumor cells typically showed weak or no RUNX3 protein staining. The rare tumor cell that exhibited moderate nuclear RUNX3 expression coincided with very weak or no discernible MYC protein expression (Supplementary Fig. 6b, e). Conversely, tumor cells that showed high nuclear MYC expression stained poorly for RUNX3 (Supplementary Fig. 6c, f). Within a tumor core (Supplementary Figs. 6 and 7), where cells showed a range of RUNX3 and MYC expression levels, the inverse correlation of RUNX3 and MYC expression was clearly evident. Although we were unable to achieve statistical significance due to the low frequency of RUNX3-expressing tumors, our findings indicate a trend of inverse correlation between RUNX3 and MYC protein levels in gastric tumor (Supplementary Fig. 7).

## Discussion

*Runx* and *Myc* genes have been identified in one of the closest unicellular ancestors of Metazoa, *Capsaspora owczarzaki*^51^. The distribution of *Myc* and *Runx* binding motifs in the *Capsaspora* genomic landscape suggest that both proteins belong to an ancient transcription factor network with regulatory interactions in proliferation^52^. In addition, *Capsaspora Myc* was inferred to regulate ribosomal biogenesis, similar to its function in animals^52,53^. Although ribosomal biogenesis does not seem to be downstream of *Capsaspora Runx*^52^, mammalian Runx proteins were reported to repress ribosomal RNA synthesis^54,55^. Runx proteins remain associated with condensed chromosomes during mitosis and are proposed – through lineage-specific regulation of ribosomal biogenesis – to serve as an epigenetic link between cell fate and proliferation^54,55^. The functional overlaps of RUNX and MYC suggest that an ancient MYC-RUNX relationship in proliferation was further expanded to include ribosomal biogenesis in the advent of the multicellular Metazoa.

In mammals, MYC is profoundly involved in the transcriptional regulation of key cell growth programs such as cell cycle progression, metabolism, mitochondrial and ribosomal biogenesis^12^. In stem cells, MYC regulates self-renewal and proliferation and has been shown to inhibit terminal differentiation in various cell types^1^. A recent paper showed that acute Myc activation in indolent pancreatic intraepithelial neoplasm epithelial cells induced changes in stromal and immune-cell types, thereby promoting pancreatic adenocarcinoma development^13^. While these properties earned MYC the epithet of principal orchestrator of tumor growth, they also render MYC addiction in various cancers. Indeed, targeting the transcriptional activity of MYC strongly inhibits tumor growth^14^. Omomyc, one of the most effective MYC inhibitors, strongly promotes tumor regression^23^. Small molecule inhibitors (eg. MYCi361) that disrupt MYC/MAX heterodimerization were shown to enhance proteasome-mediated MYC degradation by promoting MYC phosphorylation at threonine-58^22^.

It is interesting that RUNX3 partially mimics the MYC-destabilizing effects of Omomyc. Moreover, similar to MYCi361, RUNX3 also promotes MYC phosphorylation at T58 and accelerates MYC degradation. A wealth of evidence shows that RUNX3 exerts strong growth inhibition in epithelial cells. Moreover, *Runx3* deficiency is associated with elevated stem cell population and impaired terminal differentiation in epithelial cells^32,56^. Another interesting aspect of RUNX3 function is its role in lineage-specific differentiation of innate lymphoid cells^57,58^. It would seem that MYC and RUNX3 converge on similar cellular programs to serve opposite roles. RUNX3 might arguably be evolution’s answer to aberrant MYC activity – nature’s very own Omomyc. In view that the MYC/MAX/MIZ-1 complex suppresses the transcription of growth inhibitor *CDKN1A*^59,60^, the additional ability of RUNX3 to disrupt the MYC/MIZ-1 interaction gives RUNX3 an advantage over Omomyc. Both *RUNX3* and *MIZ-1* genes reside within chromosome *1p36*, a region which is frequently deleted in cancer and which has been postulated to contain several tumor suppressors that work together^61,62^. Moreover, RUNX3 cooperates with the TGFβ pathway to upregulate *CDKN1A* transcription^63^. The functional significance of the RUNX3/MIZ-1 complex in *CDKN1A* regulation, while unclear, is an intriguing avenue for further studies.

The fact that RUNX3 is frequently hypermethylated in cancer begs the question: will reactivation of the silenced *RUNX3* to stem aberrant MYC activity? An obvious caveat is that the frequent mutation of *FBXW7* in cancer^64^ might derail RUNX3’s ability to promote MYC protein degradation. Moreover, one of the *MYC* hotspot mutations is T58A (http://cancer.sanger.ac.uk)^65–67^. These mutations raise yet another question: what lies downstream of a stable RUNX3/MYC complex? Further in-depth studies of the RUNX3-MYC relationship will improve our understanding of the interplay between RUNX family members and MYC in cancer biology.

RUNX3 was earlier shown to promote ubiquitination of MYCN in neuroblastoma and the Hedgehog pathway oncogenic transcription factor GLI1 in colorectal cancer cells^33,68^. RUNX3 was also reported to reduce the stability of the estrogen receptor α protein in breast cancer cells, although the mechanism remains unclear^69^. Taken together, our data suggest that aside from its well-established role as a transcription factor, RUNX3 plays an additional role – not less prominent than its canonical one – in inhibiting oncogenic transcription factor function. Earlier, we showed that RUNX3 interferes with the DNA binding domains of TCF4 and TEAD4^29,70^. Here, we show that not only did RUNX3 interfere with MYC/MAX function, it also promotes MYC degradation. The fact that the RUNX3-targeted proteins identified so far are transcription factors involved in oncogenic pathways tellingly illustrates RUNX3’s tumor suppressor role. Conceivably, the ability of one transcription factor to destabilize another is an efficient way to rapidly effect transcriptional change.

## Methods

### Cell lines, transfection, and siRNA knockdown

The Hela-Tet-On cell line was purchased from Clontech. The MKN28 Tet-On cell line was generated by transfection of pRetroX-Tet-On Advanced Vector (Clontech) into MKN28 and subsequent selection with G418 (1000 µg/ml). RUNX3 and its mutant counterpart R122C were cloned into pRetroX-Tight-puro-3xFLAG to generate Dox-inducible RUNX3 expression constructs^35^. The constructs were then transfected into the Tet-On cell lines and selected with 0.5 µg/ml puromycin. Expression of RUNX3 and R122C mutant were induced by 0.5 µg/ml doxycycline (Sigma). HGC27, MKN28, and AGS cell lines were maintained in RPMI-1640 media supplemented with 10% fetal bovine serum (FBS). HEK293T and MKN45 were cultured in Dulbecco’s modified Eagle’s medium (DMEM) containing 10% FBS. HeLa- and MKN28-Dox-inducible cell lines were cultured in DMEM and RPMI-1640 supplemented with 10% tetracycline-free FBS respectively. Penicillin/streptomycin (100 U/ml) was added to all cell culture media. Cells were cultured at 37 °C in a humidified 5% CO_2_ incubator. All cell lines were authenticated before usage and checked for mycoplasma contamination every 3 months using LookOut® Mycoplasma qPCR Detection Kit (Sigma Aldrich). Plasmids were transfected using TransIT®-LT1 Transfection Reagent (Mirus), siRNA transfection was performed at a concentration of 35 nM using jetPRIME® transfection reagent (Polyplus) for 48 h, according to manufacturer protocol. To study if RUNX3 induce MYC degradation, cells were treated with 10 µM MG132 (Sigma) for 6 h before the lysates were collected and analyzed by immunoblot. Cells were pre-treated with doxycycline where indicated.

### Antibodies and reagents

Details on antibodies, reagents, software/algorithms are provided in Supplementary Table 1.

### Immunoblot

Cells were lysed with RIPA buffer supplemented with protease and phosphatase inhibitor. Extracted proteins were quantified using Pierce BCA assay kit (ThermoFisher Scientific). In all, 40 µg of total protein from each sample were subjected to SDS polyacrylamide gel electrophoresis (PAGE) and electrophoretic transfer to 0.2 µm polyvinylidene difluoride (PVDF) membrane (Bio-Rad) using the Mini Trans-Blot cell (Bio-Rad) at 30 V, 100 mA, 4 °C overnight. The transfer buffer comprises 25 mM Tris, pH 8.3, 192 mM glycine, with 0.03% SDS. The membrane was subsequently blocked in 5% skimmed milk (Nacalai) for total protein immunoblot or 5% BSA (Sigma) for phospho-protein immunoblot. Blots were then incubated with primary antibodies (1:200 for Santa Cruz antibodies and 1:1000 for other antibodies) in appropriate blocking solution at 4 °C overnight, followed by 1 h incubation with horseradish peroxidase conjugated secondary antibody (1:5000) at room temperature. After copious washes with PBS-T (PBS supplemented with 0.1% Tween 20), antibody detection was performed using Radiance plus chemiluminescent substrate (azurebiosystems) and visualized by ChemiDoc MP system (Bio-Rad).

### Immunoprecipitation

HEK293T cells (1.5 × 10^6^) were seeded on 10-cm dishes and transfected with indicated plasmids on the next day. At 24 h post-transfection, cells were collected and lysed using IP lysis buffer [50 mM Tris pH 8.0, 0.3 M NaCl, 0.5 mM EDTA, 5% glycerol, 0.5% NP40, supplemented with 1 mM DTT, PMSF, Benzonase® Nuclease (Merck Millipore), cOmplete™ protease inhibitor cocktail (Roche) and 1x Halt™ phosphatase inhibitor (ThermoScientific)]. Protein concentration were then quantified using Bradford reagent (Bio-Rad) 20 µg of the extracted protein were used for input lane. 400 µg of proteins were then incubated in incubation buffer [25 mM Tris pH 8.0, 0.15 M NaCl, 0.25 mM EDTA, 2.5% glycerol, 0.25% NP40, 1 mM DTT, supplemented with the above protease and phosphatase inhibitors] and subjected to immunoprecipitation for 90 min with EZview ™ Red anti-HA affinity gel (SigmaAldrich) for HA-tag pull down or Flag M2 monoclonal antibody affinity gel for Flag pulldown. The affinity gels were then washed several times with wash buffer [25 mM Tris pH 8.0, 0.15 M NaCl, 0.25 mM EDTA, 2.5% glycerol, 0.25% NP40, and 1 mM DTT] and bound proteins were eluted by boiling in Laemmli buffer. Bound proteins were then analyzed by SDS-PAGE and western blot analysis. For immunoprecipitation of endogenous proteins, HGC27 cells were lysed in IP lysis buffer. 1 mg of whole-cell lysate was incubated in incubation buffer with anti-RUNX3 (1:200) (Cell Signaling Technology, D9K6L) antibody or mouse normal IgG (1:100) (Santa Cruz sc-2025) overnight at 4 °C, followed by addition of Dynabeads™ Protein G (Thermo Fisher Scientific) for 3 h at 4 °C. The beads were copiously washed and subjected to western blot analysis.

### Ubiquitination assay

HEK293T cells were transfected with the indicated plasmids and HA-tagged ubiquitin construct (2 µg each) for 24 h. Cells were then harvested and lysed by RIPA buffer for immunoblot with HA antibody to assess the level of total protein ubiquitination. To specifically assess MYC ubiquitination levels, the cells were co-transfected with FLAG-MYC and HA-ubiquitin plasmids. The cell lysates were boiled in lysis buffer with 1% SDS for 30 min. The lysates were then diluted to 0.1% SDS and subjected to FLAG affinity purification. The FLAG-MYC immunoprecipitates were resolved by SDS-PAGE and the covalent attachment of ubiquitin to FLAG-MYC determined by immunoblot using anti-HA antibodies (1:1000).

### Proliferation assay

Cells were plated (4 × 10^3^ cells per well) onto 96-well flat-bottom plates in triplicates. Cells were trypsinized, and counted each day using hemocytometer. Drug treatments or siRNA transfection were performed as indicated. Assays were performed independently at least three times. Statistical analysis was performed with Student’s *t* test (two-tailed).

### Immunofluorescence staining

Cells were seeded onto sterile glass coverslips placed in six-well-plates overnight. Doxycycline was added where indicated for 24 h. Cells were fixed with 4% paraformaldehyde for 30 min at room temperature, permeabilized with 0.25% Triton X-100 for 30 min and blocked with 3% BSA for 60 min at room temperature. The samples were then incubated with primary antibodies (1:400) at 4 °C overnight. Excess antibodies were removed by copious PBS washes. The samples were then incubated with Highly Cross-Adsorbed Secondary Antibody, Alexa Fluor™ 488, 555 and 546 (1:400) (Invitrogen) at room temperature for 60 min. Following PBS washes, the cells were stained with DAPI (4′,6-diamidino-2-phenylindole) as a counterstain for nuclei, before being mounted with ProLong™ Diamond Antifade mountant (Invitrogen) onto glass slides. Samples were visualized with Zeiss LSM880 Airy Scan confocal microscope and analyzed with Zeiss Zen (Blue) imaging software. For EdU staining, Click-iT EdU Cell Proliferation Kit for Imaging (Invitrogen) was used according to manufacturer’s instructions.

### Proximity ligation assay

Proximity ligation assay (PLA) was performed using Duolink^TM^ In Situ Probe Anti-Mouse PLUS, Anti-Rabbit MINUS, Wash Buffers (Fluorescence), Reagents Green, and Mounting medium with DAPI, according to manufacturer’s instructions (Sigma-Aldrich). Primary antibodies combinations used were anti-RUNX3 rabbit (1:400) (Cell Signaling Technology, D6E2) and anti-MYC mouse (1:400) (OriGene, OTI3F2) monoclonal antibodies; anti-RUNX3 (1:400) (Cell Signaling Technology, D9K6L) and anti-MYC (1:400) (Cell Signaling Technology, D84C12); anti-RUNX3 (1:400) (Cell Signaling Technology, D9K6L) and anti-GSK3B (1:400) (Cell Signaling Technology, D5C5Z); anti-RUNX3 (1:400) (Cell Signaling Technology, D9K6L) and FBXW7 (1:400) (Proteintech, 55290-1-AP); anti-RUNX3 (1:400) (Cell Signaling Technology, D9K6L), and MAX (1:400) (abcam, ab199489); RUNX3 (1:400) (Cell Signaling Technology, D9K6L) and MIZ-1 (1:400) (Cell Signaling Technology, D7E8B). The signals were visualized with Zeiss LSM880 confocal microscope and analyzed with Zeiss Zen (Blue) imaging software.

### RNA sequencing

HeLa-RUNX3 and MKN28-RUNX3 were treated with 500 ng/ml Doxycycline for 48 h. Untreated HeLa-RUNX3 and MKN28-RUNX3 served as control (mock). Total RNA was extracted using RNeasy mini kit with DNase treatment (QIAGEN) and diluted in RNAse-free water. Quality control of RNA was performed by Agilent Bioanalyzer 2100 (Agilent Technologies). RNA samples were sent to BGI Genomics for preparation of transcriptome library and sequencing by the DNBseq™ next generation platform. Clean reads were mapped to the human reference genome hg19 using HISAT2. Data analysis was performed by the online automated platform CSI NGS Portal^71^. Analysis of differential gene expression was done with DESeq2 and Broad Institute Gene Set Enrichment Analysis (GSEA) tool with the Molecular Signature Database v6.0^72^.

### RNA isolation and real-time quantitative PCR

Cells were harvested and homogenized using QIAshredder (QIAGEN). Total RNA was isolated using RNeasy mini kit with DNase treatment (QIAGEN). cDNA synthesis was performed using High-Capacity cDNA Reverse Transcription Kit (Applied Biosystems). Quantitative PCR (qPCR) was conducted using iTaq™ Universal SYBR® Green Supermix (Biorad) on QuantStudio™ 3 Real-Time PCR System (Applied Biosystems). Gene expression was normalized to the GAPDH mRNA levels.

### Generation of RUNX3 and MYC point mutations and truncations

All point mutations and short truncations were generated using KAPA HiFi PCR Kit (KAPA Biosystems) using primers containing the desired mutations. The PCR product was digested with DpnI (NEB) for 3 h before transformation into competent STBL3 bacteria cells. After transformation, the bacteria were selected with 100 µg/ml ampicillin. Plasmids with mutated sequences were confirmed by DNA sequencing (1^st^ BASE, Singapore). Other truncation constructs were generated by PCR-cloning using oligonucleotides (see Resources Table, primers designated with T4).

### GST pull-down assay

pGEX4T-1 plasmids containing glutathione‐S‐transferase (GST) and GST-tagged Runt domain (RUNX3 aa 49–187) were expressed and purified from *E. coli* Rosetta^TM^ DE3 cells after induction with 0.1 mM IPTG for 3 h at 37 °C. The GST proteins were purified by glutathione (GSH) Sepharose 4B beads (GE Healthcare). For the pulldown assay, GST proteins bound to GSH beads were incubated with 5 µg of recombinant MYC (RayBiotech). After copious washes, the precipitated proteins were resolved by SDS-PAGE and analyzed by immunoblot using anti-MYC (1:1000) and anti-GST (1:200) antibodies.

### Analysis of MYC protein half-life by cycloheximide treatment

HeLa Tet-On and MKN28 Tet-On cell lines were treated with 500 ng/ml and 20 ng/ml doxycycline, respectively, for 12 h before addition of 50 μg/ml cycloheximide (Sigma-Aldrich). Cells were harvested at every 15 min interval, lysed and analyzed by immunoblotting. Plasmid transfections were performed 24 h before cycloheximide-treatment where indicated. The densitometry of protein band intensity was performed using ImageJ software.

### In vitro protein kinase reaction

FLAG empty vector control, FLAG-tagged-RUNX3 and FLAG-tagged RUNX3 truncation (aa 1–187) as well as HA-tagged wild-type MYC and mutant counterparts S62A and S62D were in vitro translated using TNT® T7 Quick Coupled Transcription/Translation system (Promega) using 1 μg of the respective plasmid DNA. After incubation for 90 min at 30 °C, the samples were treated with 10 units DNase I (Roche) for 30 min at room temperature. Kinase reaction was performed by incubating the in vitro translated proteins with 1x PK buffer (NEB), 5 mM ATP and 2 µl recombinant human GSK3 beta protein (Active) (Abcam) at 30 °C for 30 min. 2 µl recombinant CBFβ protein (Abcam) was added where indicated. The samples were subjected to immunoprecipitation using EZview ™ Red anti-HA affinity gel (Sigma Aldrich) before SDS-PAGE and western blot analysis.

### Tumoroid formation assays

HeLa-RUNX3 (2000 cells) were trypsinized and resuspended in 120 µl matrigel (Discovery Labware) and seeded in 24 well-plates (6 replicates/sample). Dox (500 ng/ml) is added where indicated during resuspension. After 30 min incubation in 37 °C CO_2_ incubator, 600 µl culture media (supplemented with Dox 500 ng/ml where indicated) was added. Media was changed every 3 days. Photographic images of tumoroids were acquired at day 6.

### Assessment of cell growth in suspension with no cell attachment

HeLa-FLAG, -RUNX3, and -R122C cells were seeded at 3000 cells / 200 µl media per well on Nunclon™ Sphera™ 96-well low attachment plates (ThermoScientific). Media were replenished once every 3 days. Dox (500 ng/ml) was added where indicated. Cells were photographed on Day 7 after seeding.

### Tumorigenesis assay in NOD scid gamma mice

MKN-28 control and MKN28-RUNX3 Dox-inducible cells (2.5 × 10^5^ cells per injection) were resuspended in 40 μL of Matrigel and subcutaneously injected into 8 to 10-week-old male NOD scid gamma (NSG) mice (The Jackson Laboratory) at the left and right flanks respectively. Subcutaneous implantations were performed under general anesthesia using isoflurane (Baxter). Mice were fed with doxycycline-embedded food pellets (Envigo) for the entire duration of the experiment. At the end of the experiments (12 weeks after cell injection), the recipient mice were sacrificed, and tumor xenografts harvested. Proteins were extracted from the tumor xenografts and used for Western blot analysis. All animal work was performed according to the experimental protocol approved by the Institutional Animal Care and Use Committee and the Office of Safety, Health, and Environment at the National University of Singapore (NUS).

### ClusPro

The simulation of molecular docking was performed on the ClusPro protein-protein docking web server (https://cluspro.bu.edu)^73–75^. For molecular docking, the inputs were taken from the Runt domain from AML1 and CBFB complex (AML1/CBFbeta) complex; (PDB ID: 1E50) and MYC LZ domain (MYC/MAX LZ domain; PDB ID: 1A93)^28,43^. The predicted molecular complex (Fig. 4h) was one of the top three conformations with lowest docking energy structure (ClusPro score: -646.2) ranked by ClusPro.

### Immunohistochemistry of tissue microarray

Commercial tissue-microarray (TMAs) for human gastric cancer ST1001 and ST1001a were purchased from US Biomax, Rockville, Maryland. Formalin-fixed paraffin-embedded (FFPE) gastric cancer tissue specimens were used to construct the TMAs with cores of 1 mm diameter. Consecutive sections of 5 μM thickness were used to compare RUNX3 and MYC expression across stomach cancer tissue cores. For both ST1001 and ST1001a, cores with insufficient tissues were excluded from the final analysis.

Briefly, sections were deparaffinized and rehydrated followed by antigen retrieval either in pH 9.0 EDTA buffer (RUNX3) or pH 7.8 Tris EDTA buffer (MYC). Endogenous peroxides were then blocked followed by incubation with monoclonal primary antibodies RUNX3 (Clone D6E2, Cell Signaling, ready to use) and c-Myc (Clone Y69, Master Diagnostics, ready to use) according to the optimized protocol. Antibodies were then localized using the standard protocols; for RUNX3, Leica Bond DAB Detection kit and Leica Bond III Autostainer were used. For MYC, Ventana Ultra Optiview DAB detection Kit and Ventana Ultra Autostainer were used. Negative controls were performed simultaneously without the incubation with the primary antibody and other procedures were unchanged. Appropriate positive controls were immunostained in each batch of IHC.

For both RUNX3 and MYC, nuclear staining was considered positive. Semi-quantitative assessment was performed and H-score was calculated for all the TMA cores. Intensity was graded as mild (+1), moderate (+2), and strong (+3) while antibody expression was quantified as percentage of positive cells in the gastric tumor areas. Thereafter, H-score was calculated which was intensity times percentage positive tumor cells. The H-score ranged from 0–300.

### Statistics and reproducibility

The number of replicates and times the experiments were performed are stated in the figure legends. Experiments were repeated and reproduced independently as described in the figure legends. GraphPad Prism (version 9.3.1) was used to perform statistical analysis and plot data. Data is presented as mean ± standard deviation, error bars represented standard deviations. Parametric Student’s *t* test (two-tailed) was used to examine the significance between two groups. *p* values less than 0.05 were considered statistically significant. (NS, not significant for *p* > 0.05; *p ≤ 0.05, **p ≤ 0.01, ***p ≤ 0.001, ****p ≤ 0.0001).

### Reporting summary

Further information on research design is available in the Nature Portfolio Reporting Summary linked to this article.

## Supplementary information

Supplementary Information

Description of Additional Supplementary Files

Supplementary Data 1

Supplementary Data 2

Supplementary Data 3

Reporting Summary

## Data Availability

Supplementary Figs. 8–32 contain uncropped and unedited blot/gel images with size markers. The RNAseq data is available as Excel files in Supplementary Data 1 (HeLa-RUNX3) and 2 (MKN28-RUNX3), and at NCBI Gene Expression Omnibus (GEO) under the accession number GSE233777. The source data for the graphs presented here are available as an Excel file in Supplementary Data 3. Plasmids generated in this study have been deposited in Addgene with ID numbers ranging from # 203424–203440 (see Supplementary Table 1). Further information and requests for resources and reagents should be directed to the corresponding author, Yoshiaki Ito (yoshi_ito@nus.edu.sg).

## References

[1] Eilers M, Eisenman RN (2008). Myc’s broad reach. Genes Dev..

[2] Beroukhim R (2010). The landscape of somatic copy-number alteration across human cancers. Nature.

[3] Hnisz D (2013). Super-enhancers in the control of cell identity and disease. Cell.

[4] Baluapuri A, Wolf E, Eilers M (2020). Target gene-independent functions of MYC oncoproteins. Nat. Rev. Mol. Cell Biol..

[5] Dang CV (2012). MYC on the path to cancer. Cell.

[6] Takahashi K, Yamanaka S (2006). Induction of pluripotent stem cells from mouse embryonic and adult fibroblast cultures by defined factors. Cell.

[7] Wong DJ (2008). Module map of stem cell genes guides creation of epithelial cancer stem cells. Cell Stem Cell.

[8] Lourenco C (2021). MYC protein interactors in gene transcription and cancer. Nat. Rev. Cancer.

[9] Land H, Parada LF, Weinberg RA (1983). Tumorigenic conversion of primary embryo fibroblasts requires at least two cooperating oncogenes. Nature.

[10] He TC (1998). Identification of c-MYC as a target of the APC pathway. Science.

[11] Sansom OJ (2007). Myc deletion rescues Apc deficiency in the small intestine. Nature.

[12] Dang, C. V. MYC, metabolism, cell growth, and tumorigenesis. *Cold Spring Harb. Perspect. Med.*10.1101/cshperspect.a014217 (2013). 10.1101/cshperspect.a014217 PMC3721271 23906881

[13] Sodir NM (2020). MYC instructs and maintains pancreatic adenocarcinoma phenotype. Cancer Discov..

[14] Harrington CT, Sotillo E, Dang CV, Thomas-Tikhonenko A (2021). Tilting MYC toward cancer cell death. Trends Cancer.

[15] Grandori C, Cowley SM, James LP, Eisenman RN (2000). The Myc/Max/Mad network and the transcriptional control of cell behavior. Annu. Rev. Cell Dev. Biol..

[16] Blackwood EM, Eisenman RN (1991). Max: a helix-loop-helix zipper protein that forms a sequence-specific DNA-binding complex with Myc. Science.

[17] Das SK, Lewis BA, Levens D (2023). MYC: a complex problem. Trends Cell Biol..

[18] Amati B (1993). Oncogenic activity of the c-Myc protein requires dimerization with Max. Cell.

[19] Amati B (1992). Transcriptional activation by the human c-Myc oncoprotein in yeast requires interaction with Max. Nature.

[20] Soucek L (2008). Modelling Myc inhibition as a cancer therapy. Nature.

[21] Demma, M. J. et al. Omomyc reveals new mechanisms to inhibit the MYC oncogene. *Mol. Cell. Biol.*10.1128/MCB.00248-19 (2019). 10.1128/MCB.00248-19 PMC6817756 31501275

[22] Han H (2019). Small-molecule MYC inhibitors suppress tumor growth and enhance immunotherapy. Cancer Cell.

[23] Mullard A (2022). Climbing cancer’s MYC mountain. Nat. Rev. Drug Discov..

[24] Ito Y, Bae SC, Chuang LS (2015). The RUNX family: developmental regulators in cancer. Nat. Rev. Cancer.

[25] Blyth K, Cameron ER, Neil JC (2005). The RUNX genes: gain or loss of function in cancer. Nat. Rev. Cancer.

[26] Tahirov TH (2001). Structural analyses of DNA recognition by the AML1/Runx-1 Runt domain and its allosteric control by CBFbeta. Cell.

[27] Bravo J, Li Z, Speck NA, Warren AJ (2001). The leukemia-associated AML1 (Runx1)-CBF beta complex functions as a DNA-induced molecular clamp. Nat. Struct. Biol..

[28] Warren AJ, Bravo J, Williams RL, Rabbitts TH (2000). Structural basis for the heterodimeric interaction between the acute leukaemia-associated transcription factors AML1 and CBFbeta. EMBO J..

[29] Ito K (2008). RUNX3 attenuates beta-catenin/T cell factors in intestinal tumorigenesis. Cancer Cell.

[30] Lee YS (2013). Runx3 inactivation is a crucial early event in the development of lung adenocarcinoma. Cancer Cell.

[31] Li QL (2002). Causal relationship between the loss of RUNX3 expression and gastric cancer. Cell.

[32] Douchi D (2022). A point mutation R122C in RUNX3 promotes the expansion of isthmus stem cells and inhibits their differentiation in the stomach. Cell. Mol. Gastroenterol. Hepatol..

[33] Yu F (2014). RUNX3 interacts with MYCN and facilitates protein degradation in neuroblastoma. Oncogene.

[34] Whittle MC (2015). RUNX3 controls a metastatic switch in pancreatic ductal adenocarcinoma. Cell.

[35] Chuang LS (2016). Aurora kinase-induced phosphorylation excludes transcription factor RUNX from the chromatin to facilitate proper mitotic progression. Proc. Natl Acad. Sci. USA.

[36] Yamada C (2010). RUNX3 modulates DNA damage-mediated phosphorylation of tumor suppressor p53 at Ser-15 and acts as a co-activator for p53. J. Biol. Chem..

[37] Farrell, A. S. & Sears, R. C. MYC degradation. *Cold Spring Harb. Perspect. Med.*10.1101/cshperspect.a014365 (2014). 10.1101/cshperspect.a014365 PMC3935390 24591536

[38] Zindy F (1998). Myc signaling via the ARF tumor suppressor regulates p53-dependent apoptosis and immortalization. Genes Dev..

[39] Linggi B (2002). The t(8;21) fusion protein, AML1 ETO, specifically represses the transcription of the p14(ARF) tumor suppressor in acute myeloid leukemia. Nat. Med..

[40] Qi Y (2004). p19ARF directly and differentially controls the functions of c-Myc independently of p53. Nature.

[41] Sammak S (2019). Crystal structures and nuclear magnetic resonance studies of the apo form of the c-MYC:MAX bHLHZip complex reveal a helical basic region in the absence of DNA. Biochemistry.

[42] Nair SK, Burley SK (2003). X-ray structures of Myc-Max and Mad-Max recognizing DNA. Molecular bases of regulation by proto-oncogenic transcription factors. Cell.

[43] Lavigne P (1998). Insights into the mechanism of heterodimerization from the 1H-NMR solution structure of the c-Myc-Max heterodimeric leucine zipper. J. Mol. Biol..

[44] Agrawal P, Yu K, Salomon AR, Sedivy JM (2010). Proteomic profiling of Myc-associated proteins. Cell Cycle.

[45] Nicole Tsang YH (2013). Prolyl isomerase Pin1 downregulates tumor suppressor RUNX3 in breast cancer. Oncogene.

[46] Peukert K (1997). An alternative pathway for gene regulation by Myc. EMBO J..

[47] Bedard M, Maltais L, Montagne M, Lavigne P (2017). Miz-1 and Max compete to engage c-Myc: implication for the mechanism of inhibition of c-Myc transcriptional activity by Miz-1. Proteins.

[48] Savino M (2011). The action mechanism of the Myc inhibitor termed Omomyc may give clues on how to target Myc for cancer therapy. PLoS ONE.

[49] Ebihara T, Seo W, Taniuchi I (2017). Roles of RUNX complexes in immune cell development. Adv. Exp. Med. Biol..

[50] Egawa T, Tillman RE, Naoe Y, Taniuchi I, Littman DR (2007). The role of the Runx transcription factors in thymocyte differentiation and in homeostasis of naive T cells. J. Exp. Med..

[51] Sebe-Pedros A, de Mendoza A, Lang BF, Degnan BM, Ruiz-Trillo I (2011). Unexpected repertoire of metazoan transcription factors in the unicellular holozoan Capsaspora owczarzaki. Mol. Biol. Evol..

[52] Sebe-Pedros A (2016). The dynamic regulatory genome of capsaspora and the origin of animal multicellularity. Cell.

[53] Grandori C (2005). c-Myc binds to human ribosomal DNA and stimulates transcription of rRNA genes by RNA polymerase I. Nat. Cell Biol..

[54] Pande S (2009). Subnuclear targeting of the Runx3 tumor suppressor and its epigenetic association with mitotic chromosomes. J. Cell. Physiol..

[55] Young DW (2007). Mitotic occupancy and lineage-specific transcriptional control of rRNA genes by Runx2. Nature.

[56] Ito K (2011). Loss of Runx3 is a key event in inducing precancerous state of the stomach. Gastroenterology.

[57] Ebihara T (2015). Runx3 specifies lineage commitment of innate lymphoid cells. Nat. Immunol..

[58] Levanon D (2014). Transcription factor Runx3 regulates interleukin-15-dependent natural killer cell activation. Mol. Cell. Biol..

[59] Seoane J, Le HV, Massague J (2002). Myc suppression of the p21(Cip1) Cdk inhibitor influences the outcome of the p53 response to DNA damage. Nature.

[60] Staller P (2001). Repression of p15INK4b expression by Myc through association with Miz-1. Nat. Cell. Biol..

[61] Bagchi A, Mills AA (2008). The quest for the 1p36 tumor suppressor. Cancer Res..

[62] Wiese KE (2013). The role of MIZ-1 in MYC-dependent tumorigenesis. Cold Spring Harb. Perspect. Med..

[63] Chi XZ (2005). RUNX3 suppresses gastric epithelial cell growth by inducing p21(WAF1/Cip1) expression in cooperation with transforming growth factor {beta}-activated SMAD. Mol. Cell. Biol..

[64] Yeh CH, Bellon M, Nicot C (2018). FBXW7: a critical tumor suppressor of human cancers. Mol. Cancer.

[65] Hemann MT (2005). Evasion of the p53 tumour surveillance network by tumour-derived MYC mutants. Nature.

[66] Bahram F, von der Lehr N, Cetinkaya C, Larsson LG (2000). c-Myc hot spot mutations in lymphomas result in inefficient ubiquitination and decreased proteasome-mediated turnover. Blood.

[67] Tate JG (2019). COSMIC: the catalogue of somatic mutations in cancer. Nucleic Acids Res..

[68] Kim BR (2020). RUNX3 suppresses metastasis and stemness by inhibiting Hedgehog signaling in colorectal cancer. Cell Death Differ..

[69] Huang B (2012). RUNX3 acts as a tumor suppressor in breast cancer by targeting estrogen receptor alpha. Oncogene.

[70] Qiao Y (2016). RUNX3 is a novel negative regulator of oncogenic TEAD-YAP complex in gastric cancer. Oncogene.

[71] An, O. et al. CSI NGS portal: an online platform for automated ngs data analysis and sharing. *Int. J. Mol. Sci*. 10.3390/ijms21113828 (2020). 10.3390/ijms21113828 PMC7312552 32481589

[72] Subramanian A (2005). Gene set enrichment analysis: a knowledge-based approach for interpreting genome-wide expression profiles. Proc. Natl Acad. Sci. USA.

[73] Vajda S (2017). New additions to the ClusPro server motivated by CAPRI. Proteins.

[74] Kozakov D (2017). The ClusPro web server for protein-protein docking. Nat. Protoc..

[75] Kozakov D (2013). How good is automated protein docking?. Proteins.

