# Supplementary material for: RUNX3 inactivates oncogenic MYC through disruption of MYC/MAX complex and subsequent recruitment of GSK3β-FBXW7 cascade
Source: Commun Biol. 2023 Jul 3;6:689. doi: 10.1038/s42003-023-05037-0 (PMC10317990; doi:10.1038/s42003-023-05037-0)
Supplement: Supplementary file 1 — Supplementary Information [file 42003_2023_5037_MOESM1_ESM.pdf]

SUPPLEMENTAL INFORMATION

RUNX3 inactivates oncogenic MYC through disruption of MYC/MAX complex and subsequent recruitment of GSK3β-FBXW7 cascade

Vincent Oei, Linda Shyue Huey Chuang, Junichi Matsuo, Supriya Srivastava, Ming Teh and Yoshiaki Ito

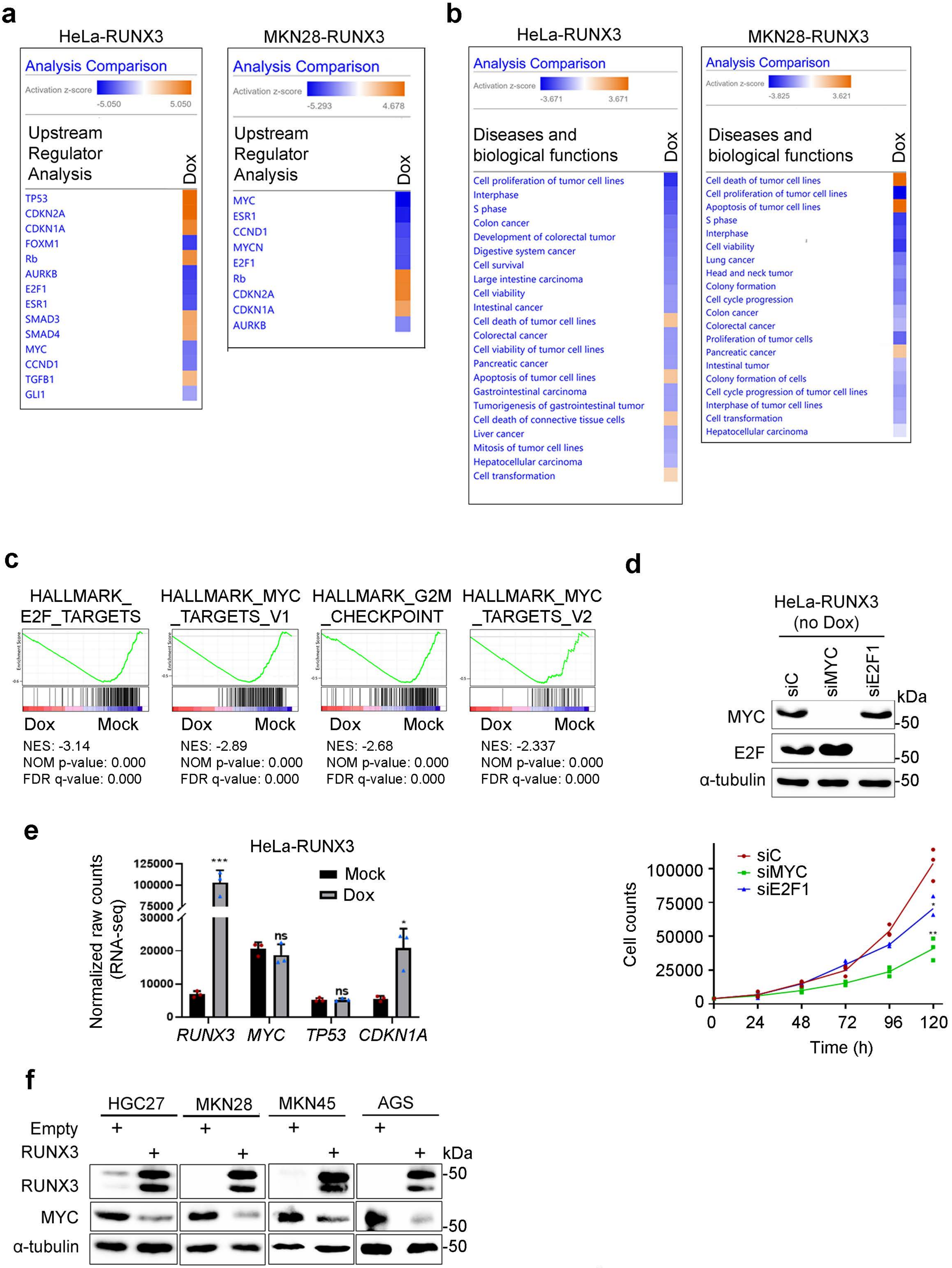

Supplementary Fig. 1

Supplementary Fig. 1: RUNX3 inhibits genes associated with cell proliferation.

(a) & (b) HeLa-RUNX3 was untreated (Mock) or treated with Dox (500 ng/ml) for 48 h, MKN28-RUNX3 was untreated (Mock) or treated with Dox (15 ng/ml) for 48 h. Experiments were performed 3 times and in 3 consecutive passages (n=3). Following RNAseq, DEGs were filtered using adjusted p-value of < 0.01 (for HeLa-RUNX3) and < 0.001 (for MKN28-RUNX3), $\log_2$ fold change > +/- 0.4. DEGs list (both up and down) were used as input for (a) upstream regulator analysis (IPA) and (b) Diseases and biological function analysis. Orange indicates activated (z score >= 2) Blue indicates Inactivated (z score =< 2).

(c) GSEA analysis of MKN-RUNX3 samples from (a).

(d) Top, Immunoblot of HeLa-RUNX3 treated with control siRNA (siC), siRNA targeting MYC (siMYC) and E2F1 (siE2F1) for 60 h. Bottom, Proliferation assay was performed by manual cell counting every 24 h up to 120 h (n=3). Data is presented as mean ± standard deviation. Asterisks indicate significant differences between groups at 120 h time point, $*p \le 0.05$, $**p \le 0.01$. Data is representative of two independent experiments.

(e) Normalized raw value of indicated mRNAs in HeLa-RUNX3 mock or Dox-treated as determined by DEseq2 analysis of RNA-seq data in (a). Data is from three independent experiments (n=3), presented as mean ± standard deviation. NS, not significant for $p > 0.05$; $*p \le 0.05$, $**p \le 0.01$, $***p \le 0.001$, $****p \le 0.0001$.

(f) Immunoblot of cells transfected with pEF-BOS (empty vector) and pEF-BOS-RUNX3 for 48 h. The data is representative of 3 independent experiments.

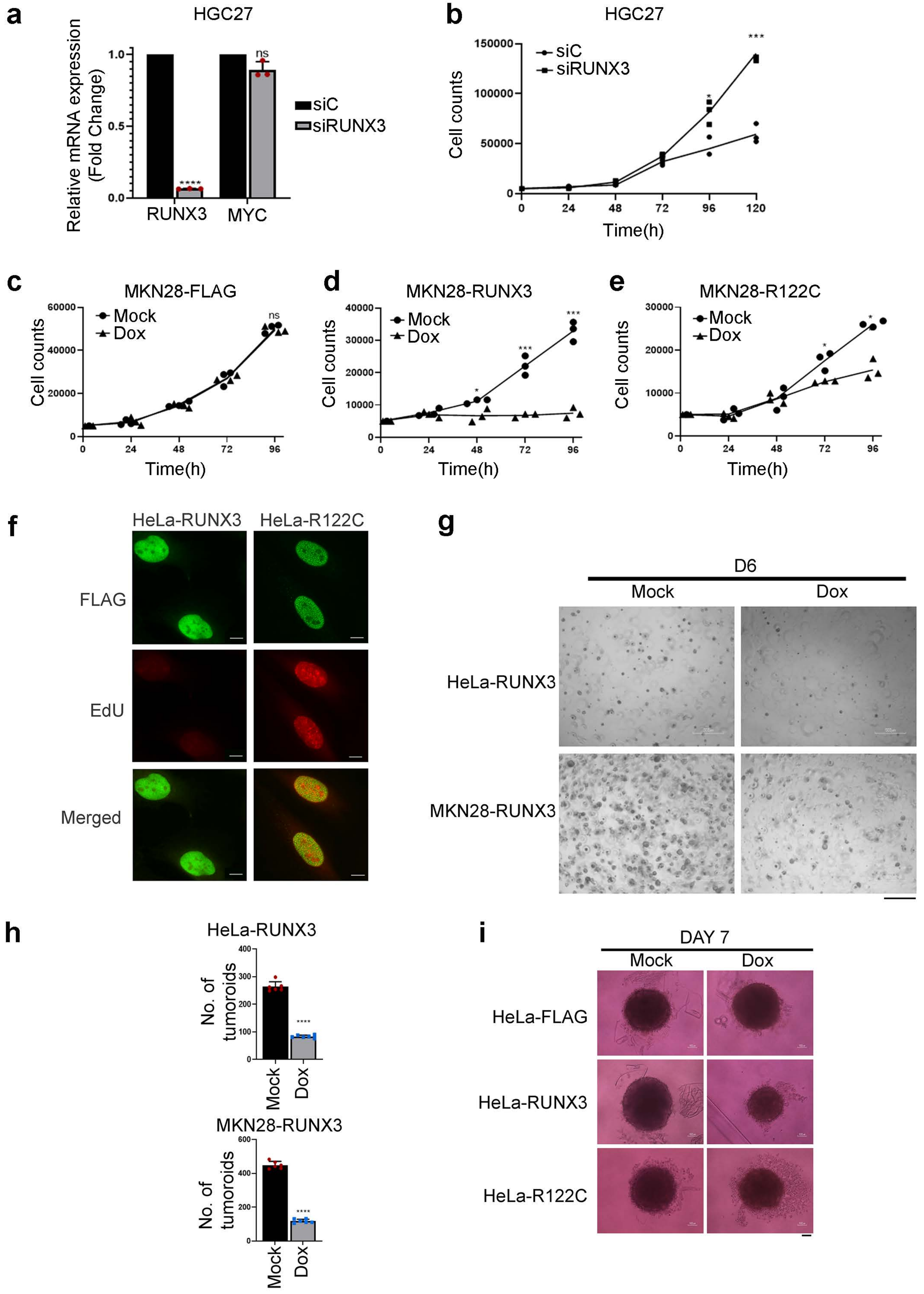

Supplementary Fig. 2

Supplementary Fig. 2: RUNX3 inhibits tumor proliferation and spheroid formation.

(a) Relative RNA expression of HGC27 cells treated with control siRNA (siC), siRNA targeting RUNX3 (siRUNX3) for 60 h. RT-qPCR was performed. The data are presented as means ± standard deviation (n = 3) relative to the SiControl group and shown in bar graphs. NS, not significant for $p > 0.05$, ****$p \leq 0.0001$. The data is representative of 3 independent experiments.

(b) Cell proliferation assay of HGC27 cells in (a). Manual cell counting was performed every 24 h up to 120 h (n=3). Data is presented as mean ± standard deviation. Asterisks indicate significant differences between groups at 96 h and 120 h time point, *$p \leq 0.05$, **$p \leq 0.01$, ***$p \leq 0.001$. The data is representative of 3 independent experiments.

(c) – (e) Proliferation assay of MKN28-FLAG, MKN28-RUNX3, MKN28-R122C without (mock) and with Dox treatment (10 ng/ml). Cells were plated in triplicates (n=3) and manually counted every 24 hours. Data is presented as mean ± standard deviation. Asterisks indicate significant differences between groups at indicated time point. NS, not significant for $p > 0.05$, *$p \leq 0.05$, **$p \leq 0.01$, ***$p \leq 0.001$. The data is representative of 3 independent experiments.

(f) EdU incorporation proliferation assay of HeLa-RUNX3 and HeLa-R122C. Fluorescence staining shows FLAG-tagged RUNX3 and R122C (green) and newly replicated DNA with EdU incorporation (red). Scale bar, 10 µm. The data is representative of 2 independent experiments.

(g) Representative images of tumoroids formed by HeLa-RUNX3 and MKN28-RUNX3 6 days (D6) in the presence or absence of Dox. Scale bar, 500 µm. The data is representative of 2 independent experiments.

(h) The total number of tumoroids in (g) were plotted (6 replicates per sample, n=6) by GraphPadPrism. Data is presented as mean ± standard deviation, ****$p \leq 0.0001$.

(i) Representative images of HeLa-FLAG, HeLa-RUNX3 and HeLa-R122C tumor spheroids 7 days after plating on low cell attachment surface plates. Dox (500 ng/ml) was added where indicated. Scale bar, 100 µm. The data is representative of 2 independent experiments.

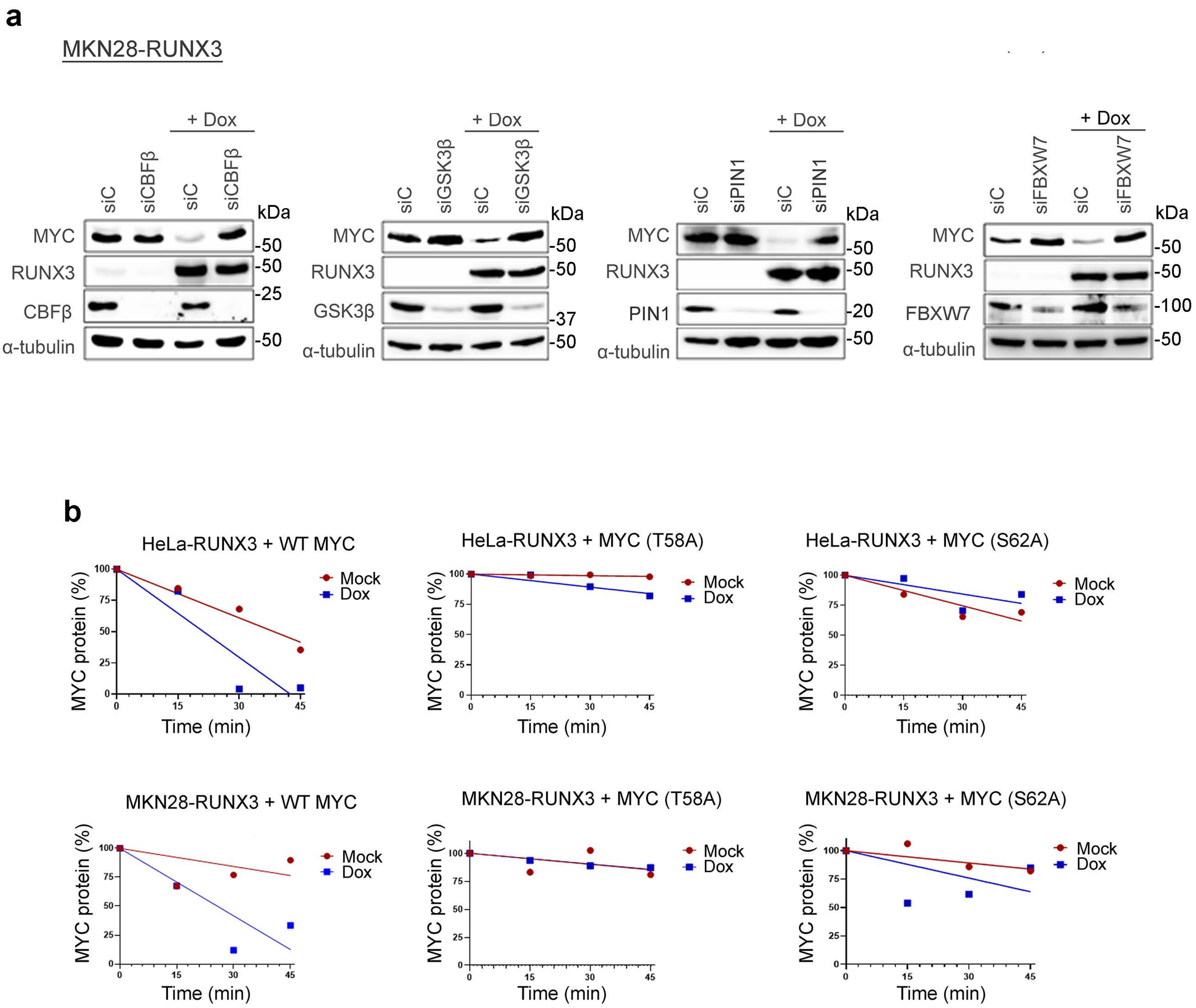

Supplementary Fig. 3: RUNX3 destabilizes MYC via the GSK3β-FBXW7 pathway in a cell type independent manner.

(a) Immunoblot of MKN28-RUNX3 cells treated with control siRNA (siC), and siRNA targeting CBFβ (siCBFβ), GSK3β (siGSK3β), PIN1 (siPIN1), and FBXW7 (siFBXW7). After 12 h, 15 ng/ml Dox was added where indicated. Cells were harvested 48 h after Dox addition. The data is representative of 3 independent experiments.

(b) Densitometric quantification of band intensity from Fig. 2e using ImageJ. Graph and linear regression line were plotted using GraphPad Prism. The data is representative of 2 independent experiments.

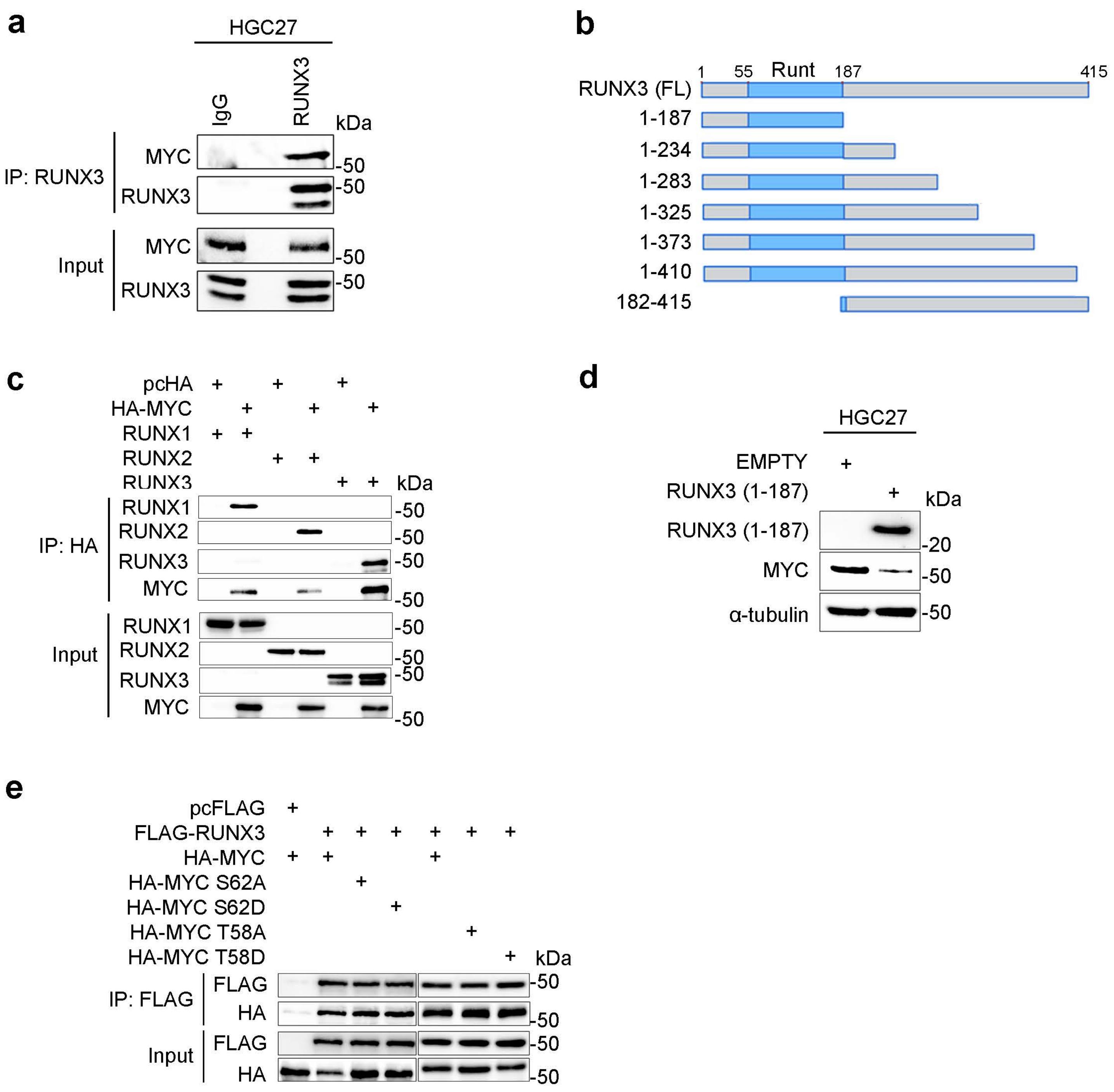

Supplementary Fig. 4: RUNX proteins interact with MYC.

(a) Immunoprecipitation of HGC27 cell lysates using IgG control and anti-RUNX3 antibodies to detect endogenous RUNX3/MYC interaction. Representative of 3 independent experiments.

(b) FLAG-tagged truncation constructs of RUNX3. FL, full-length. Numbers indicate amino acid residues. Created with Biorender.com.

(c) Immunoprecipitation and immunoblot of HEK293T cells transfected with indicated combinations of pEF-BOS-RUNX1, -RUNX2 and -RUNX3 and HA-tagged-MYC for 24 h. Representative of 3 independent experiments.

(d) Immunoblot of HGC27 cells transfected with FLAG-tagged RUNX3 (aa 1-187) or control vector pcFLAG (empty) for 24 h. α-tubulin is the loading control. Representative of 3 independent experiments.

(e) Immunoprecipitation and immunoblot of HEK293T cells transfected with the indicated plasmids. Anti-FLAG and anti-HA antibodies was used to detect RUNX3 and MYC phosphorylation mutants respectively. Representative of 3 independent experiments.

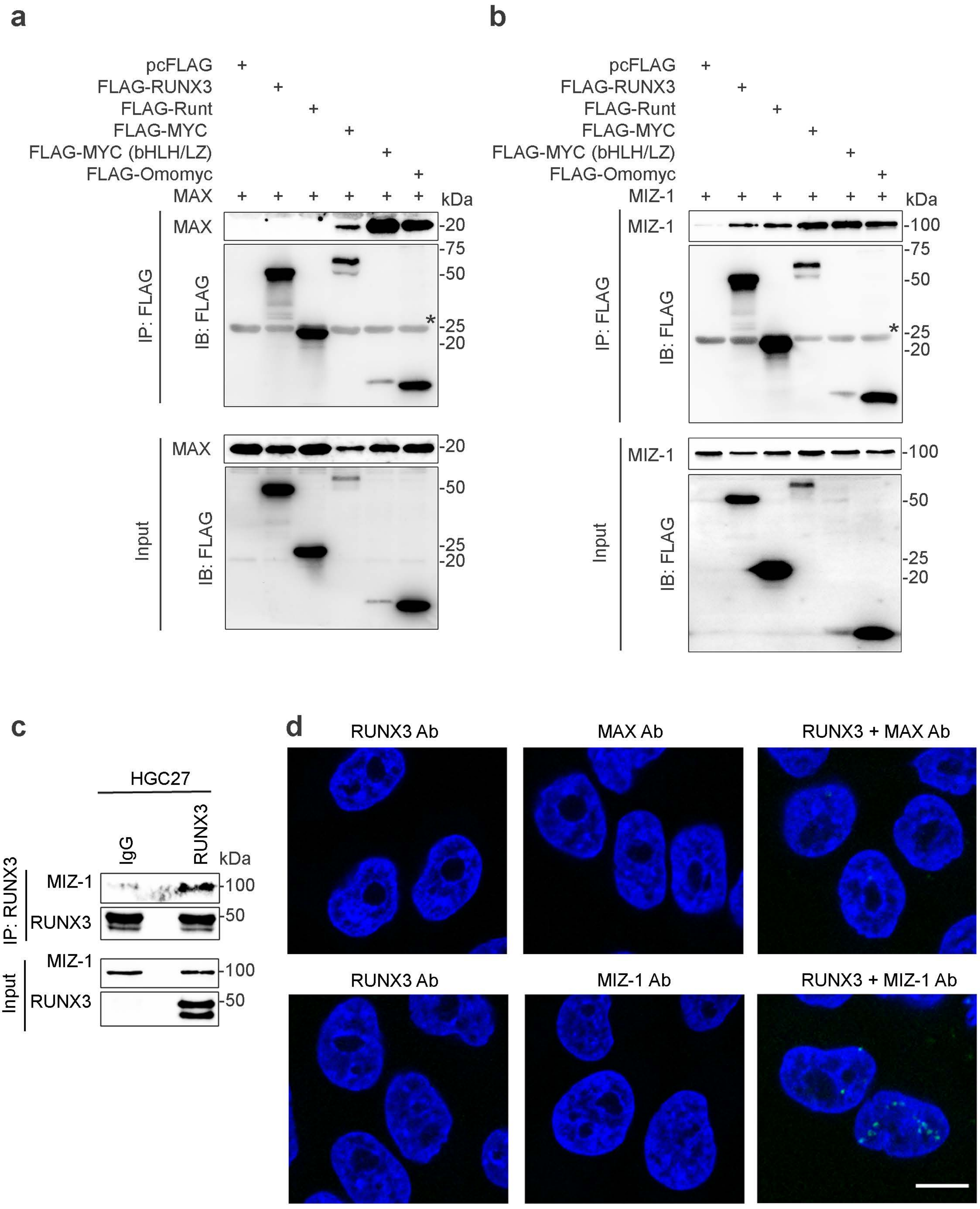

Supplementary Fig. 5

Supplementary Fig. 5: RUNX3 interacts with MIZ-1, not MAX.

(a) Immunoprecipitation (IP) and immunoblot (IB) of HEK293T cells were transfected with MAX expression vector and FLAG-tagged RUNX3 constructs, FLAG-tagged MYC constructs and FLAG-Omomyc for 24 h. FLAG-Runt refers to FLAG-RUNX3 (aa 1-187). * indicates the FLAG antibodies used for IP. Representative of 2 independent experiments.

(b) As in (a) except that MIZ-1 was used instead of MAX. Representative of 2 independent experiments.

(c) HGC27 cell lysate was used for co-immunoprecipitation of endogenous proteins RUNX3 and MIZ1. Representative of 2 independent experiments.

(d) Proximity ligation assay to visualize endogenous protein MIZ-1 and RUNX3 interaction in HGC27 cell nuclei. The absence of signals in presence of MAX and RUNX3 antibodies (Ab) served as negative control for specificity of MIZ-1/RUNX3 complex detection. Representative of 2 independent experiments.

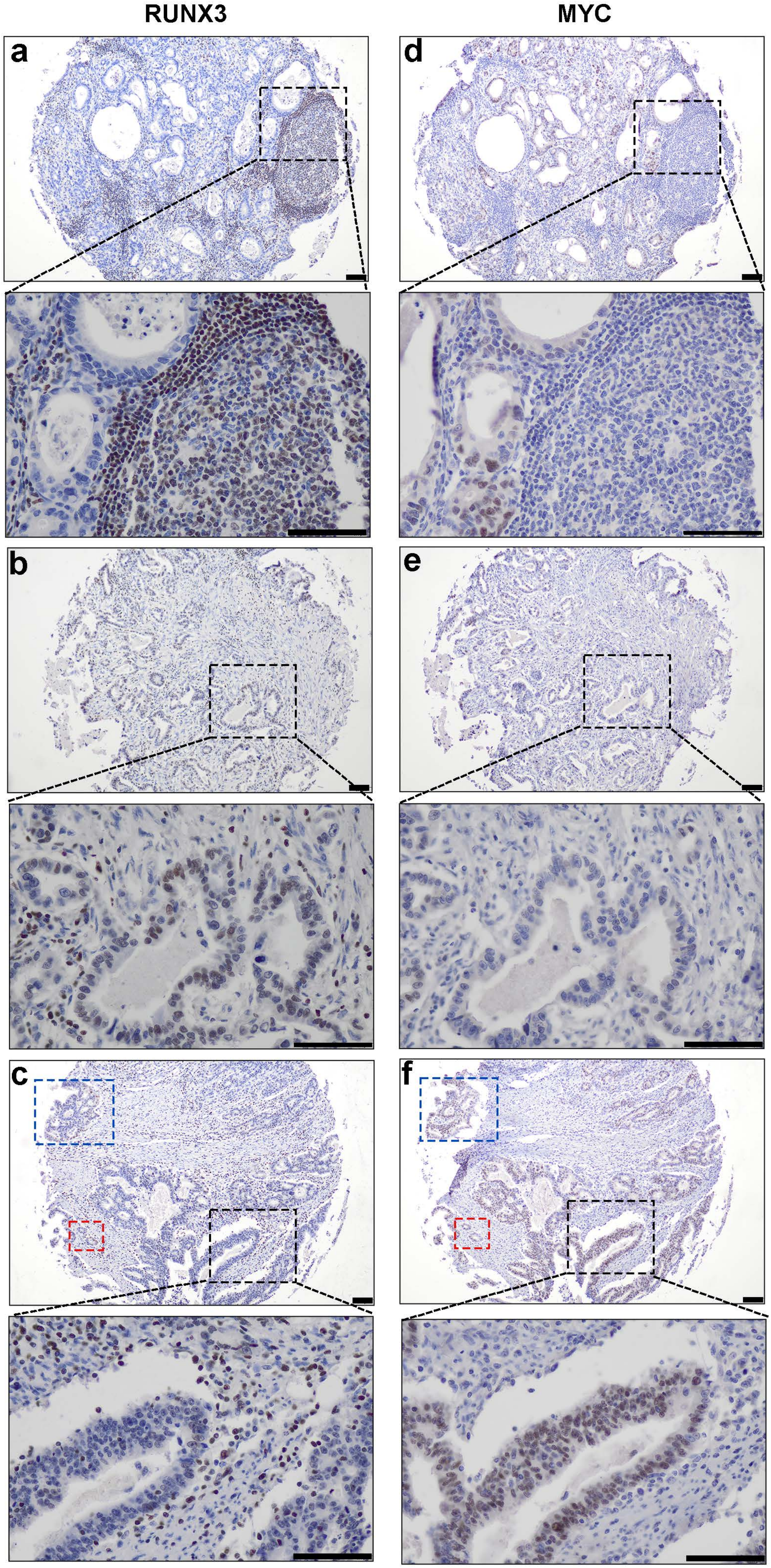

Supplementary Fig. 6

Supplementary Fig. 6: Inverse correlation of RUNX3 and MYC protein levels in human gastric tumor microarrays

(a), (b) and (c), Immunohistochemistry (IHC) showing RUNX3 protein expression in gastric tumor cells. (d), (e) and (f), IHC showing MYC protein expression in the corresponding tumor cores. Representative photographs at 100x and 400x magnifications are shown. Boxed regions in (a) and (d) indicate lymphoid aggregates. Boxed regions in (b) and (e) indicate moderate RUNX3- and low MYC-expressing tumor cells. Boxed regions in (c) and (f) indicate low RUNX3- and high MYC-expressing tumor cells. Red and blue boxed regions will be referred to in Figure S7. Scale bar, 200 μm.

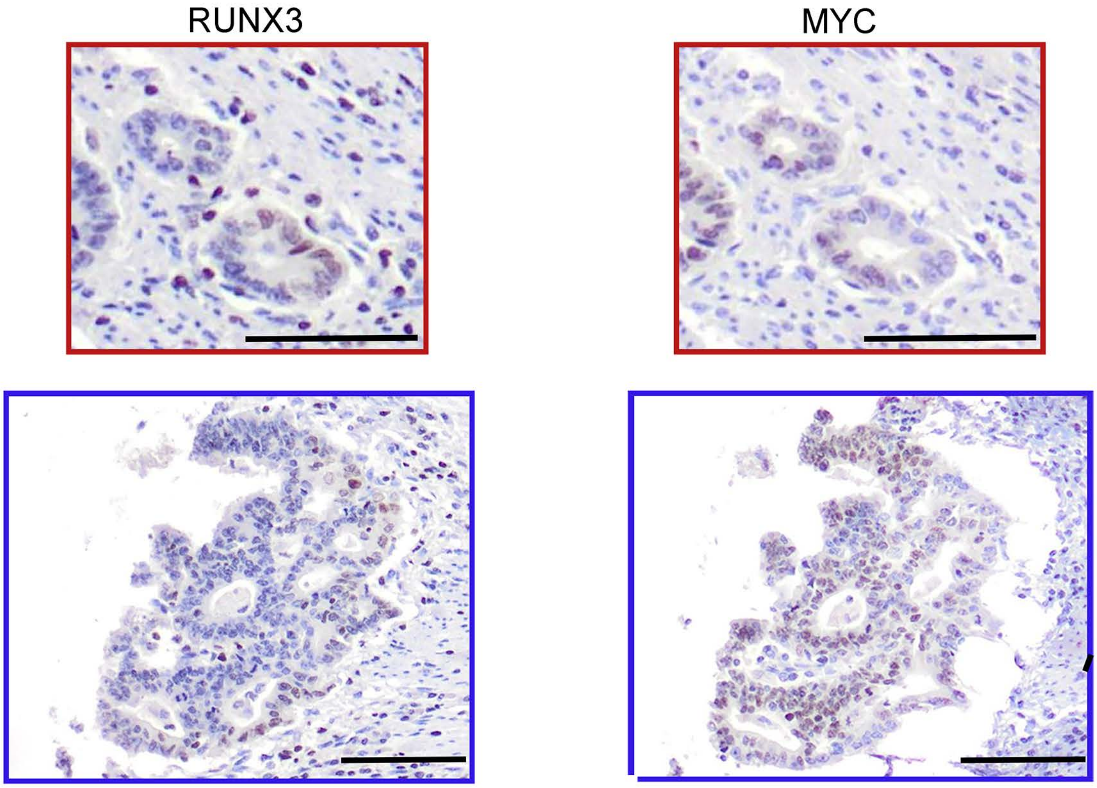

Supplementary Fig. 7: Inverse correlation of RUNX3 and MYC within a malignant gastric tumor tissue

Higher magnification images of red and blue boxed regions from tumor core in Supplementary Fig.S6c and S6f showing RUNX3 and MYC protein expression, respectively. Scale bar, 200 µm.

Supplementary Fig. 8: Scans of immunoblots in Figures 1c and 1d

Figure 1c

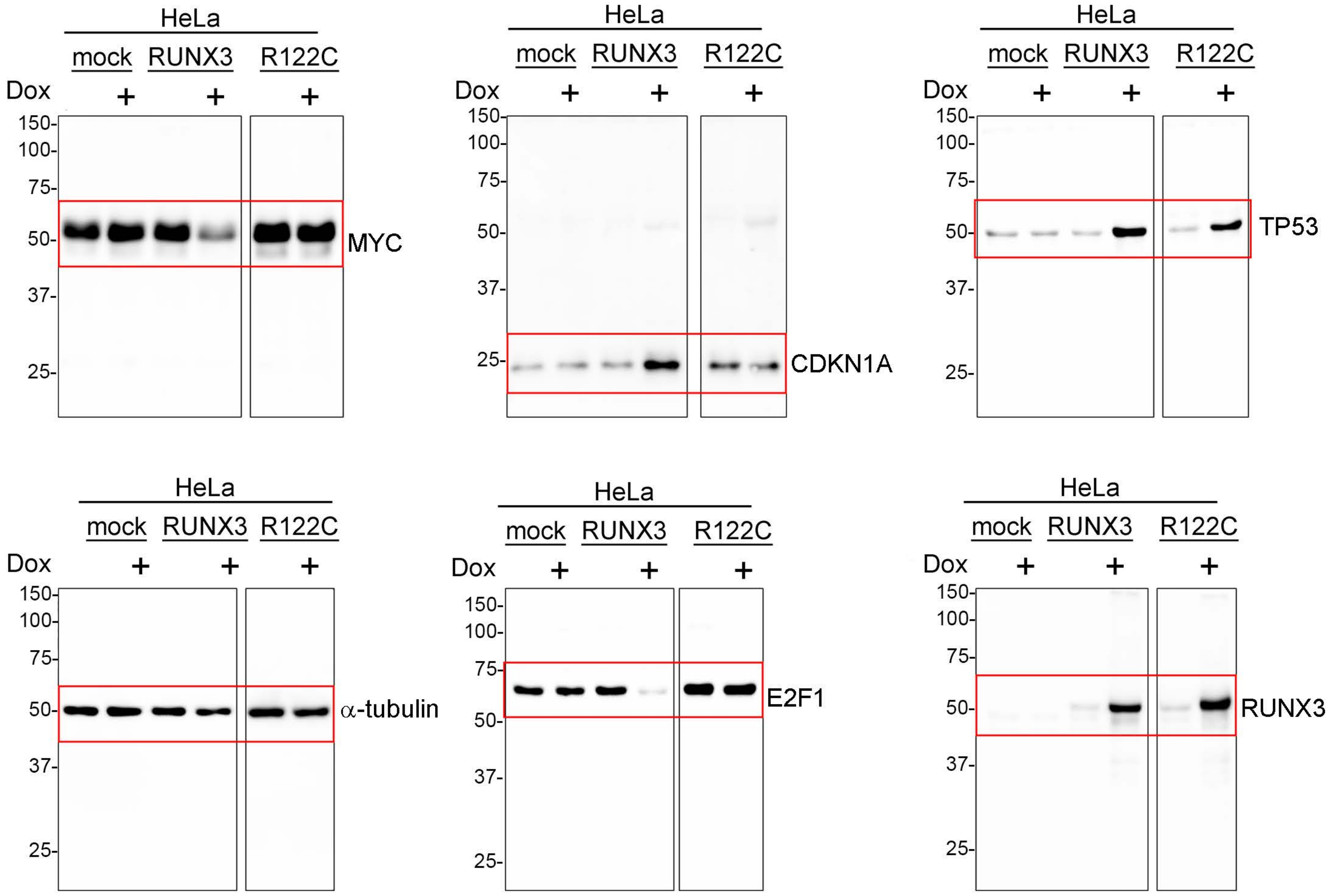

Figure 1d

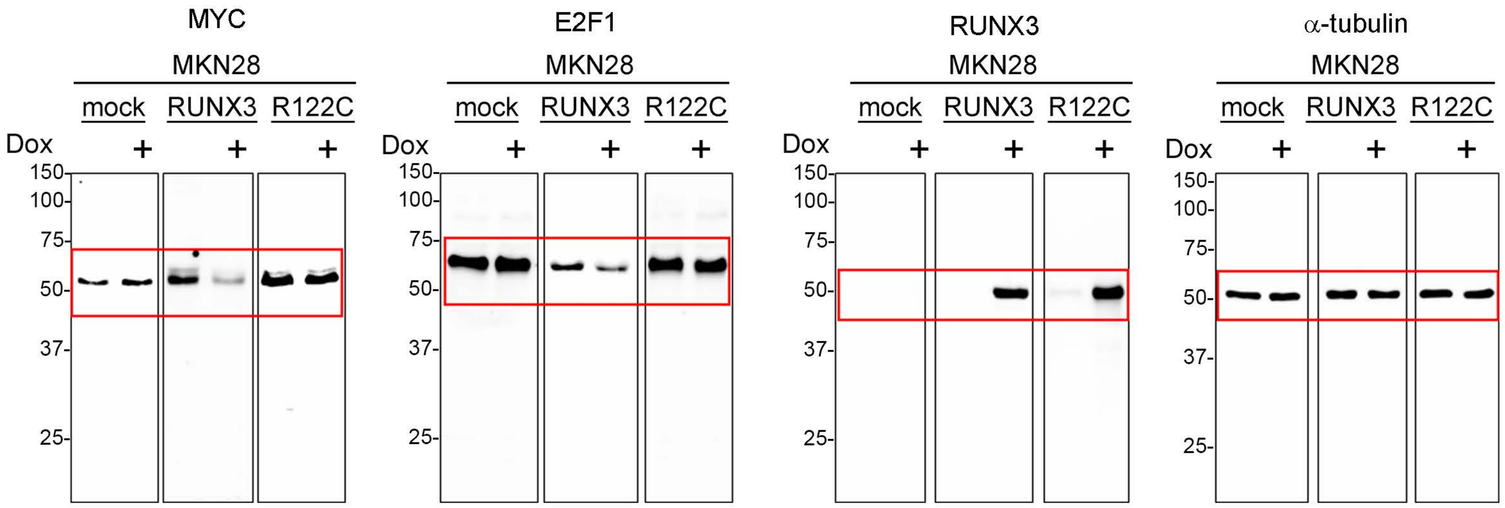

Supplementary Fig. 9: Scans of immunoblots from Figures 1e, 1g and 1i

Figure 1e

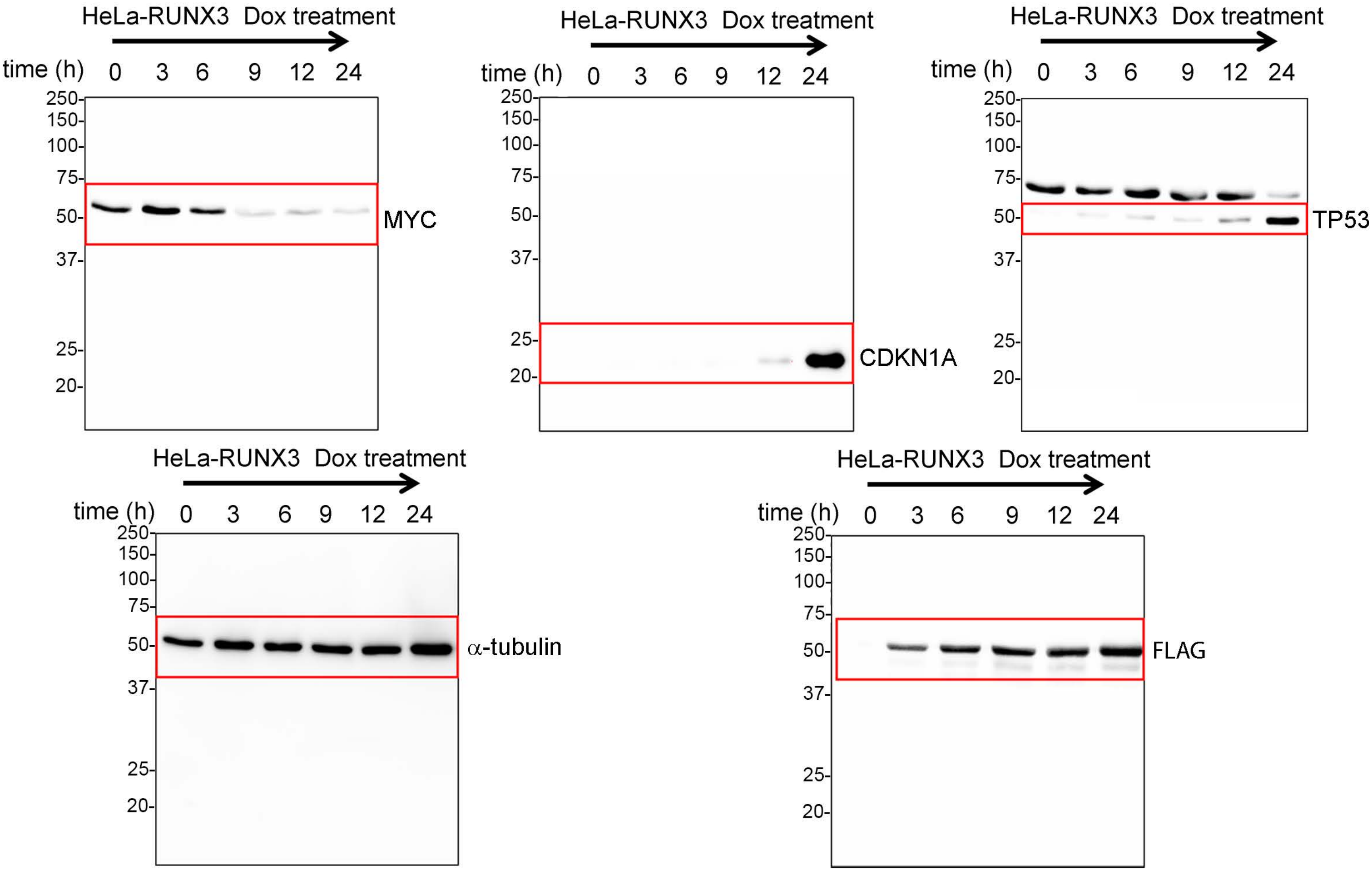

Figure 1g

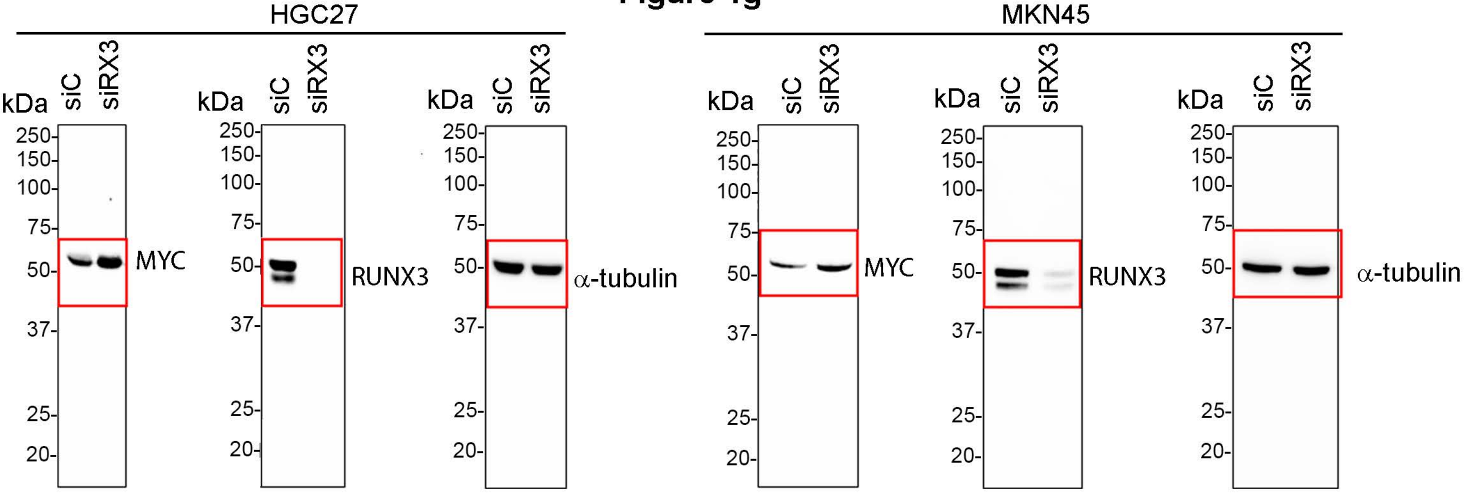

Figure 1i

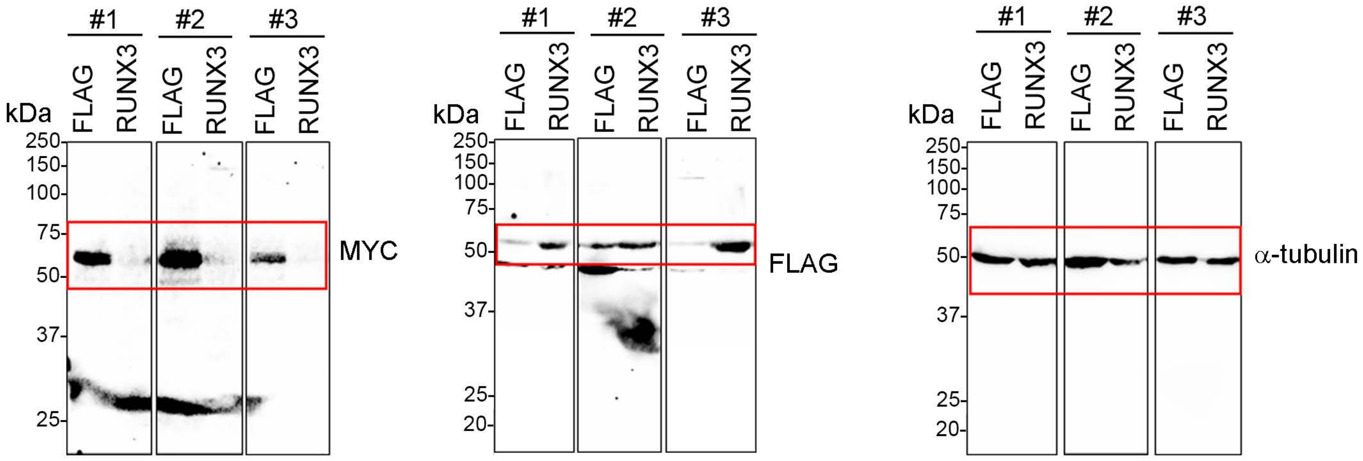

Supplementary Fig. 10: Scans of immunoblots in Figures 2a and 2b

Figure 2a

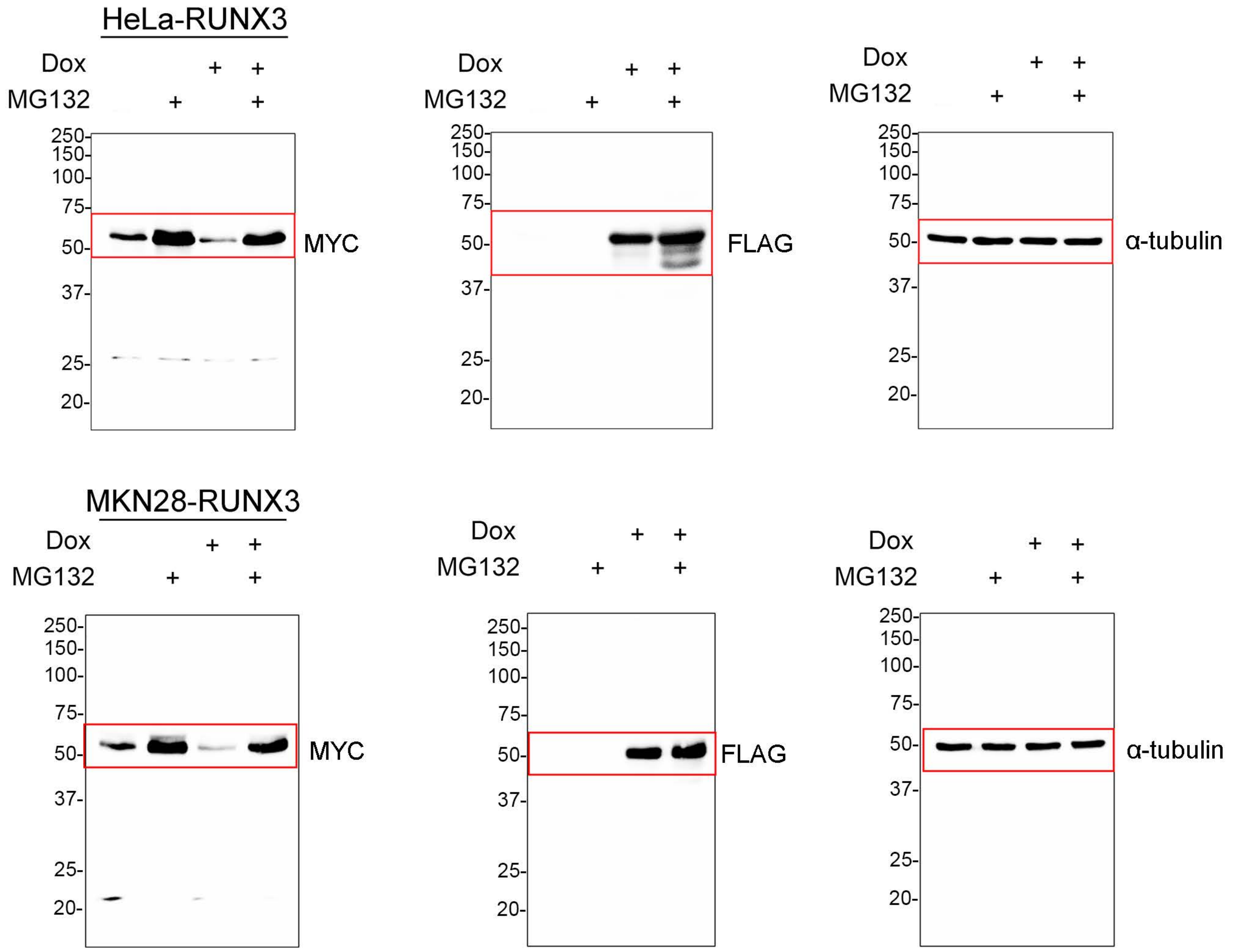

Figure 2b

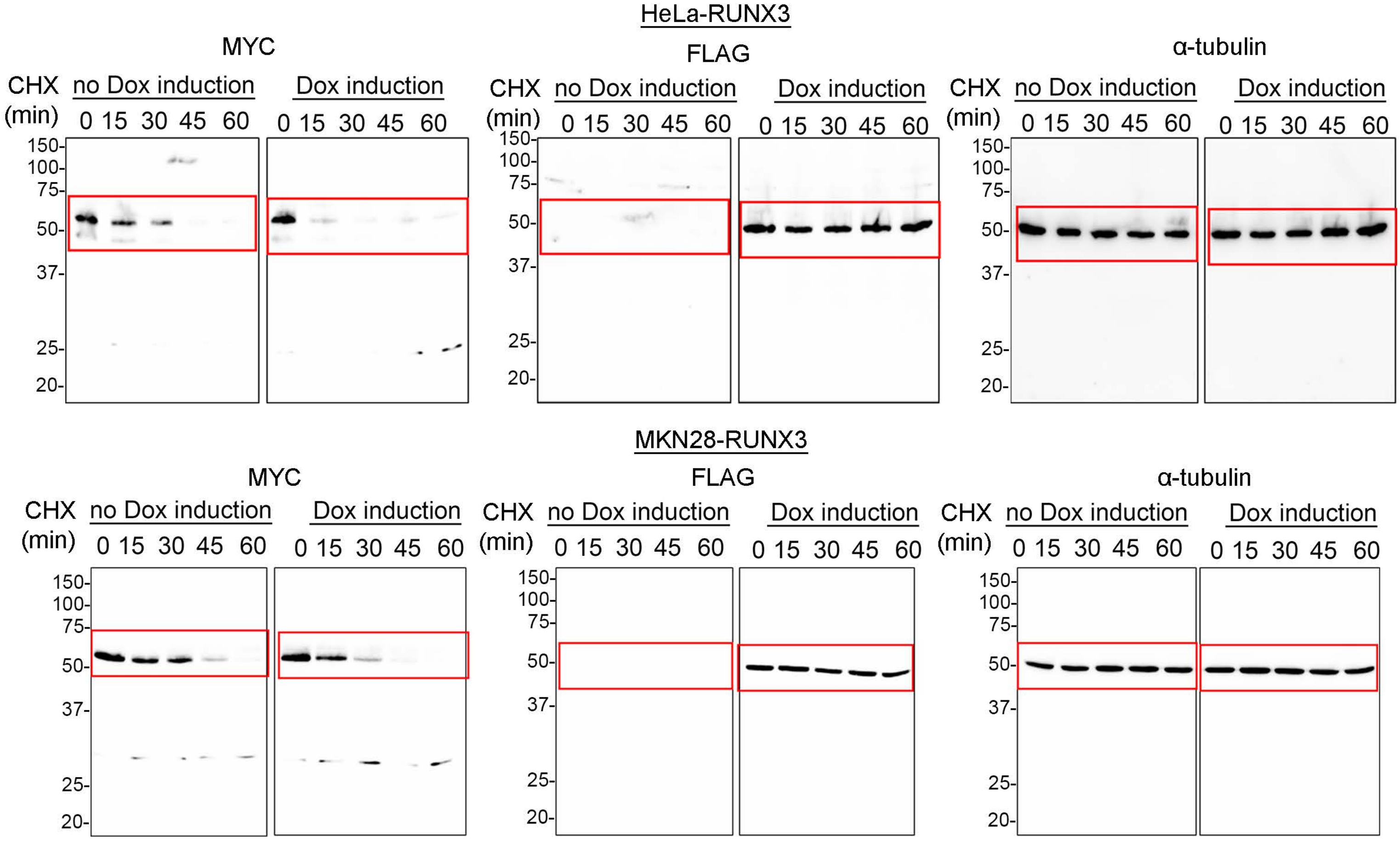

Supplementary Fig. 11: Scans of immunoblots in Figure 2d

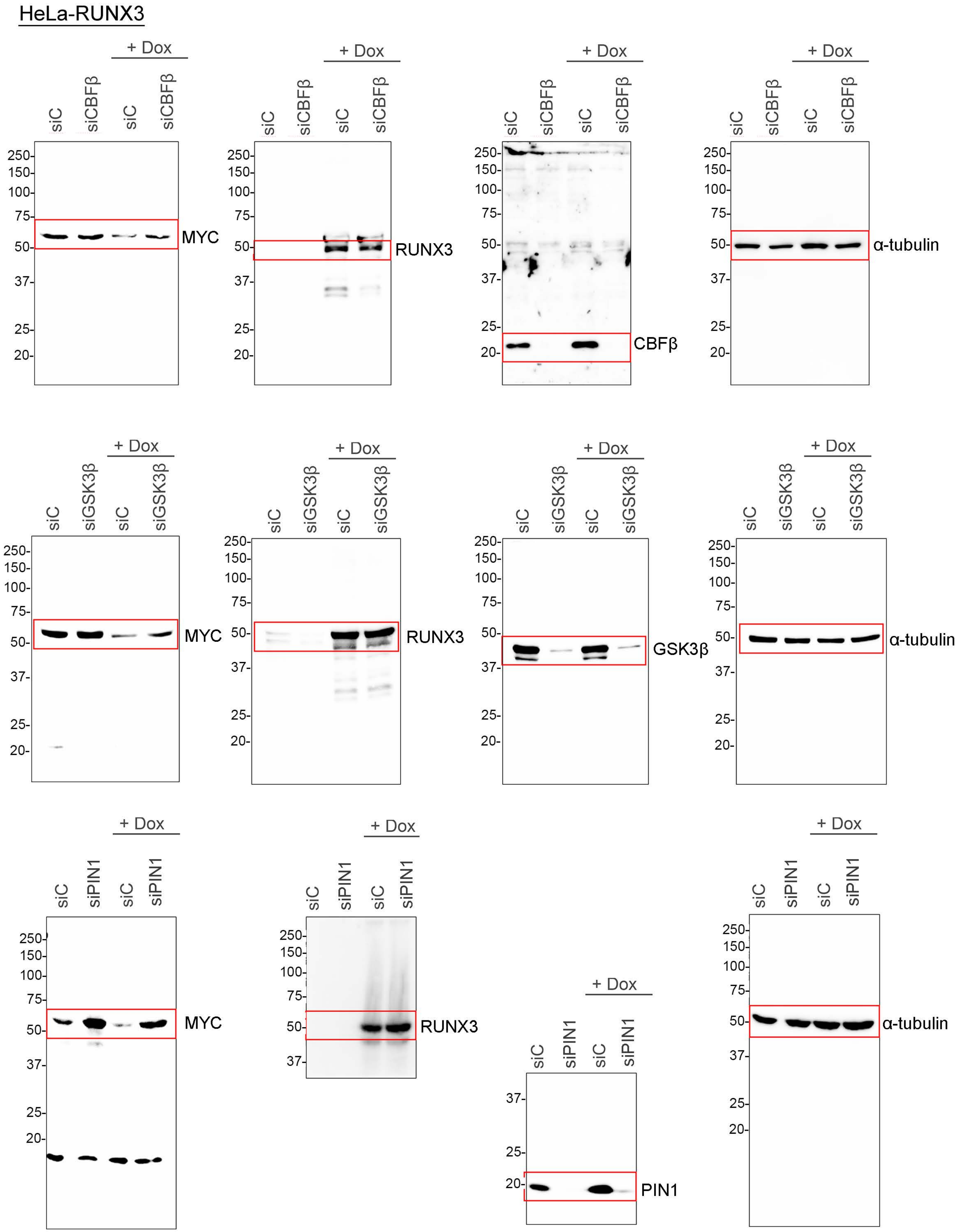

Supplementary Fig. 12: Scans of immunoblots in Figures 2d and 2e

Figure 2d

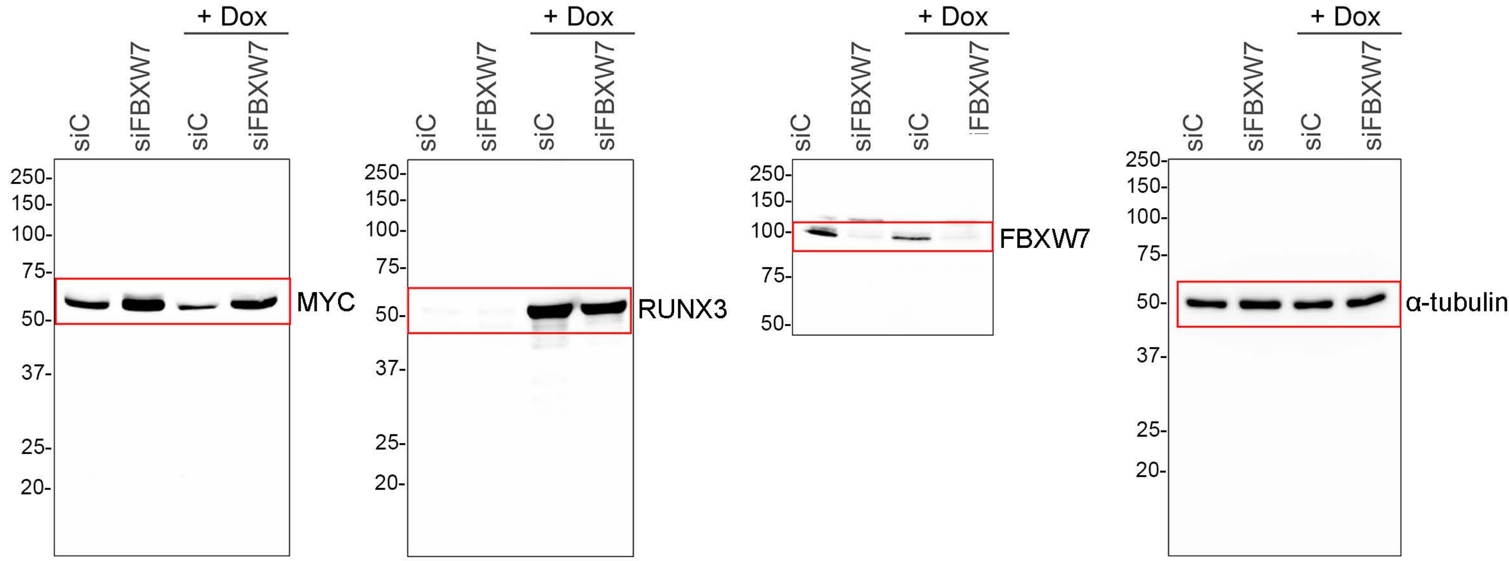

Figure 2e

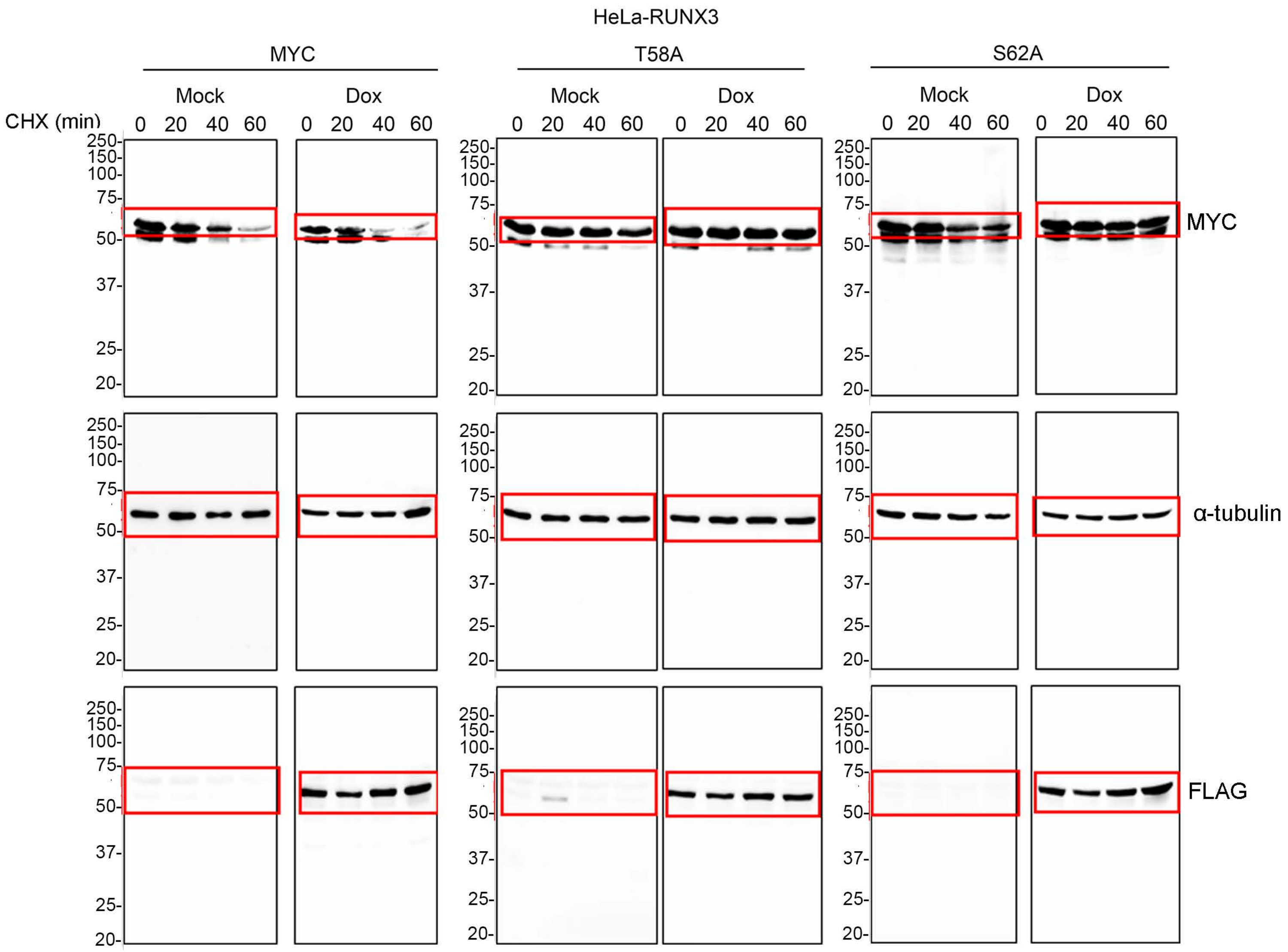

Supplementary Fig. 13: Scans of immunoblots in Figure 2e

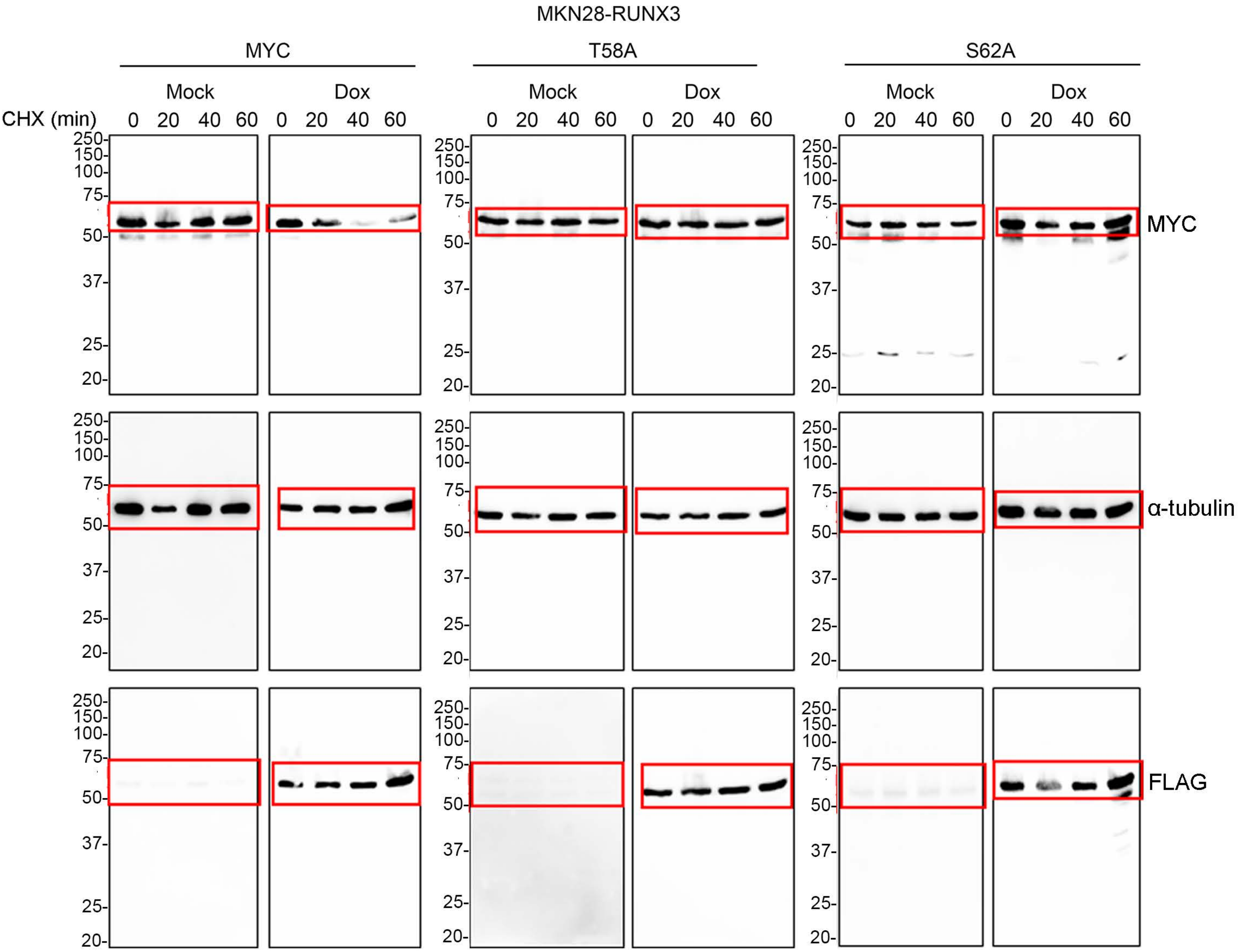

Supplementary Fig. 14: Scans of immunoblots in Figures 3a and 3b

Figure 3a

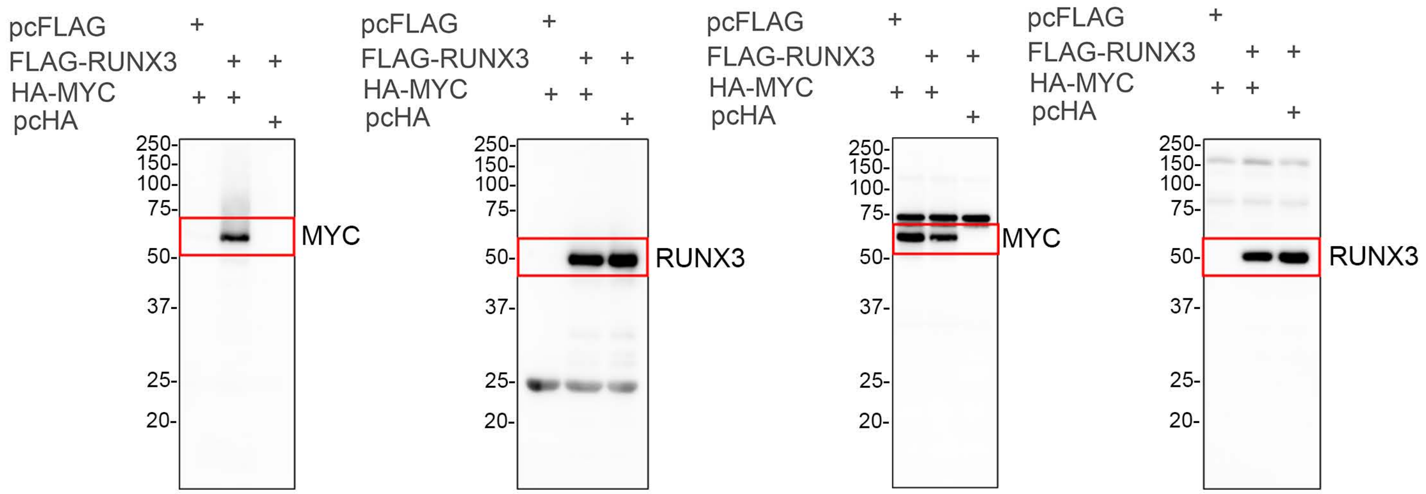

Figure 3b

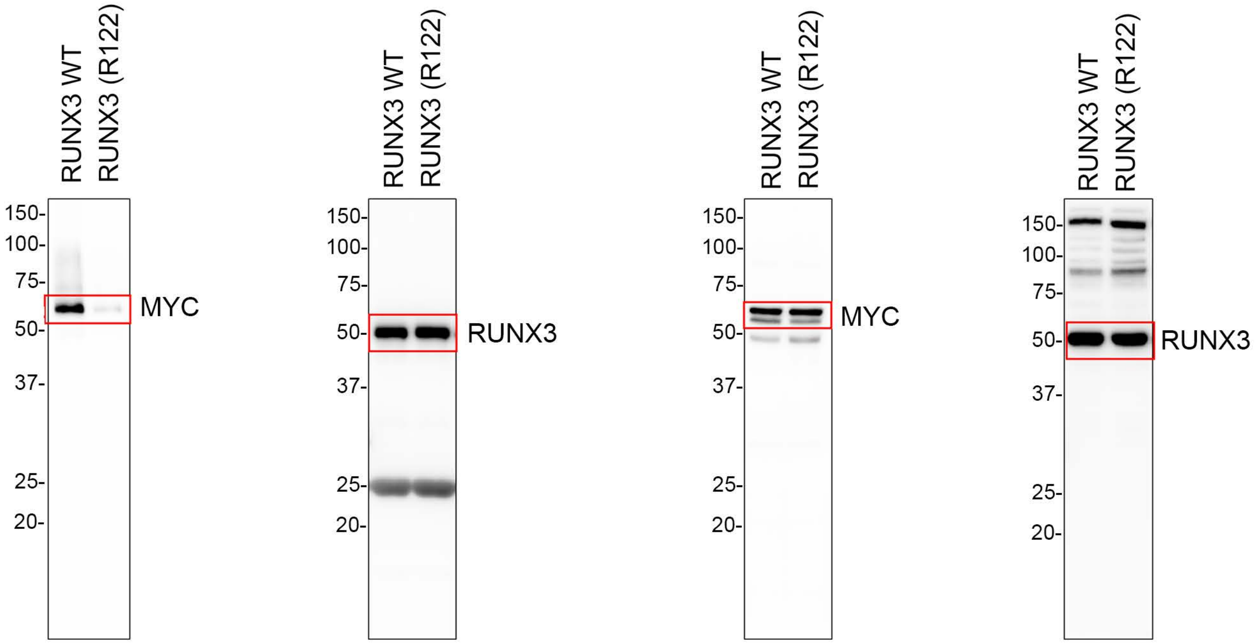

Supplementary Fig. 15: Scans of immunoblots in Figures 3e and 3f

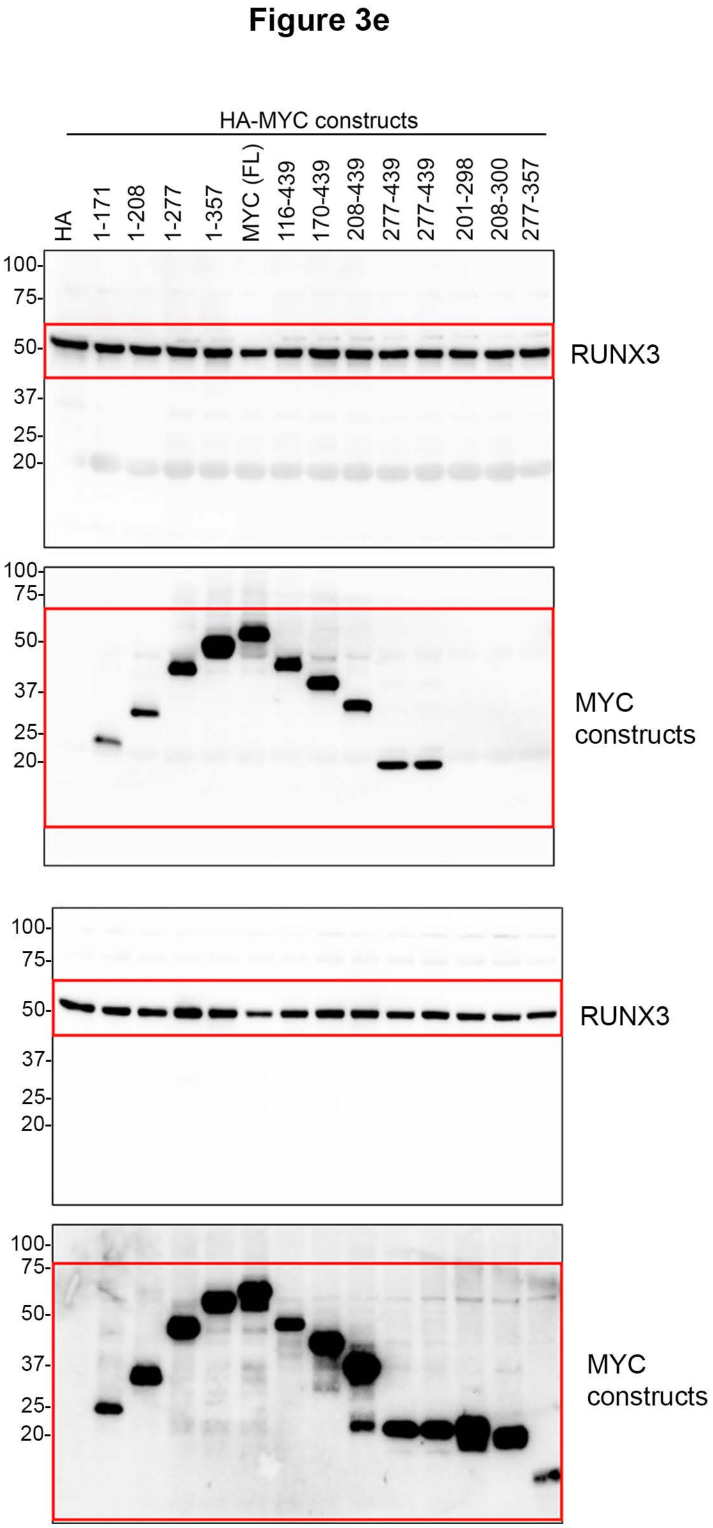

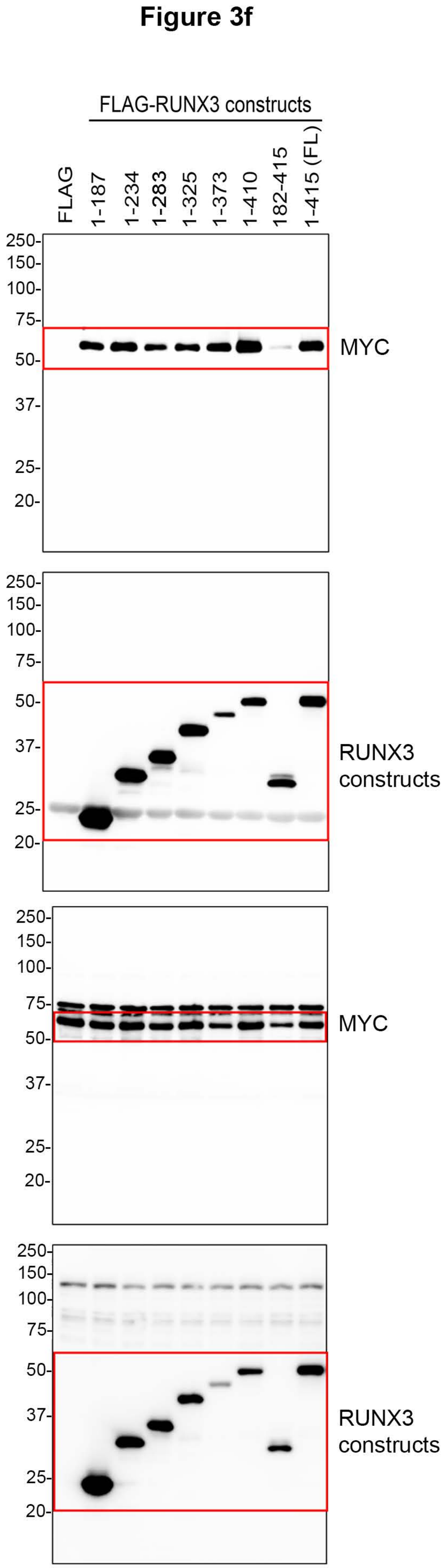

Supplementary Fig. 16: Scans of immunoblots and gel in Figures 3g and 4a

Figure 3g

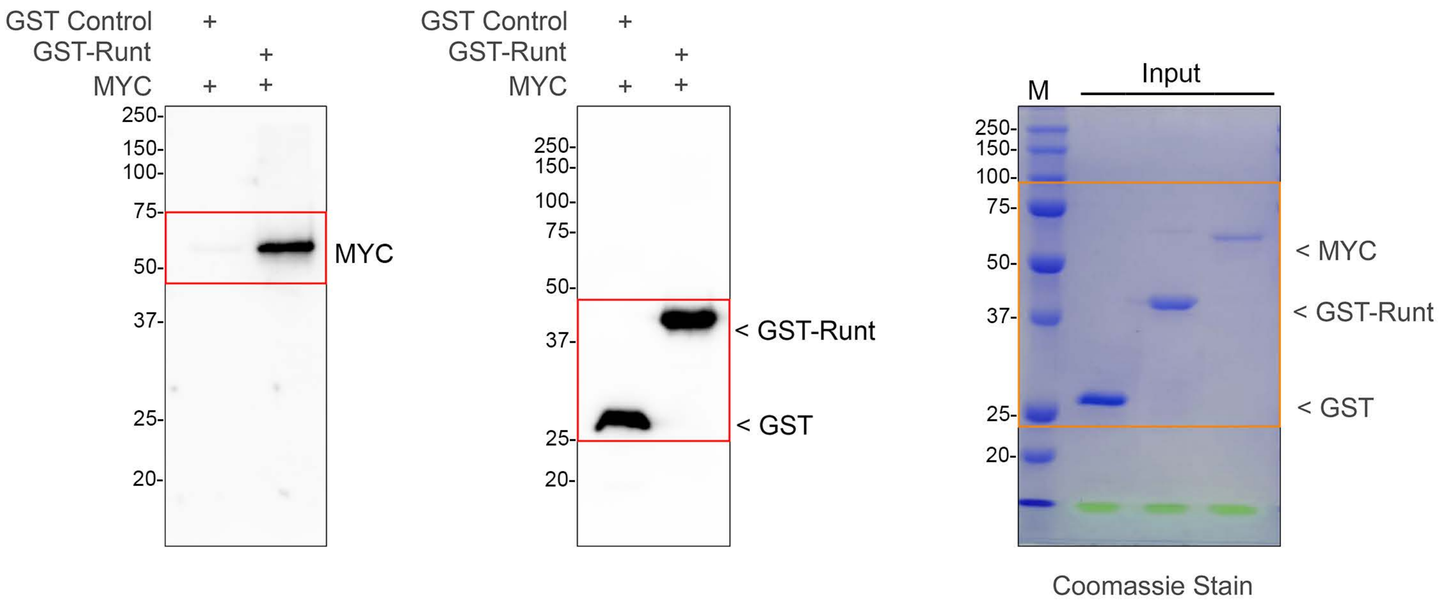

Figure 4a

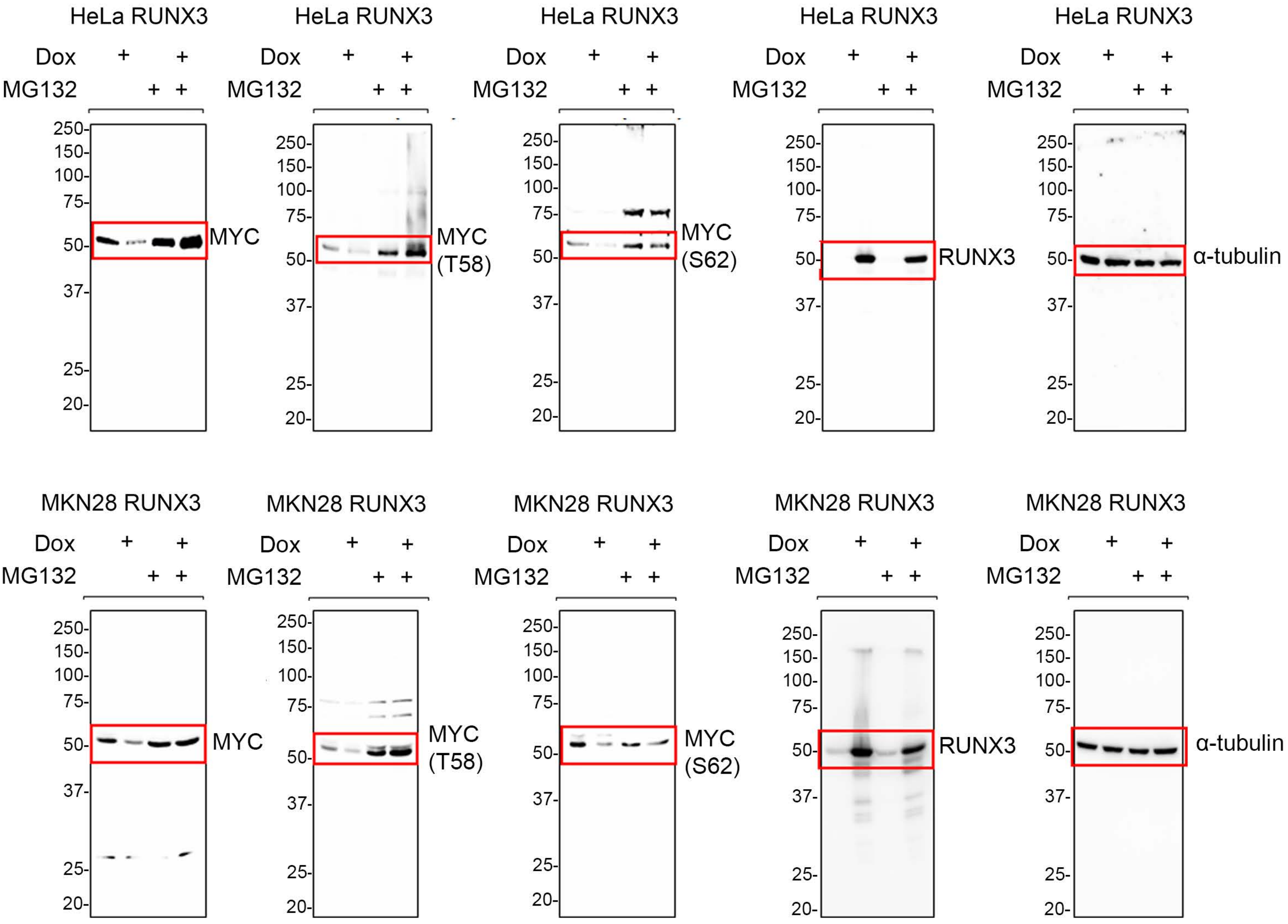

Supplementary Fig. 17: Scans of immunoblots in Figures 4b and 4d

Figure 4b

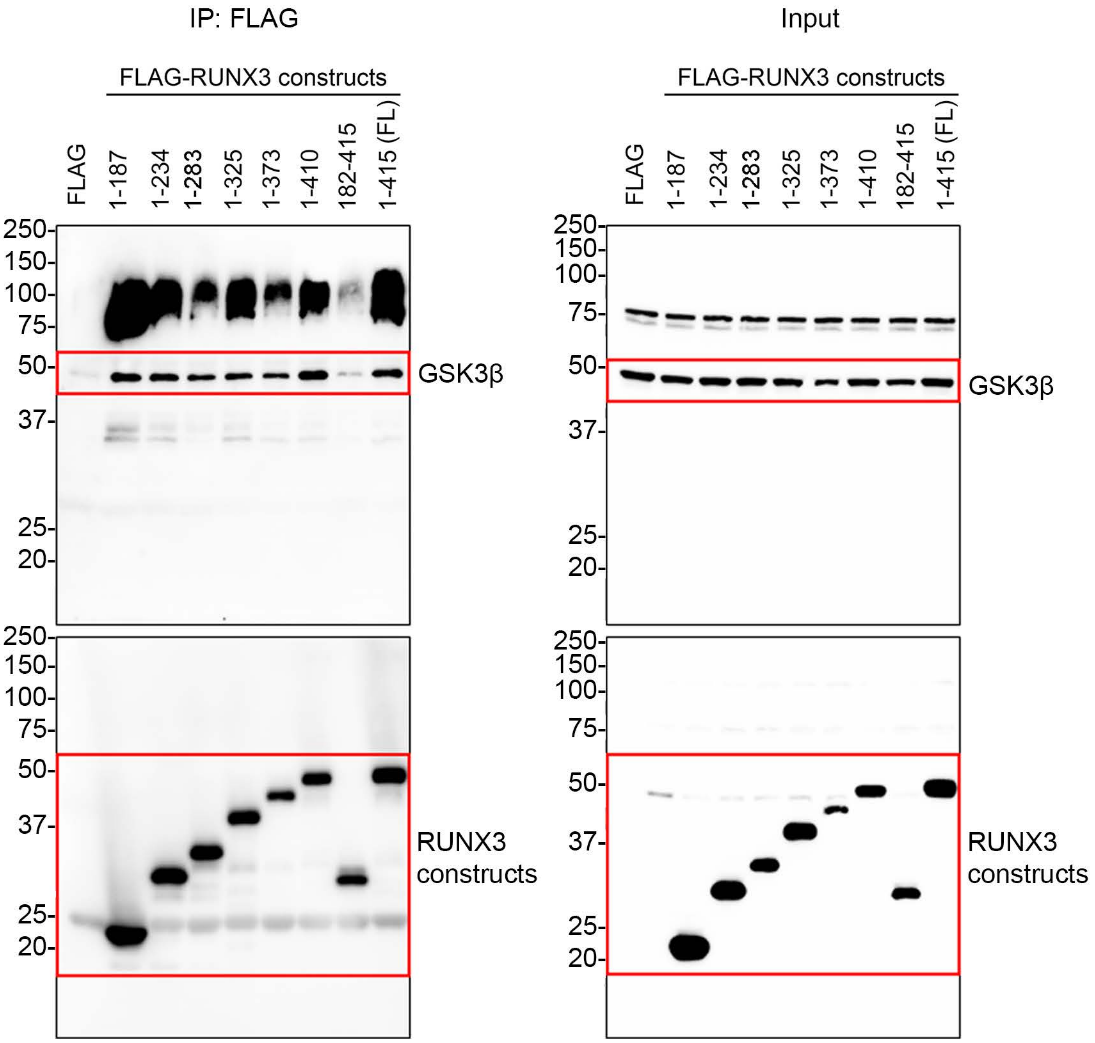

Figure 4d

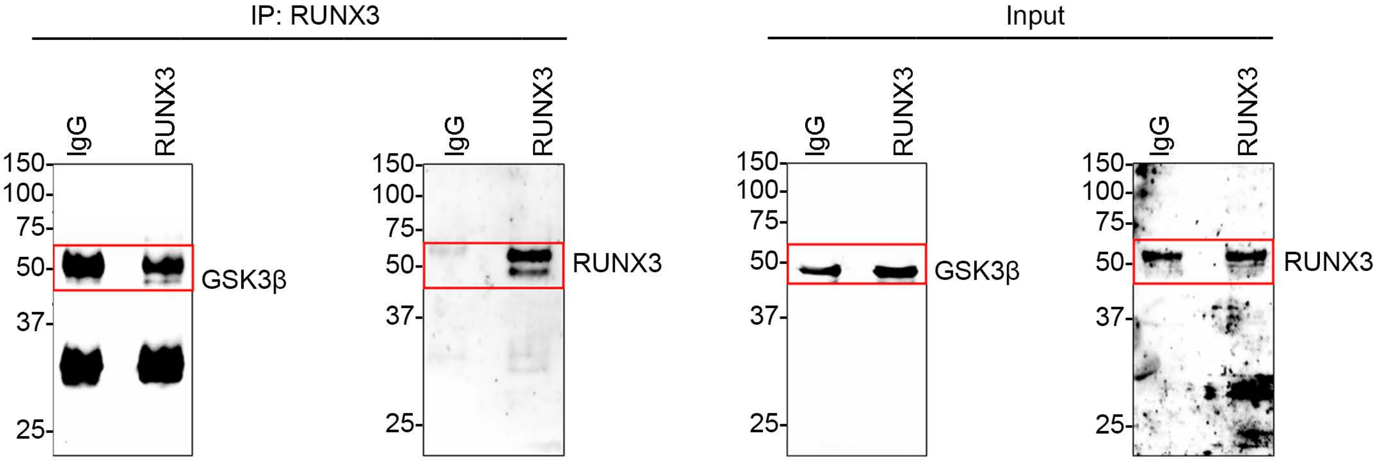

Supplementary Fig.18: Scans of immunoblots in Figures 4e and 4f

Figure 4e

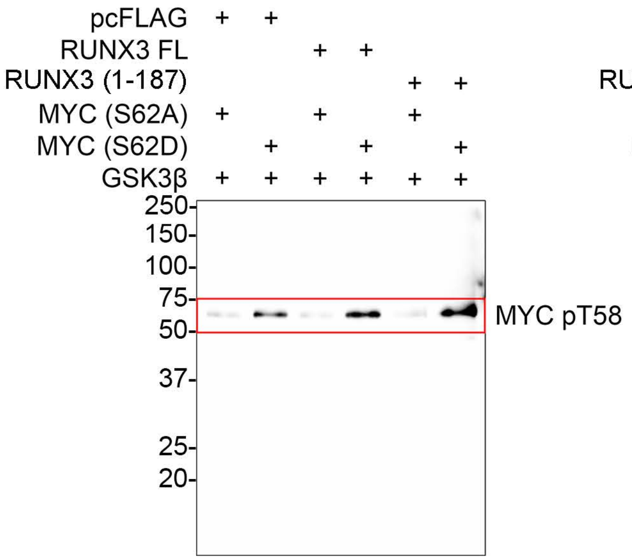

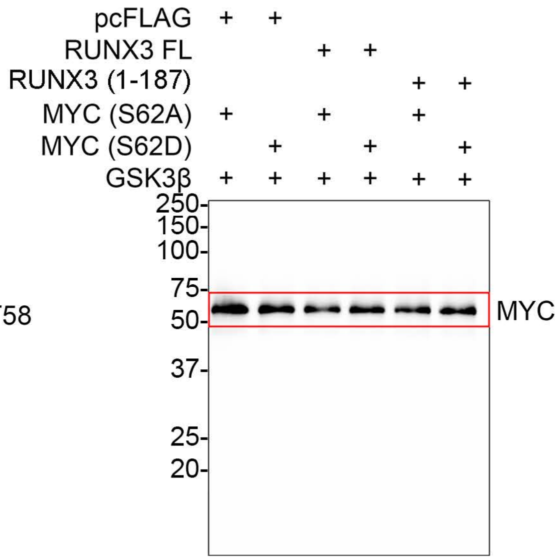

Figure 4f

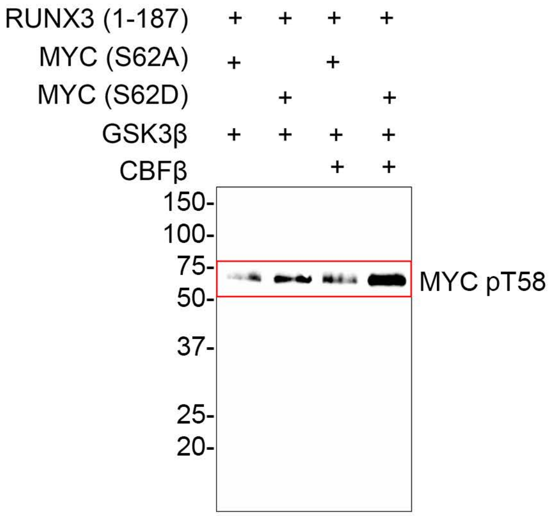

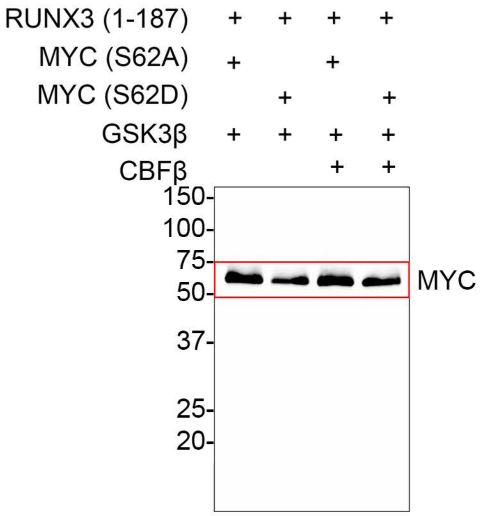

Supplementary Fig.19: Scans of immunoblots from Figure 4g

Figure 4g

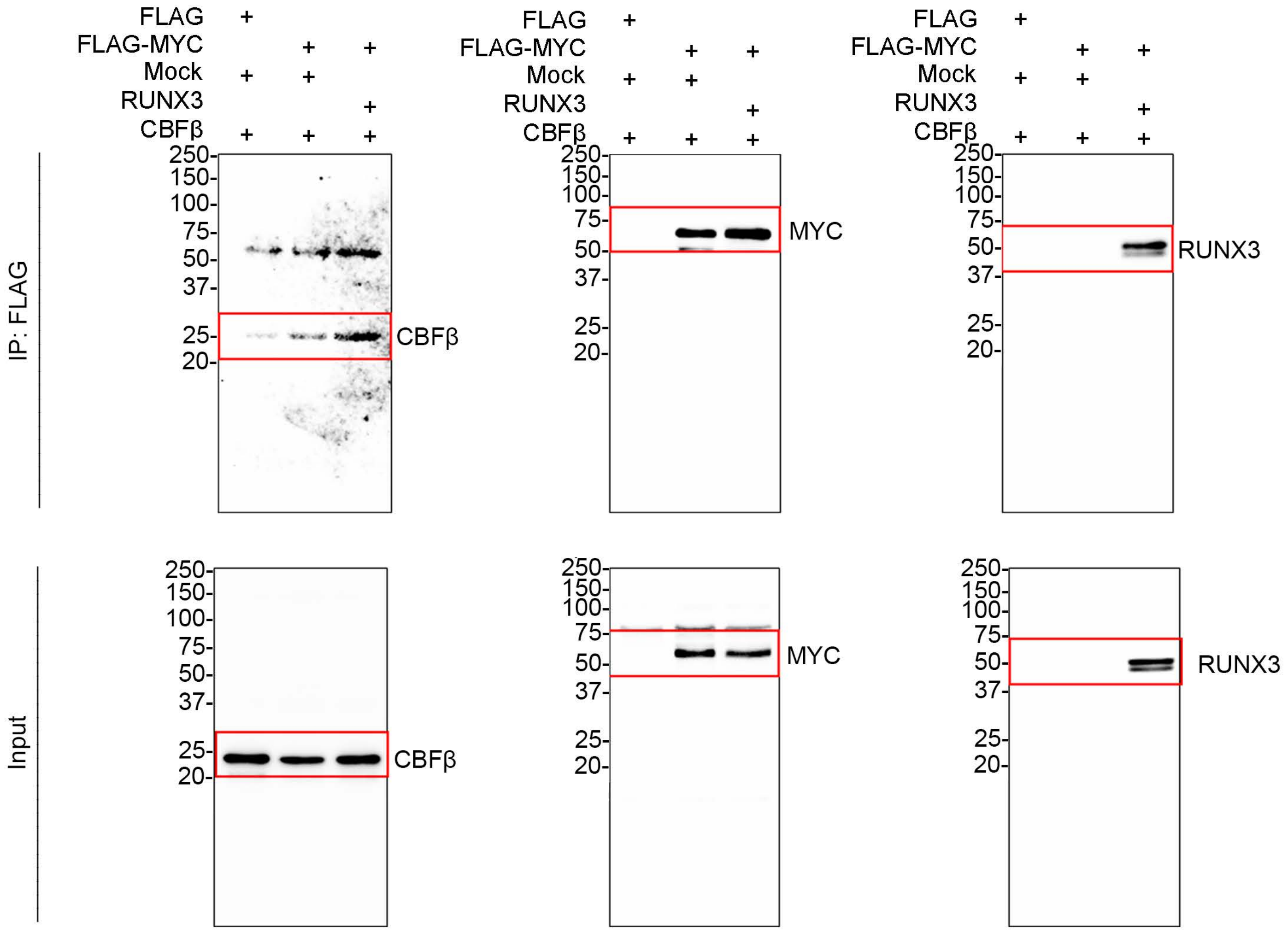

Supplementary Fig. 20: Scans of immunoblots in Figures 5a and 5b

Figure 5a

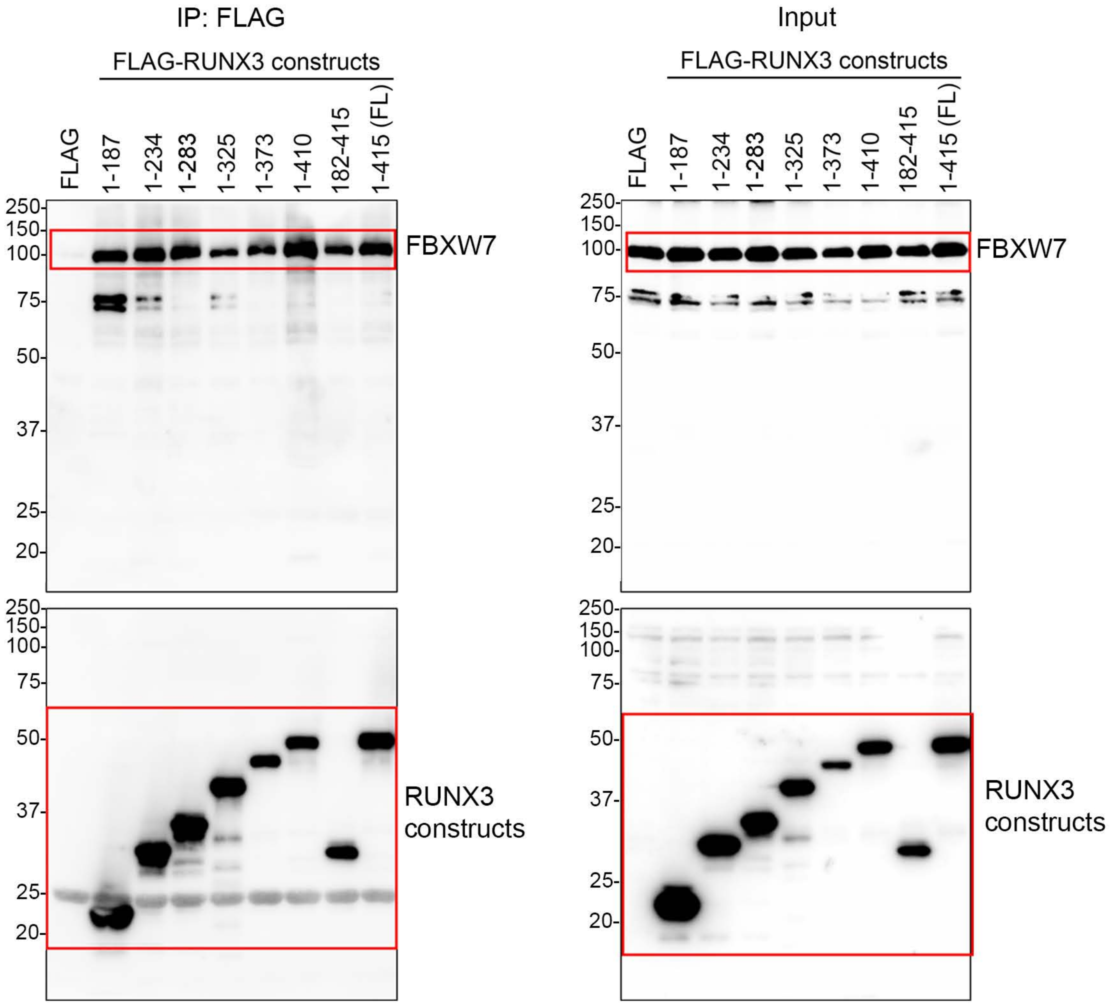

Figure 5b

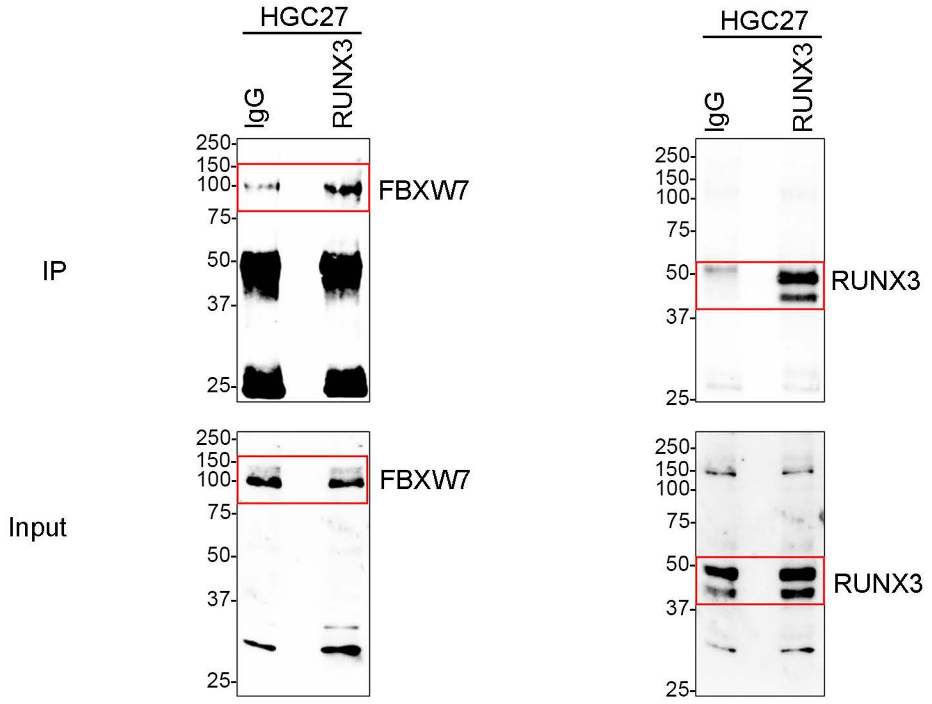

Supplementary Fig. 21: scans of immunoblots in Figures 5d

Figure 5d

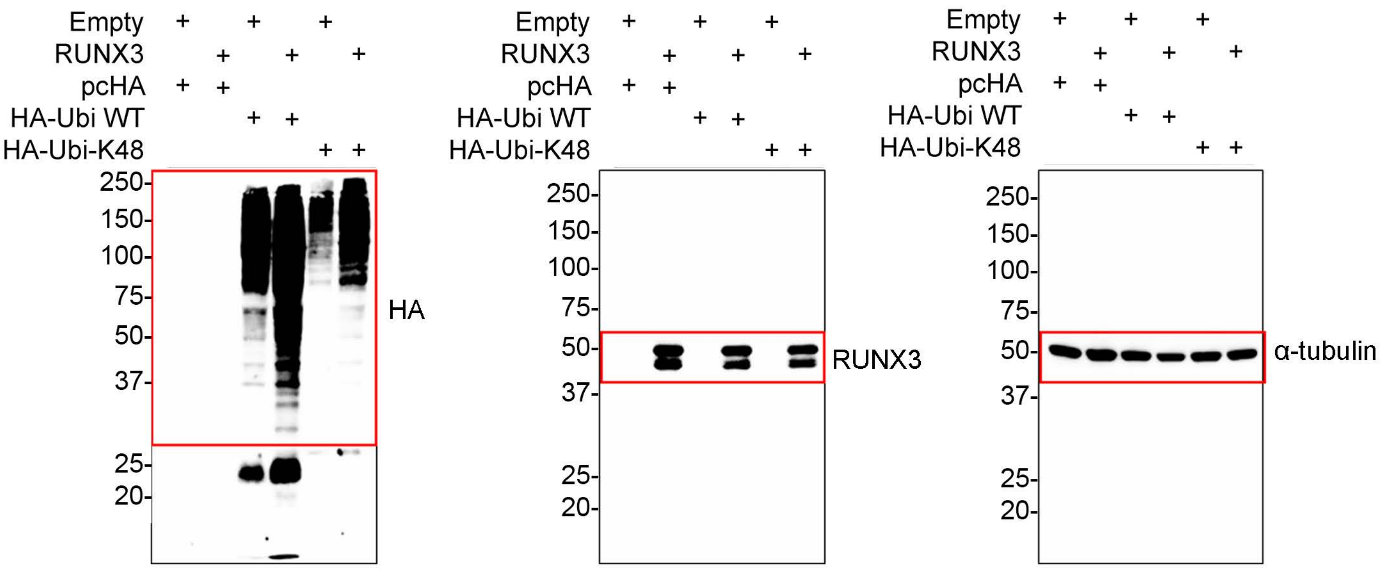

Supplementary Fig. 22: scans of immunoblots in Figures 5e

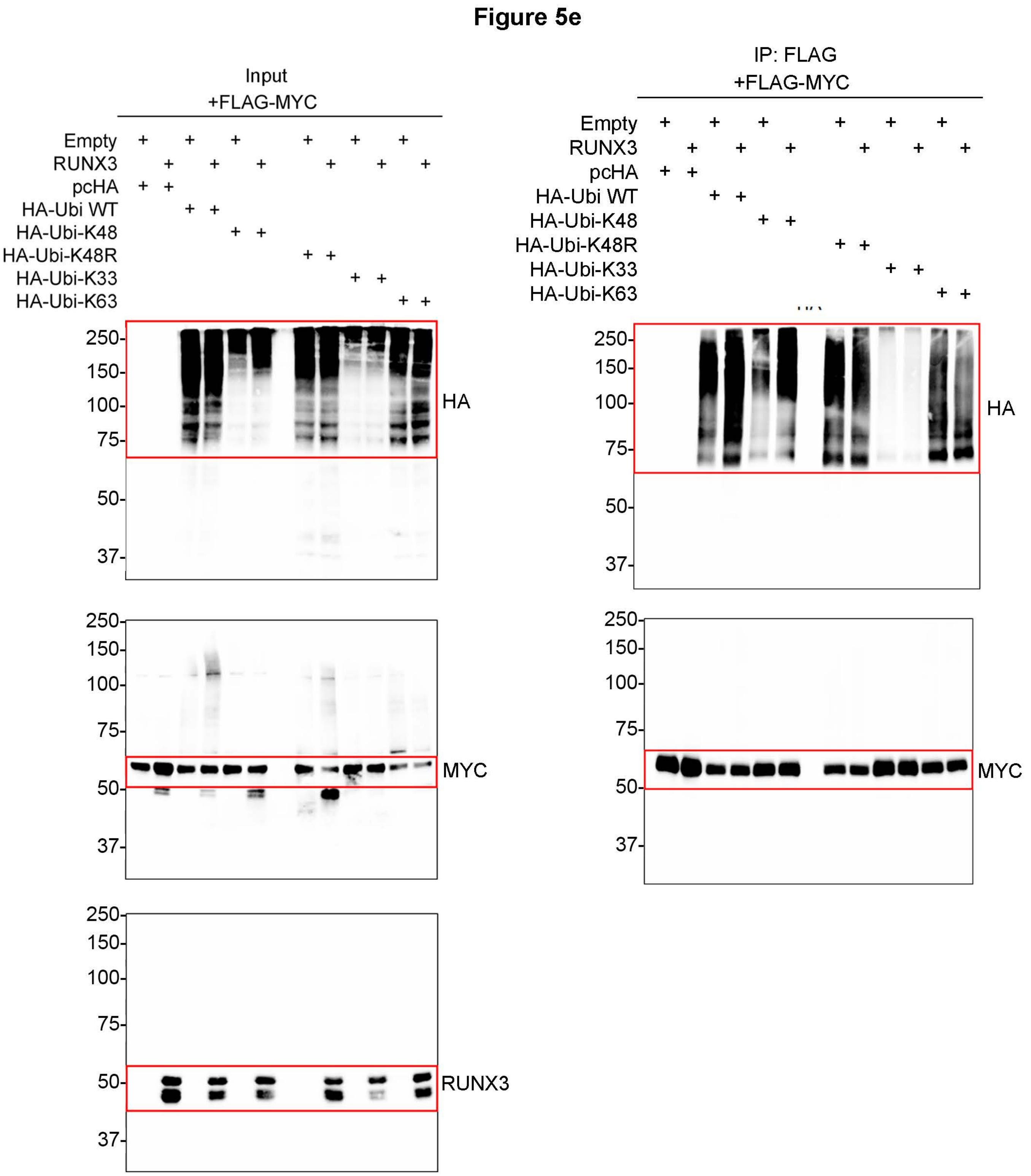

Supplmentary Fig.23: Scans of immunoblots in Figures 6a, 6b, and 6c

Figure 6a

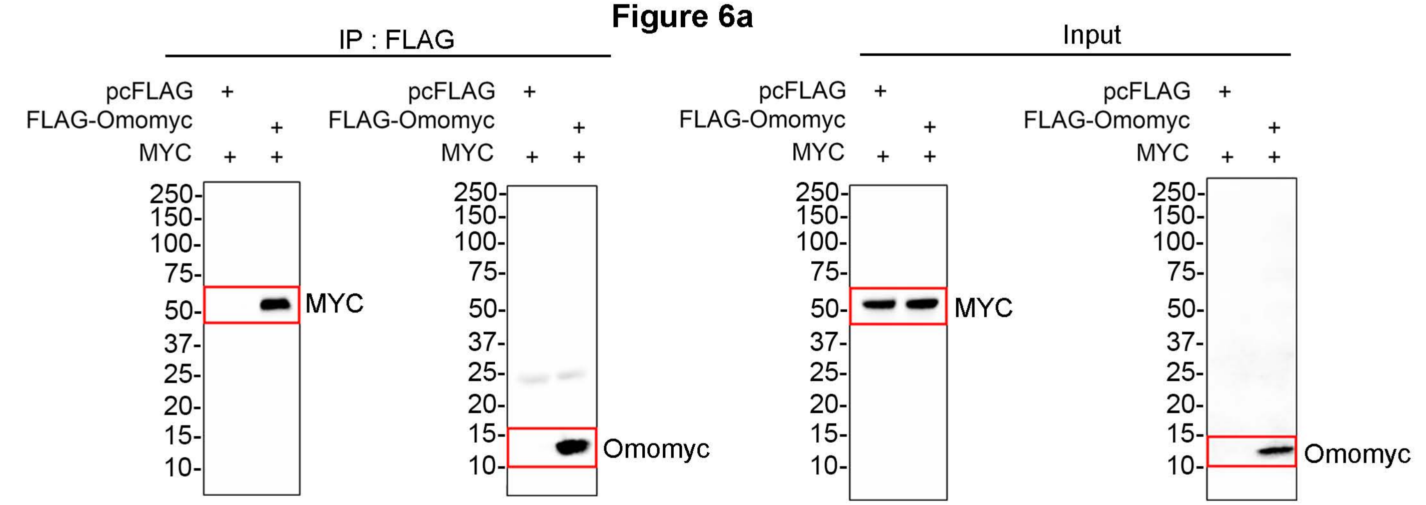

Figure 6b

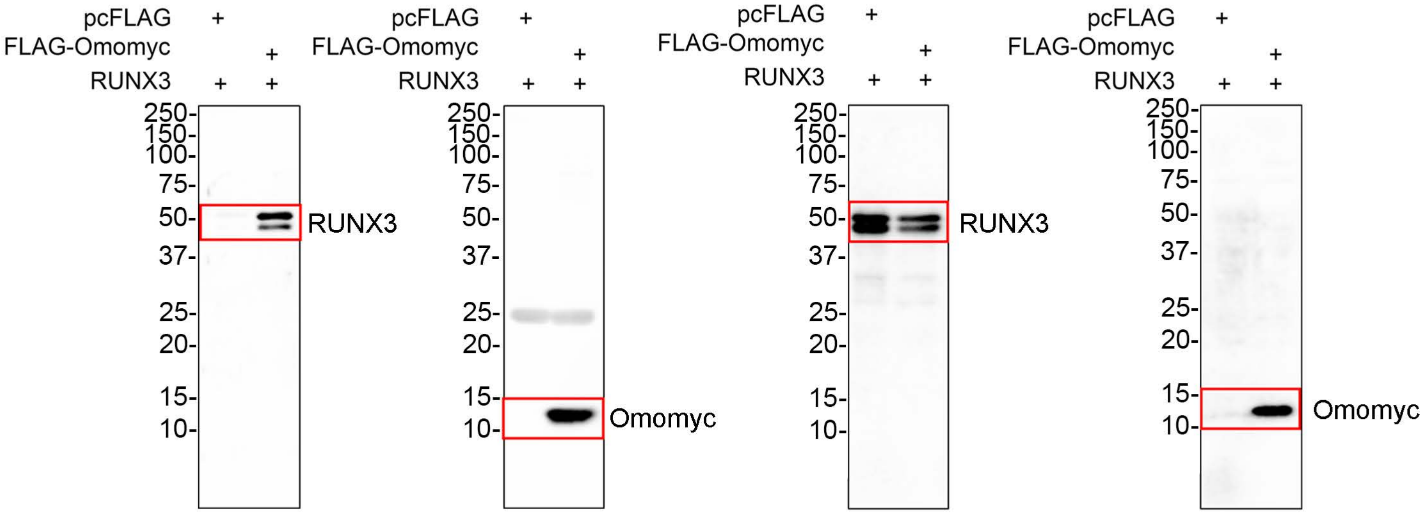

Figure 6c

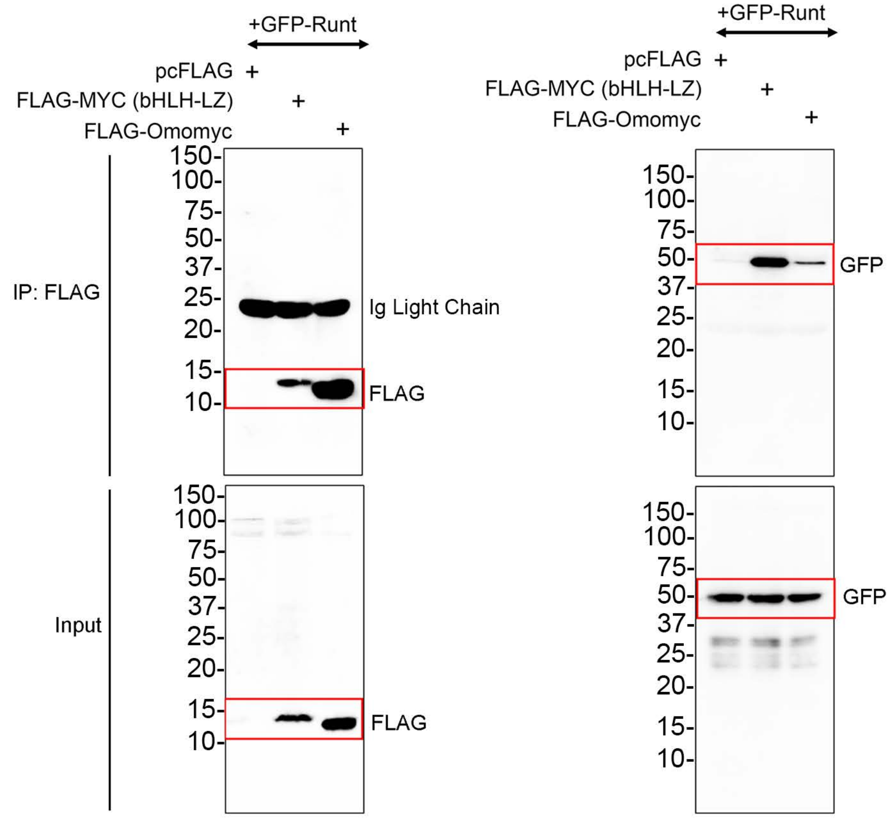

Supplementary Fig. 24: Scans of Immunoblots in Figures 6d

Figure 6d

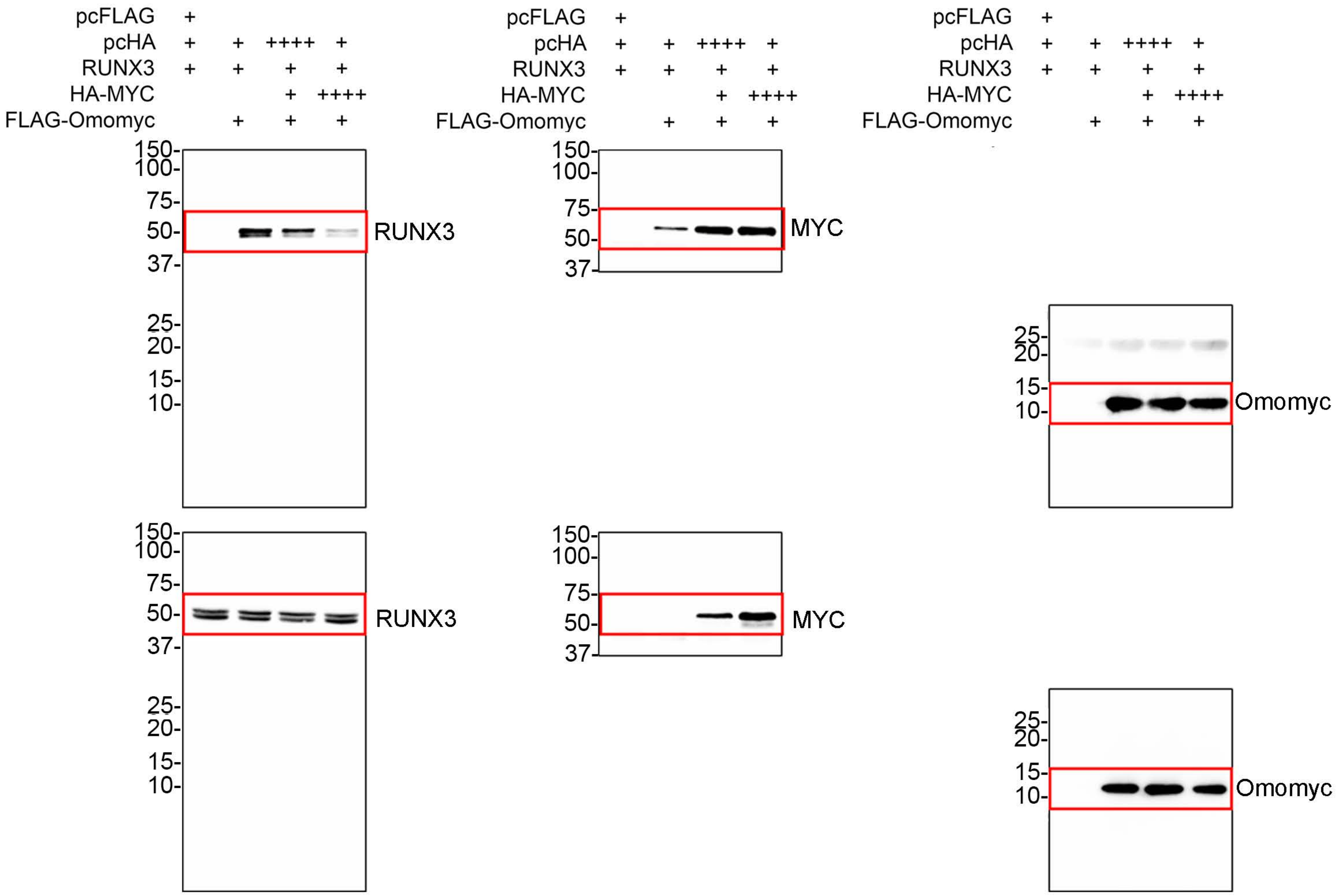

Supplementary Fig. 25: Scans of imunoblots in Figures 6e and 6f

Figure 6e

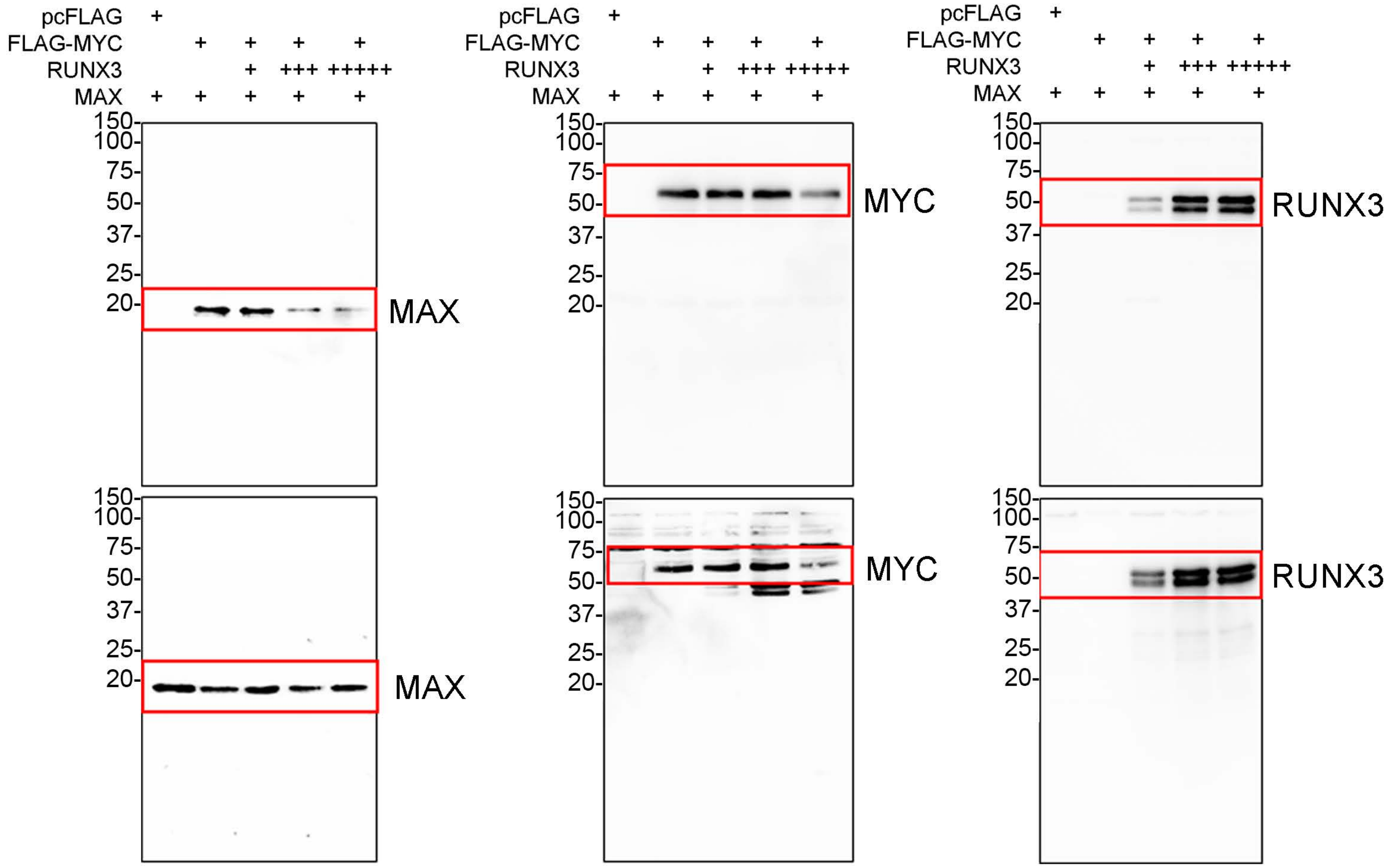

Figure 6f

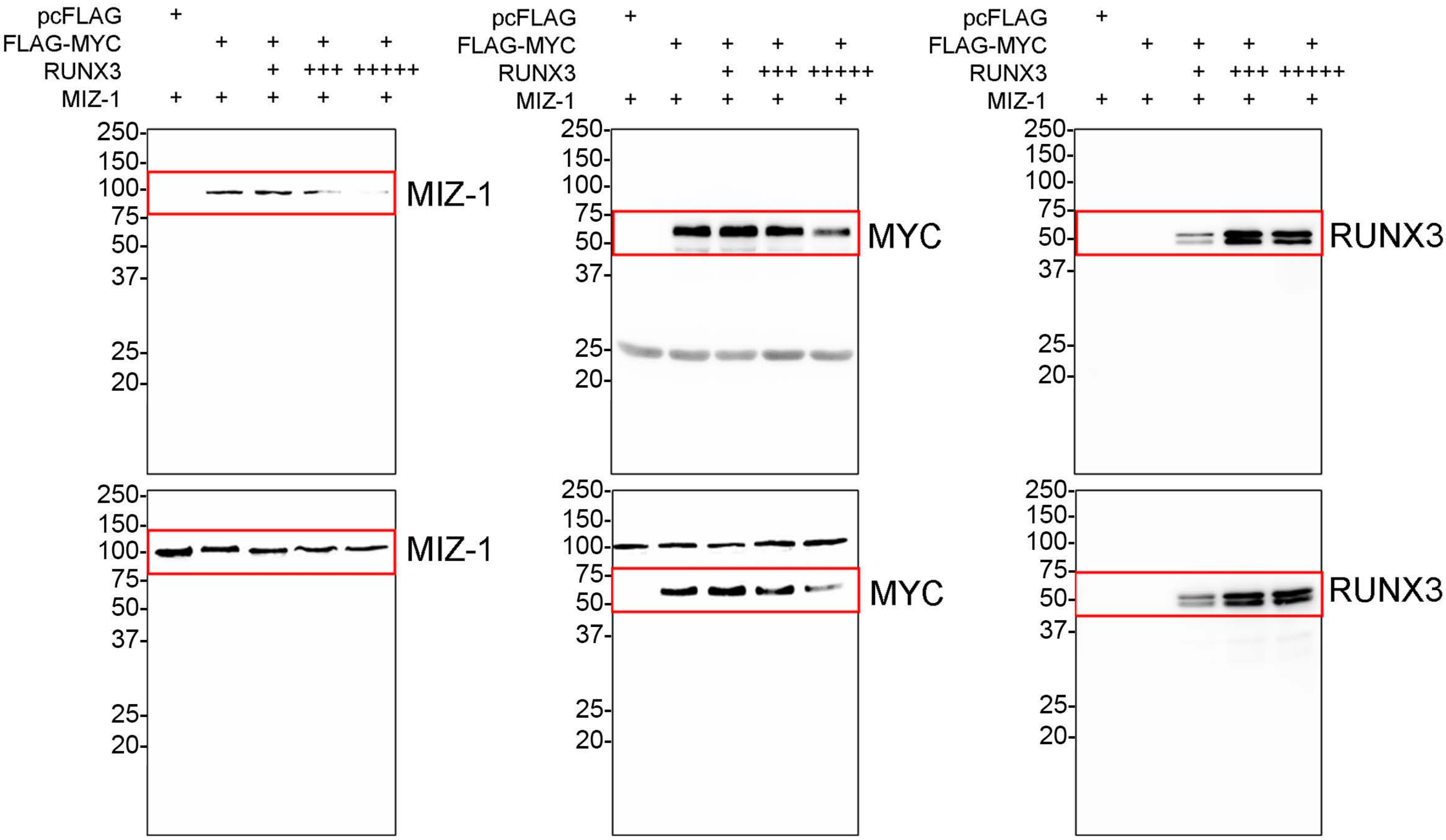

Supplementary Fig. 26: Scans of Immunoblots in Figures 6g and 6h

Figure 6g

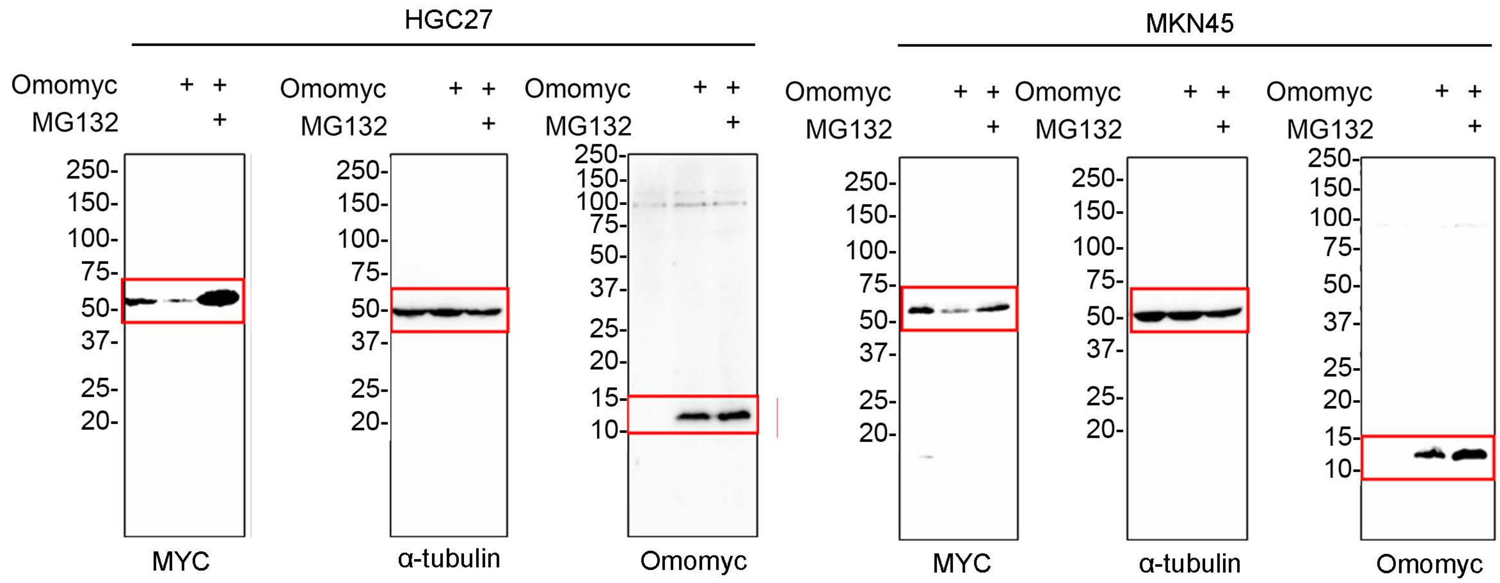

Figure 6h

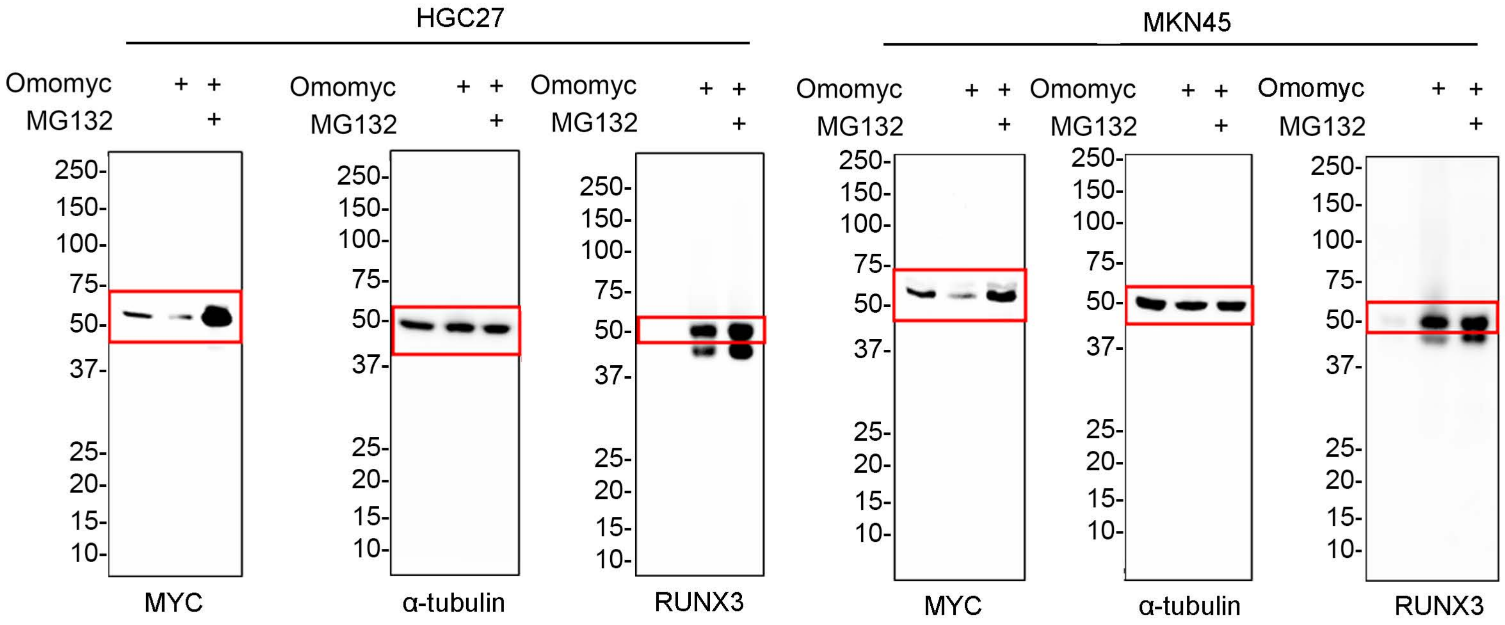

Supplementary Fig. 27: Scans of immunoblots in Supplementary Figures 1d and 1f

Supplementary figure 1d

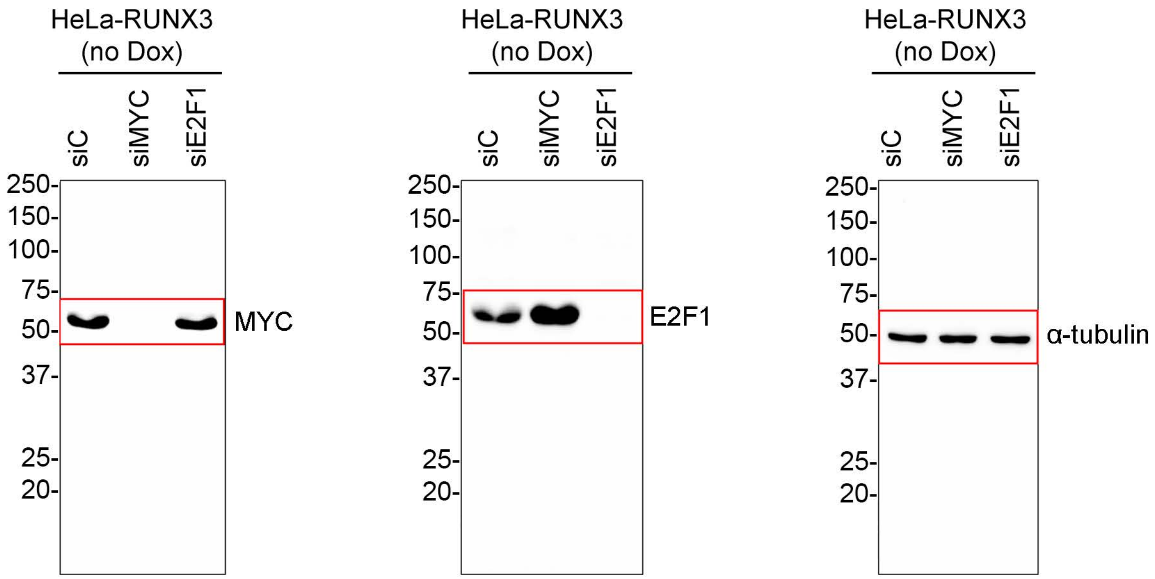

Supplementary figure 1f

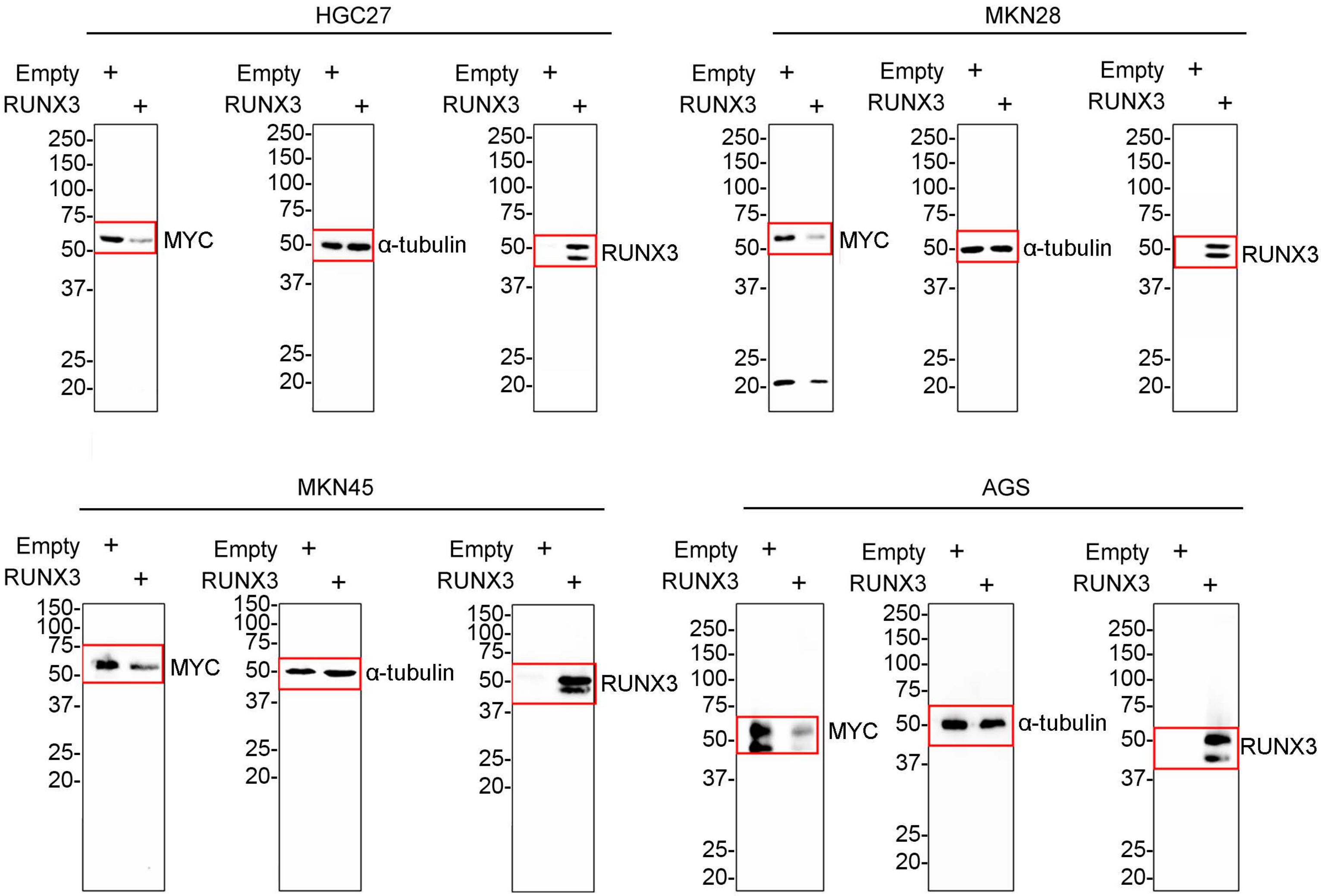

Supplementary Fig. 28: Scans of immunoblots from Supplementary Figure 3a

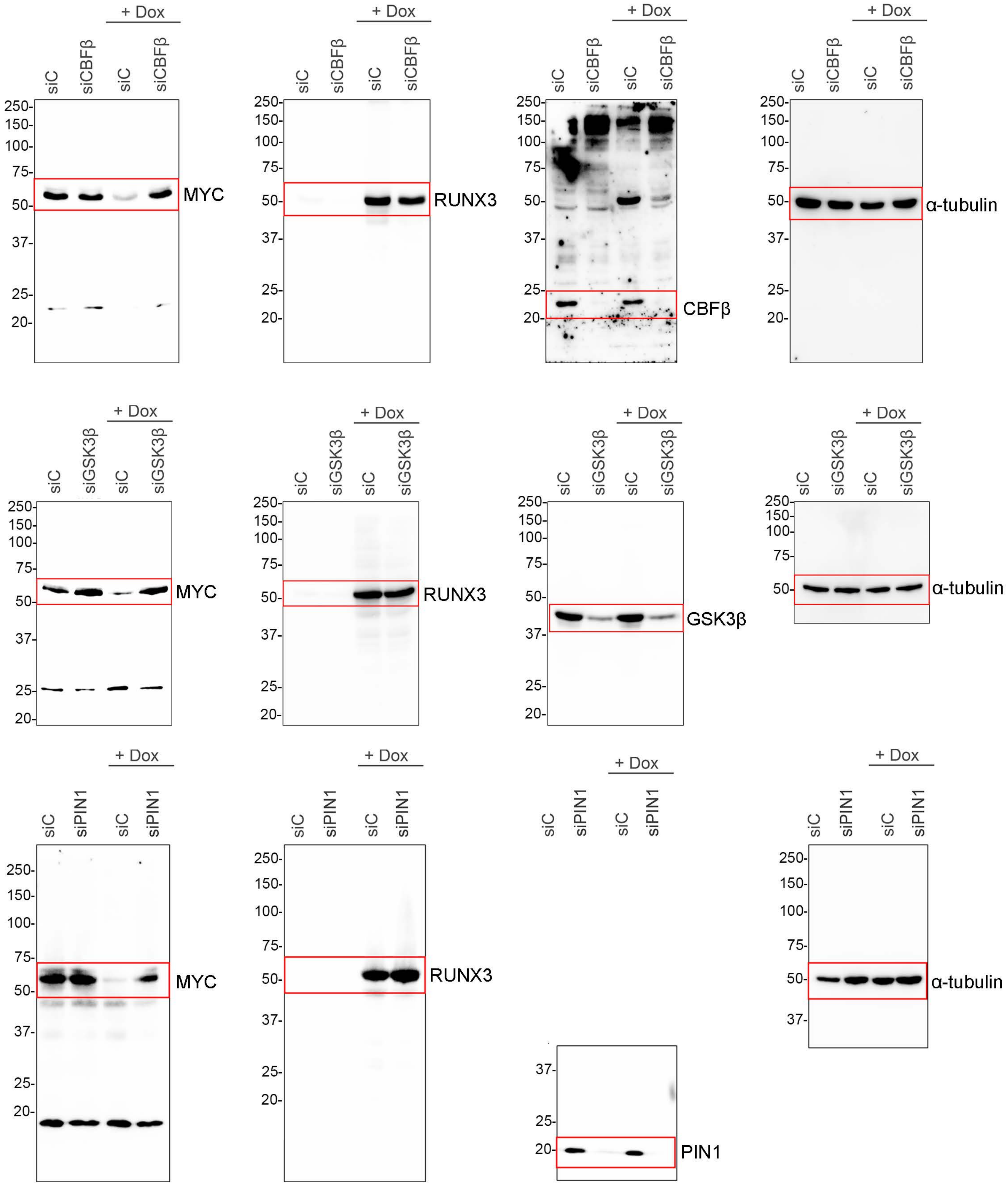

Supplementary Fig. 29: Scans of Immunoblots from Supplementary Figures 3a, 4a and 4c

Supplementary Figure 3a

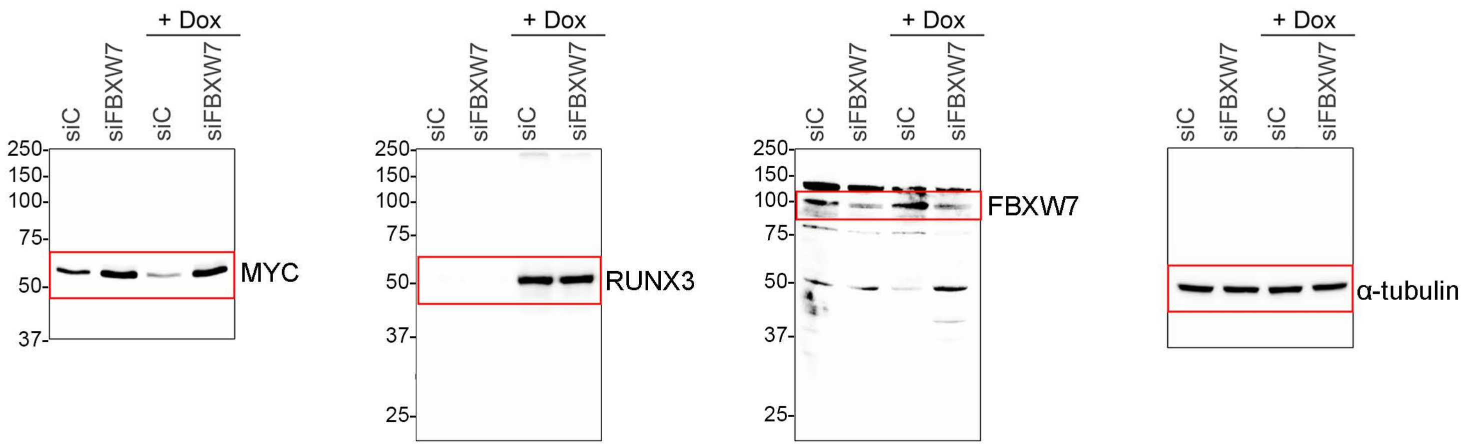

Supplementary Figure 4a

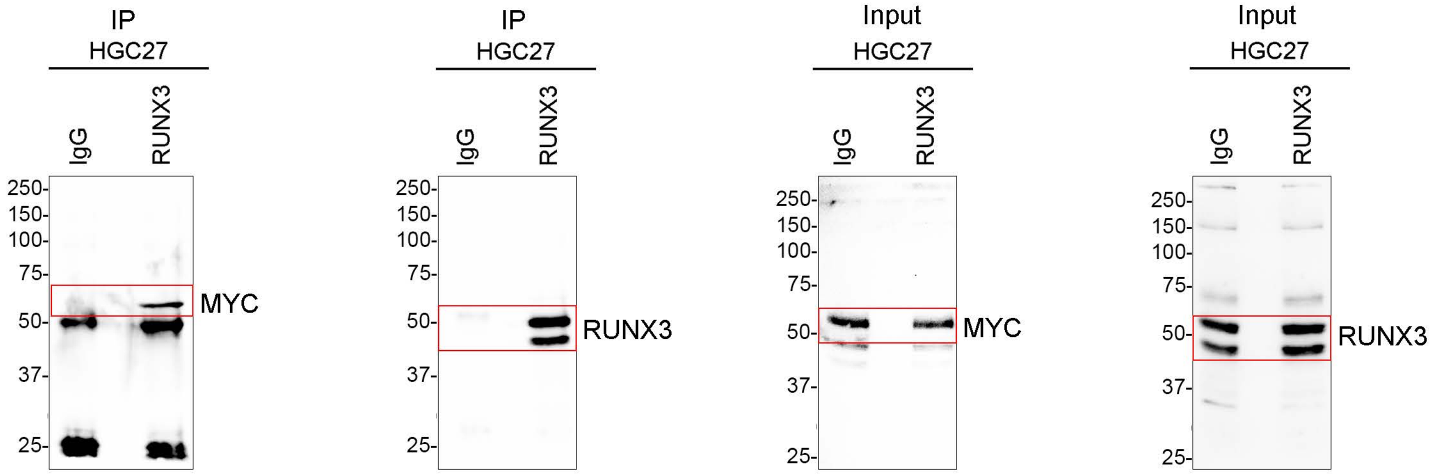

Supplementary Figure 4c

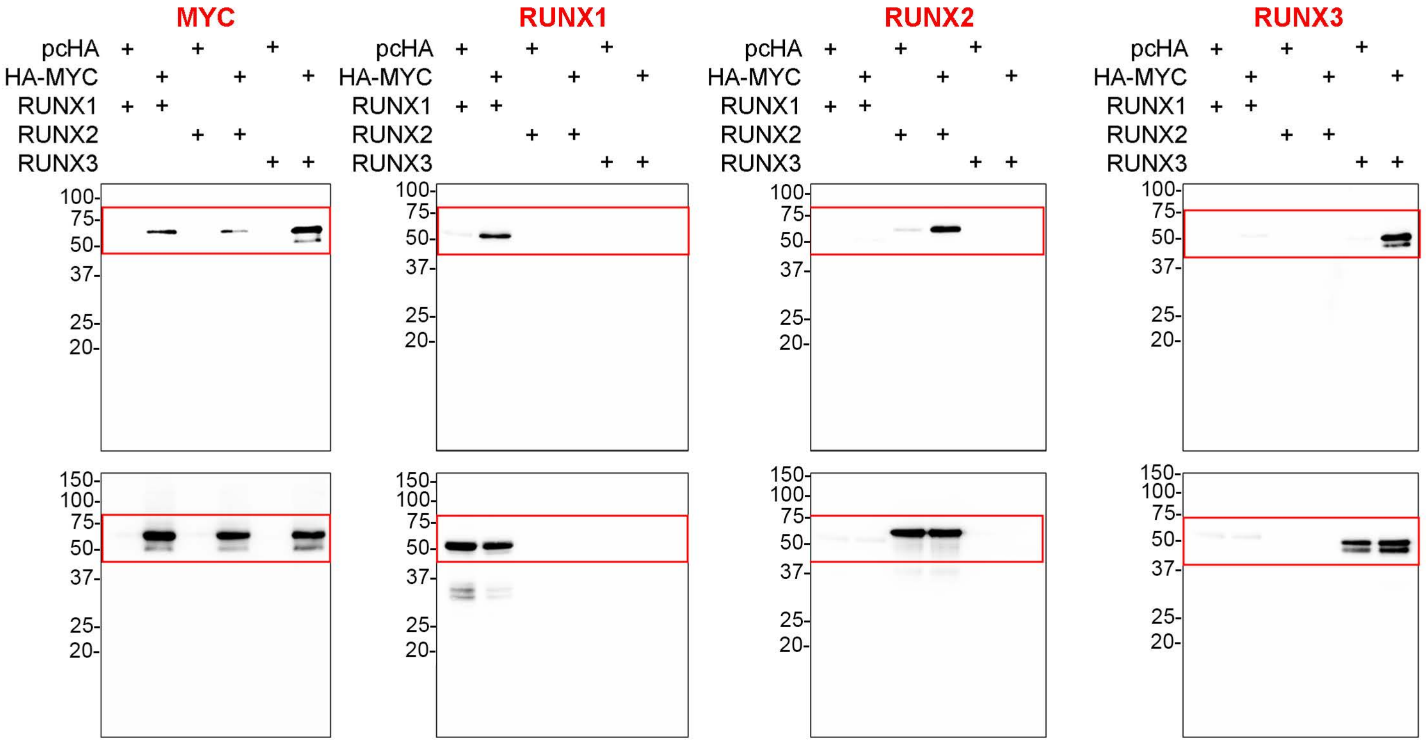

Supplementary Fig. 30: Scans of Immunoblots from Supplementary Figures 4d and 4e

Supplementary Figure 4d

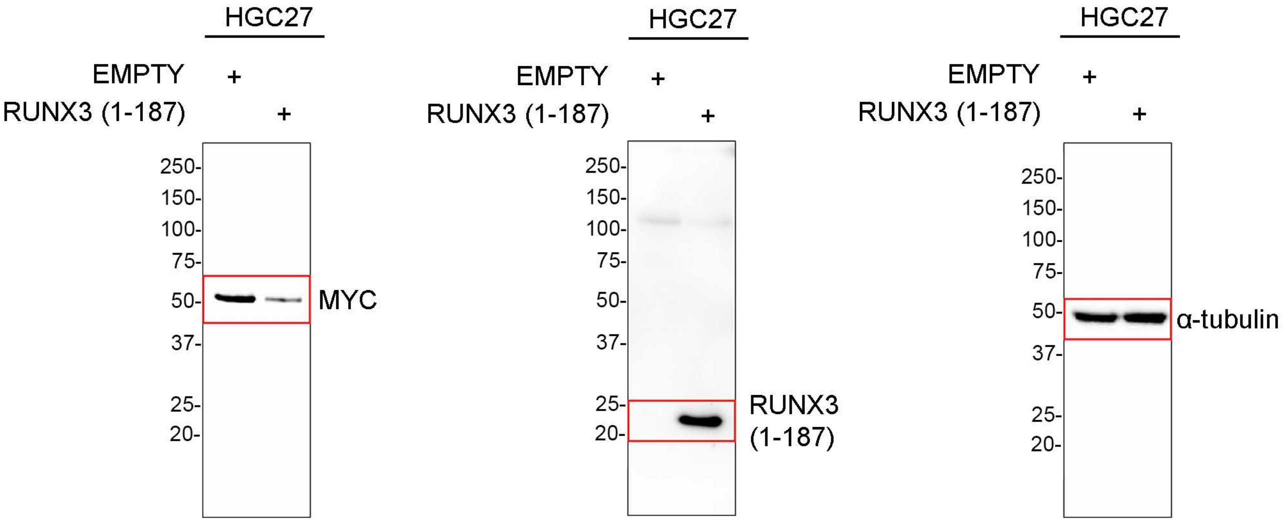

Supplementary Figure 4e

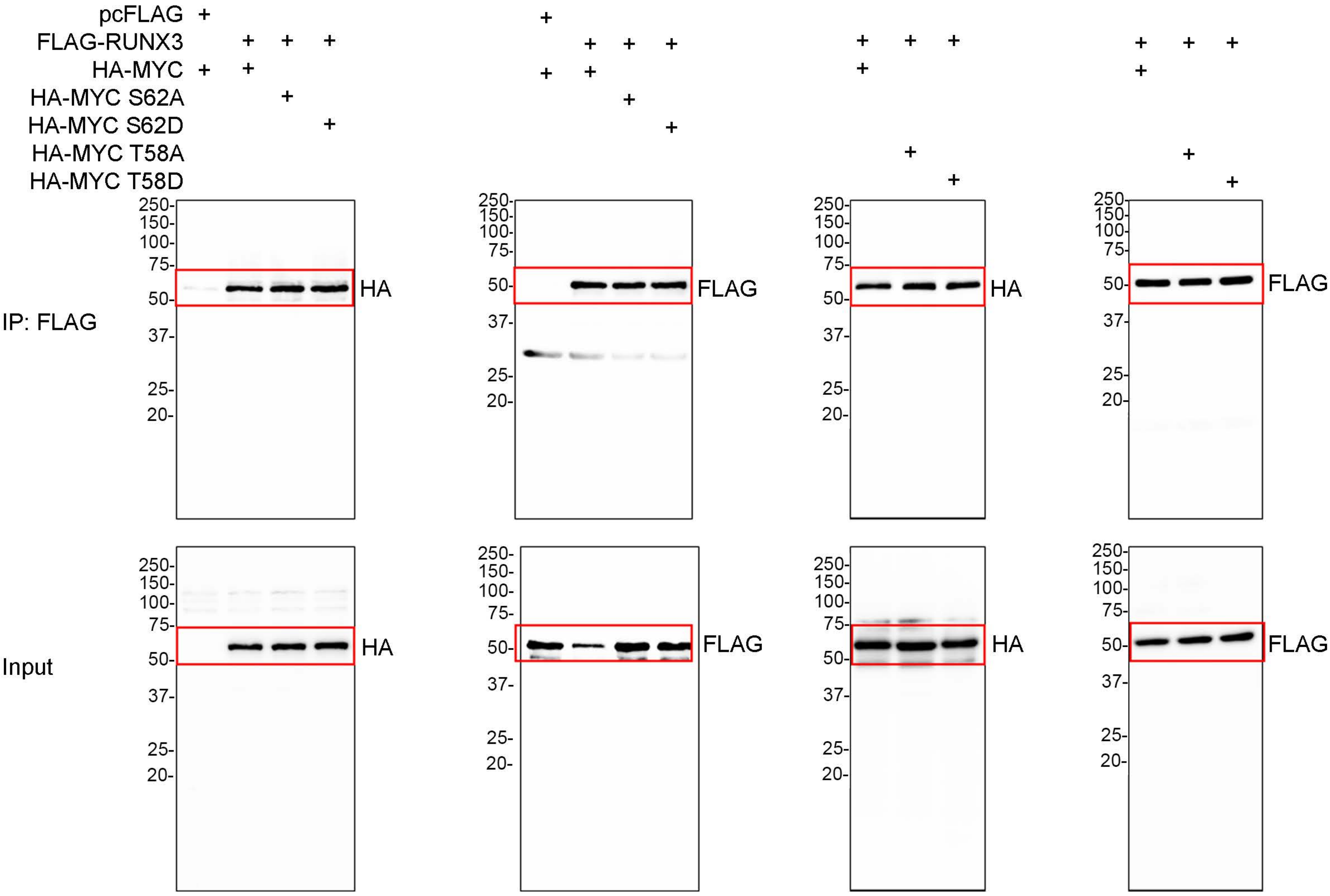

Supplementary Fig. 31: Scans of Immunoblots from Supplementary Figures 5a and 5b

Supplementary Figure 5a

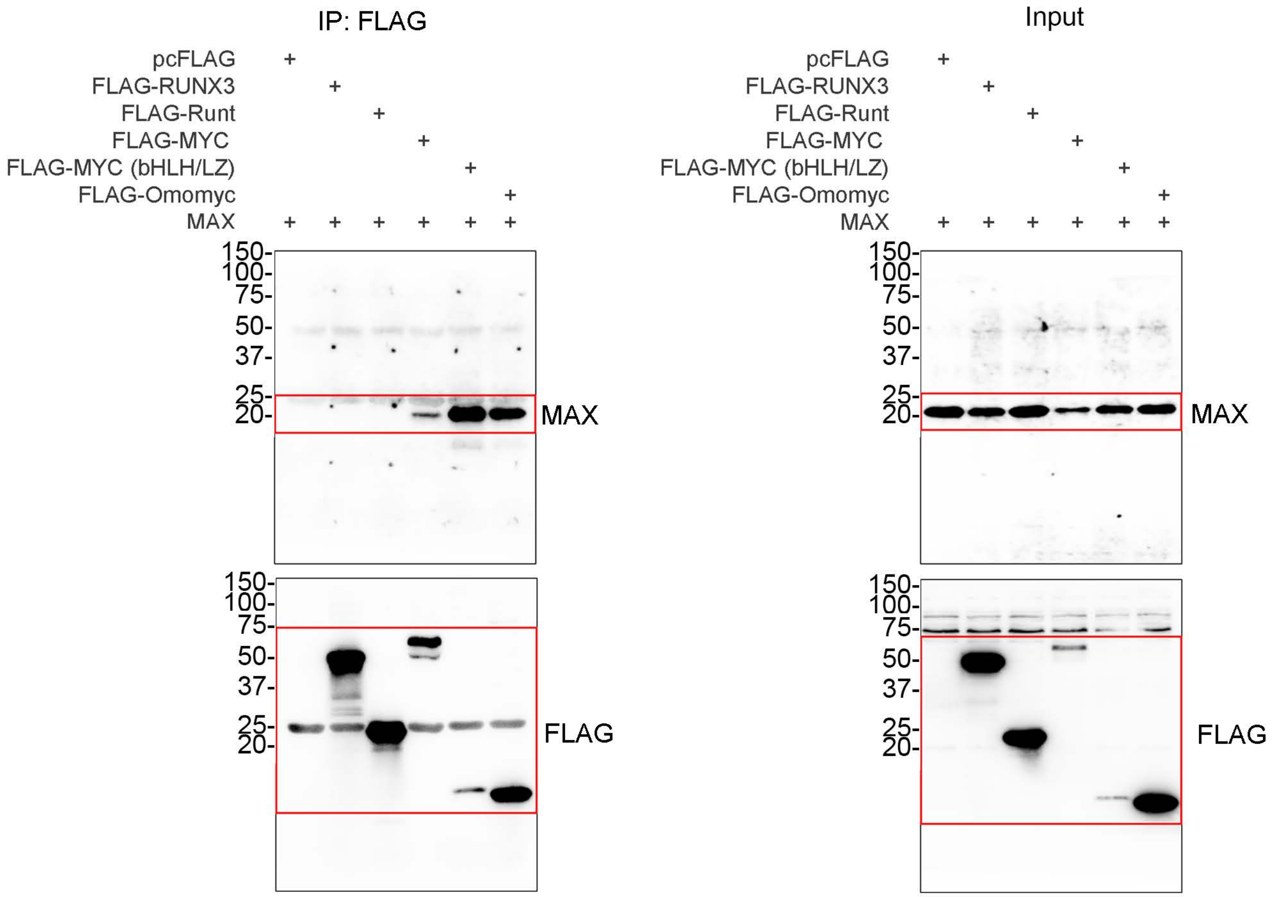

Supplementary Figure 5b

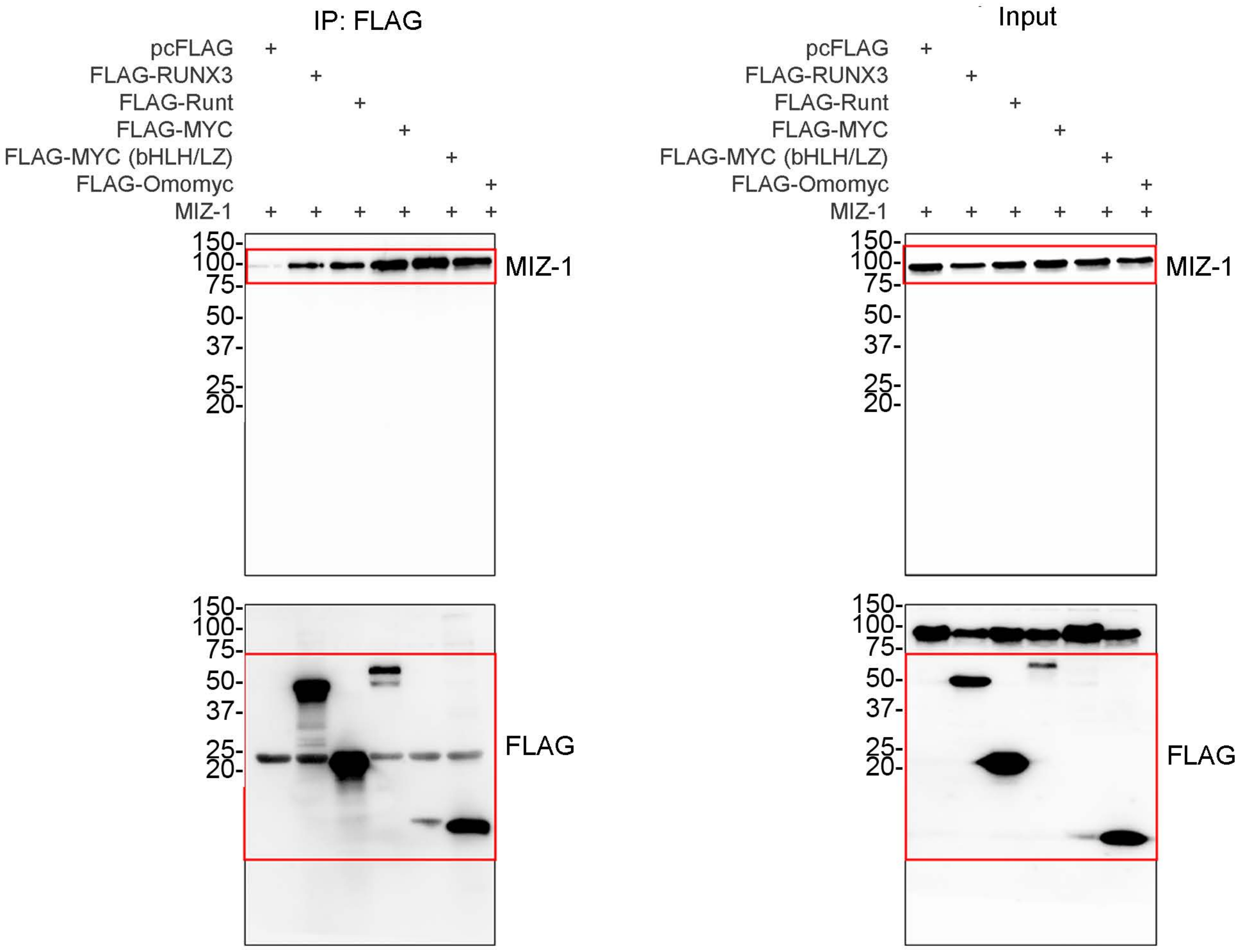

Supplementary Fig. 32: Scans of immunoblots from Supplementary Figure 5c

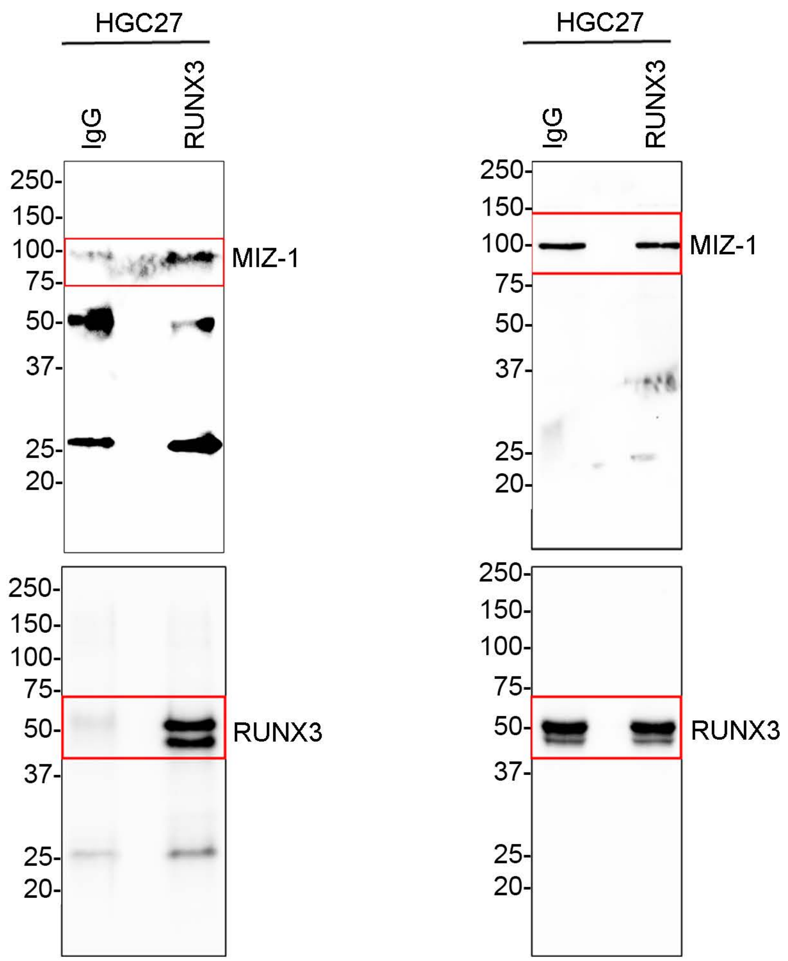

Supplementary Table 1: List of antibodies, plasmids, cell lines, reagents, primers, softwares and algorithms.

Reagents and Materials	Source	Identifier
**1° Antibodies**		
CBFβ Rabbit mAb	Abcam	Cat# ab133600
c-MYC (D84C12) Rabbit mAb	Cell Signaling Technology	Cat# 5605
c-MYC (OTI3F2) Mouse mAb	Origene	Cat# TA500003
c-MYC (phospho S62) Rabbit mAb	Cell Signaling Technology	Cat# 13748
c-MYC (phospho T58) Rabbit mAb	Abcam	Cat# ab185655
E2F-1 Rabbit Antibody	Cell Signaling Technology	Cat# 3742
FBXW7 Rabbit Antibody	proteintech	Cat# 55290
FLAG M2 Mouse mAb	Sigma-Aldrich	Cat# F1804
FLAG Rabbit antibody	Sigma-Aldrich	Cat# F7425
GAPDH (14C10) Rabbit mAb	Cell Signaling Technology	Cat# 2118
GSK-3β (D5C5Z) XP® Rabbit mAb	Cell Signaling Technology	Cat# 12456
GST Mouse mAb (B-14)	Santa Cruz Biotechnology	Cat# sc-138
HA-Tag (C29F4) Rabbit mAb	Sigma-Aldrich	Cat# 3724
K48-linkage Specific Polyubiquitin (D9D5) Rabbit mAb	Cell Signaling Technology	Cat# 8081
MAX antibody Rabbit mAb	Abcam	Cat# ab199489
Miz-1 (D7E8B) Rabbit mAb	Cell Signaling Technology	Cat# 14300
Normal Rabbit IgG Antibody	Cell Signaling Technology	Cat# 2729
p21 Waf1/Cip1 (12D1) Rabbit mAb	Cell Signaling Technology	Cat# 2947
p53 Mouse mAb (DO-1)	Santa Cruz Biotechnology	Cat# sc-126
Pin1 Mouse Antibody (G-8)	Santa Cruz Biotechnology	Cat# sc-46660
PP2A C Subunit Rabbit Antibody	Cell Signaling Technology	Cat# 2038
RUNX1 (D33G6) Rabbit mAb	Cell Signaling Technology	Cat# 4336
RUNX2 (D1H7) Rabbit mAb	Cell Signaling Technology	Cat# 8486
RUNX3 (D6E2) Rabbit mAb	Cell Signaling Technology	Cat# 9647
RUNX3 (D9K6L) Mouse mAb	Cell Signaling Technology	Cat# 13089
α-Tubulin Mouse mAb	Sigma-Aldrich	Cat# T9026
β-Tubulin (9F3) Rabbit mAb (HRP Conjugate)	Cell Signaling Technology	Cat# 5346
**2° Antibodies** **For Immunofluorescence**		
Goat anti-Mouse IgG (H+L) Highly Cross-Adsorbed Secondary Antibody, Alexa Fluor 546	Invitrogen	Cat# A11030
Donkey anti-Mouse IgG (H+L) Highly Cross-Adsorbed Secondary Antibody, Alexa Fluor 488	Invitrogen	Cat# A21202
Donkey anti-Rabbit IgG (H+L) Highly Cross-Adsorbed Secondary Antibody, Alexa Fluor 488	Invitrogen	Cat# A21206

Donkey anti-Rabbit IgG (H+L) Highly Cross-Adsorbed Secondary Antibody, Alexa Fluor 555	Invitrogen	Cat# A31572
**For Immunoblot**		
Amersham Horseradish Peroxidase ECL Anti-Mouse IgG	Rockland	Cat# NA931
Amersham ECL Donkey Anti-Rabbit IgG (HRP-linked whole Ab), for Western blotting, 1 ml	GE	Cat# NA934
Mouse TrueBlot® ULTRA: Anti-Mouse Ig HRP	Invitrogen	Cat# 18-8817-33
**Beads for Immunoprecipitation**		
Glutathione Sepharose 4 Fast Flow, 25 mL	Cytiva	Cat# 17513201
Dynabeads Protein G for Immunoprecipitation, 5ml	Invitrogen	Cat# 10004D
EZview™ Red Anti-HA Affinity Gel	Sigma-Aldrich	Cat# E6779
FLAG® M2 Affinity Gel	Sigma-Aldrich	Cat# A2220
**Commercial Kits**		
Bio-Rad Protein Assay Dye Reagent Concentrate	Bio-rad	Cat# 5000006
Click-iT™ EdU Cell Proliferation Kit for Imaging	Invitrogen	Cat# C10340
Duolink™ In Situ Detection Reagents Green	Sigma-Aldrich	Cat# DUO92014
Duolink™ In Situ Mounting Medium with DAPI	Sigma-Aldrich	Cat# DUO82040
Duolink™ In Situ PLA® Probe Anti-Mouse PLUS	Sigma-Aldrich	Cat# DUO92001
Duolink™ In Situ PLA® Probe Anti-Rabbit MINUS	Sigma-Aldrich	Cat# DUO92005
Duolink™ In Situ Wash Buffers, Fluorescence	Sigma-Aldrich	Cat# DUO82049
High-Capacity cDNA Reverse Transcription Kit	Applied Biosystems™	Cat# 4368814
KAPA HiFi+dNTPs (250U)	KAPA	Cat# 7958846001
LOOKOUT MYCOPLASMA PCR DETECTION KIT	Sigma-Aldrich	Cat# MP0035
Pierce™ BCA Protein Assay Kit	Thermofisher scientific	Cat# 23225
QIAfilter Plasmid Maxi Kit (25)	Qiagen	Cat# 12263
QIAprep Spin Miniprep Kit (250)	Qiagen	Cat# 27106
QIAquick Gel Extraction Kit (50)	Qiagen	Cat# 28704
QIAquick Spin Columns (100)	Qiagen	Cat# 28115
RNeasy Mini Kit (250)	Qiagen	Cat# 74106
TNT® T7 Quick Coupled Transcription/ Translation System	Promega	Cat# L1170
**Drugs and Transfection Reagents**		
Ampicillin sodium salt	Sigma-Aldrich	Cat# A9518
Cycloheximide	Sigma-Aldrich	Cat# C7698
DIMETHYL SULFOXIDE HYBRI-MAX STERILE	Sigma-Aldrich	Cat# D2650
Doxycycline Hyclate	Sigma-Aldrich	Cat# D9891-1g
Geneticin™ Selective Antibiotic (G418 Sulfate) (50 mg/mL)	Gibco	Cat# 10131-027
IPTG	Sigma-Aldrich	Cat# I6758
jetPRIME DNA and siRNA Transfection Reagent	Polyplus	Cat# 114-75
Kanamycin sulfate from Streptomyces kanamyceticus	Sigma-Aldrich	Cat# K4000

MirusBio TransIT-LT1 Transfection Reagent, Size 5x1ml	MirusBio	Cat# MIR2305
Polybrene Transfection Reagent, 1 ml	Millipore	Cat# TR-1003
Puromycin Dihydrochloride	Gibco	Cat# A1113803
Z-Leu-Leu-Leu-al (MG132)	Sigma-Aldrich	Cat# C2211
**Enzyme and Recombinant protein**		
BamHI-HF®	NEB	Cat# R3136S
Benzonase® Nuclease, Purity > 99%	Merck Millipore	Cat# 70664
DNase I recombinant, RNase-free	Roche	Cat# 04 716 728 001
DpnI	NEB	Cat# R0176S
EcoRI-HF®	NEB	Cat# R3101S
Recombinant Human CBFb protein	Abcam	Cat# ab98252
Recombinant human GSK3 beta protein (Active)	Abcam	Cat# ab60863
Recombinant Human MYC	RayBiotech	Cat# 230-00580-50
T4 DNA Ligase, 400,000 units/ml, 20,000 units	NEB	Cat# M0202S
**Experimental Models: Cell Lines**		
AGS	ATCC	CRL-1739; RRID:CVCL_0139
HEK293T	ATCC	CRL-3216; RRID:CVCL_0063
HeLa Tet-On	Clontech	RRID:CVCL_IY74
HGC27	CellBank Australia	RRID:CVCL_1279
MKN28	JCRB Cell Bank	JCRB0253; RRID:CVCL_1416
MKN45	JCRB Cell Bank	JCRB0254; RRID:CVCL_0434
MKN28-Tet-On	This study	
**Experimental Models: Animal models**		
NOD SCID gamma (NSG) mice also known as NSG(JAX)-*NOD.Cg-Prkdc*^{scid}*Il2rg*^{tm1wjl}*SzJInv*	InVivos	RRID:IMSR_JAX:005557
**Human paraffin embedded tissue arrays**		
Stomach disease spectrum (stomach cancer progression) tissue array)	US Biomax	ST1001a, ST1001
**Softwares and Algorithms**		
Ingenuity Pathway Analysis	QIAGEN	
Flowjo	Tree Star	https://www.flowjo.com/
Graphpad Prism 7.0	Graphpad software	Graphpad Prism 7.0
Gene Set Enrichment Analysis	[1]	https://www.gsea-msigdb.org/gsea/index.jsp
Image Lab	Bio-Rad	
ImageJ	ImageJ: Image Processing and analysis in Java	https://imagej.nih.gov/ij/
QuantStudio™ Design and Analysis Software	Applied Biosystems™	
Zen 3.4 (blue edition)	Zeiss	

**cDNAs /Plasmids**	**Source**	**Identifier**

pEF-BOS CBFB type II	[2]	
pEF-BOS Mock	[3]	
pEF-BOS RUNX1	[4]	
pEF-BOS RUNX2	[2]	
pEF-BOS RUNX3	[5]	
FBXW7 cDNA ORF Clone, Human, C-HA tag	Origene	Cat# HG13414-CY
Flag-Axin1	Addgene	Cat# 109370
pcDNA-GFP RUNX3 (1-187)	This study	
HA GSK3 beta wt pcDNA3	Addgene [6]	Cat# 14753
MAX (NM_002382) Human Untagged Clone	Origene	Cat# SC111653
pCbs FlagMYC (bHLH/LZ)	This study	Addgene # 203424
pCbs FlagOmomyc	Addgene	Cat# 113168
pcDNA HA	This study	
pCDNA3-deltaBamHI-human MIZ1	Addgene [7].	Cat# 74167
pCDNA3-HA-HA-humanCMYC (116-439)	This study	Addgene # 203425
pCDNA3-HA-HA-humanCMYC (1-170) T4	This study	Addgene # 203426
pCDNA3-HA-HA-humanCMYC (1-208)	This study	Addgene # 203427
pCDNA3-HA-HA-humanCMYC (1-277)	This study	Addgene # 203428
pCDNA3-HA-HA-humanCMYC (1-357)	This study	Addgene # 203429
pCDNA3-HA-HA-humanCMYC (170-439)	This study	Addgene # 203430
pCDNA3-HA-HA-humanCMYC (201-298)	This study	Addgene # 203431
pCDNA3-HA-HA-humanCMYC (208-300) t4	This study	Addgene # 203432
pCDNA3-HA-HA-humanCMYC (208-439) t4	This study	Addgene # 203433
pCDNA3-HA-HA-humanCMYC (277-357) t4	This study	Addgene # 203434
pCDNA3-HA-HA-humanCMYC (277-439) t4	This study	Addgene # 203435
pCDNA3-HA-HA-humanCMYC plasmid	Addgene	Cat# 74164
pCDNA3-HA-HA-humanCMYC plasmid S62A	This study	Addgene # 203436
pCDNA3-HA-HA-humanCMYC plasmid S62D	This study	Addgene # 203437
pcDNA3-FLAG (pcFLAG)	[8]	
pcFLAG RUNX3	[8]	
pcFLAG RUNX3 (1-187)	[8]	
pcFLAG RUNX3 (1-234)	[8]	
pcFLAG RUNX3 (1-283)	[8]	
pcFLAG RUNX3 (1-325)	[8]	
pcFLAG RUNX3 (1-373)	[8]	
pcFLAG RUNX3 (1-410)	[8]	
pcFLAG RUNX3 (182-415)	[8]	
pcFLAG RUNX3 (R122C)	This study	Addgene # 203438
pCMV4a-Flag-c-Myc FL	Addgene [9]	102625
pEGFP C-1	Clontech	
pRK5-HA-Ubiquitin-K33	Addgene [10]	17607
pRK5-HA-Ubiquitin-K48	Addgene [10]	17605
pRK5-HA-Ubiquitin-K48R	Addgene [10]	17604
pRK5-HA-Ubiquitin-K63	Addgene [10]	17606
pRK5-HA-Ubiquitin-WT	Addgene [10]	17608

V245 pCEP-4HA B56alpha	Addgene [11]	14532
pGEX4T-1	GE Healthcare	
pGEX4T-1-RUNX3 aa 49-187	This study	Addgene # 203440
**Retroviral Plasmid**		
pRetroX-Tet-On Advanced Vector	Clontech	PT3968-5
pRetroX-Tight-puro-3×FLAG	[12]	
pRetroX-Tight-puro-3xFLAG-RUNX3	[13]	
pRetroX-Tight-puro-3xFLAG-RUNX3 R122C	This study	Addgene # 203439

Primers for site directed mutagenesis	Oligonucleotides sequences
Flag_Omomyc_DR_F	5' GACTTGTTGCGGAAACGACGAGAACAGTTGAAACAC 3'
Flag_Omomyc_DR_R	5' GTGTTTCAACTGTTCTCGTCGTTTCCGCAACAAGTC 3'
Flag_Omomyc_I417E_F	5' GCTCATTTCTGAAGAGGACTTGTTGCGG 3'
Flag_Omomyc_I417E_R	5' CCGCAACAAGTCCTCTTCAGAAATGAGC 3'
Flag_Omomyc_T410E_F	5' GTCCAAGCAGAGGAGCAAAAGCTCATTTCTG 3'
Flag_Omomyc_T410E_R	5' CAGAAATGAGCTTTTGCTCCTCTGCTTGGAC 3'
Flag_RUNX3_1-187_F	5'CACCGGCAGAAGCTGTGAGACCAGACCAAGCCG 3'
Flag_RUNX3_1-187_R	5'CGGCTTGGTCTGGTCTCACAGCTTCTGCCGGTG 3'
Flag_RUNX3_R122C_F	5'-CTCCGCTGAGCTGTGCAATGCCTCGGCCGTC-3'
Flag_RUNX3_R122C_R	5'-GACGGCCGAGGCATTGCACAGCTCAGCGGAG-3'
pCDNA3-HA-HA-MYC 116_439R	5'-GTCGTCCGGGTCGCAGATGGATCCGGCGTAGTCGGG-3'
pCDNA3-HA-HA-MYC 116-439F	5'-CCCGACTACGCCGGATCCATCTGCGACCCGGACGAC-3'
pCDNA3-HA-HA-MYC 1-171_F T4	5'-CGCCGGATCCATGCCCCTCAACGTTAGCTTC-3'
pCDNA3-HA-HA-MYC 1-171_R T4	5'-CTGCAGAATTCTCAGACGCTGTGGCCGCGGGC-3'
pCDNA3-HA-HA-MYC 1-208_F	5'-CTCGCCCAAGTCCTGAGCCTCGCAAGACTCC-3'
pCDNA3-HA-HA-MYC 1-208_R	5'-GGAGTCTTGCGAGGCTCAGGACTTGGGCGAG-3'
pCDNA3-HA-HA-MYC 1-277_F	5'-GCTCCTGGCAAAAGGTGAGAGTCTGGATCACC 3'
pCDNA3-HA-HA-MYC 1-277_R	5'-GGTGATCCAGACTCTCACCTTTTGCCAGGAGC-3'
pCDNA3-HA-HA-MYC 1-357_F	5'-GAGAATGTCAAGAGGTGAACACACAACGTCTTG-3'
pCDNA3-HA-HA-MYC 1-357_R	5'-CAAGACGTTGTGTGTTCACCTCTTGACATTCTC -3'
pCDNA3-HA-HA-MYC 170-439F	5'-CCCGACTACGCCGGATCCGTCTGCTCCACCTCCAGC-3
pCDNA3-HA-HA-MYC 170-439R	5'-GCTGGAGGTGGAGCAGACGGATCCGGCGTAGTCGGG-3'
pCDNA3-HA-HA-MYC 201-298F_T4	5'-CGCCGGATCCGACAGCAGCTCGCCCAAGTCC-3'
pCDNA3-HA-HA-MYC 201-298R_T4	5'-CTGCAGAATTCCTAGAGGACCAGTGGGCTGTG -3'
pCDNA3-HA-HA-MYC 208-300F	5'-GTCCTCAAGAGGTGACACGTCTCCACACATCAG-3'
pCDNA3-HA-HA-MYC 208-300R	5'-CTGATGTGTGGAGACGTGTCACCTCTTGAGGAC-3'
pCDNA3-HA-HA-MYC 208-439F_T4	5’ CGCCGGATCCTGCGCCTCGCAAGACTCCAGC 3’
pCDNA3-HA-HA-MYC 208-439R_T4	5'-CTGCAGAATTCTTACGCACAAGAGTTCCG-3
pCDNA3-HA-HA-MYC 277-357F_T4	5'- CGCCGGATCCTCAGAGTCTGGATCACCTTCTG-3'
pCDNA3-HA-HA-MYC 277-357R_T4	5'- CTGCAGAATTCTCACCTCTTGACATTCTCCTCGG -3'
pCDNA3-HA-HA-MYC 277-439F_T4	5'- CGCCGGATCCTCAGAGTCTGGATCACCTTCTG-3'
pCDNA3-HA-HA-MYC 277-439R_T4	5'-CTGCAGAATTCTTACGCACAAGAGTTCCG-3
pCDNA3-HA-HA-MYC S62A_F	5'-CTGCCCACCCCGCCCCTGGCCCCTAGCCGCCGC-3'

pCDNA3-HA-HA-MYC S62A_R	5'-GCGGCGGCTAGGGGCCAGGGGCGGGTGGGCAG-3'
pCDNA3-HA-HA-MYC S62D_F	5'-CTGCCCACCCCGCCCCTGGACCCTAGCCGCCGC-3
pCDNA3-HA-HA-MYC S62D_R	5'-GCGGCGGCTAGGGTCCAGGGGCGGGGTGGGCAG-3'
pCDNA3-HA-HA-MYC T58A_F	5'-GAAATTCGAGCTGCTGCCCGCCCCGCCCCTGTC-3'
pCDNA3-HA-HA-MYC T58A_R	5'-GACAGGGGCGGGGCGGGCAGCAGCTCGAATTTC-3'
**Primers for site directed qPCR**	
GAPDH_F	5'-GTCTCCTCTGACTTCAACAGCG-3'
GAPDH_R	5'-ACCACCCTGTTGCTGTAGCCAA-3'
Myc_F	5'-CCTGGTGCTCCATGAGGAGAC-3'
Myc_R	5'-CAGACTCTGACCTTTTGCCAGG-3'
RUNX3_F	5'-GGCAATGACGAGAACTACTCCG-3
RUNX3_R	5'-GATGGTCAGGGTGAAACTCTTCC-3'

References

1 Subramanian, A. *et al.* Gene set enrichment analysis: a knowledge-based approach for interpreting genome-wide expression profiles. *Proc Natl Acad Sci U S A* **102**, 15545-15550, doi:10.1073/pnas.0506580102 (2005).

2 Lu, J. *et al.* Subcellular localization of the alpha and beta subunits of the acute myeloid leukemia-linked transcription factor PEBP2/CBF. *Mol Cell Biol* **15**, 1651-1661, doi:10.1128/MCB.15.3.1651 (1995).

3 Mizushima, S. & Nagata, S. pEF-BOS, a powerful mammalian expression vector. *Nucleic Acids Res* **18**, 5322, doi:10.1093/nar/18.17.5322 (1990).

4 Zhang, Y. W. *et al.* A novel transcript encoding an N-terminally truncated AML1/PEBP2 alphaB protein interferes with transactivation and blocks granulocytic differentiation of 32Dcl3 myeloid cells. *Mol Cell Biol* **17**, 4133-4145, doi:10.1128/MCB.17.7.4133 (1997).

5 Bae, S. C. *et al.* Cloning, mapping and expression of PEBP2 alpha C, a third gene encoding the mammalian Runt domain. *Gene* **159**, 245-248, doi:10.1016/0378-1119(95)00060-j (1995).

6 He, X., Saint-Jeannet, J. P., Woodgett, J. R., Varmus, H. E. & Dawid, I. B. Glycogen synthase kinase-3 and dorsoventral patterning in Xenopus embryos. *Nature* **374**, 617-622, doi:10.1038/374617a0 (1995).

7 Vo, B. T. *et al.* The Interaction of Myc with Miz1 Defines Medulloblastoma Subgroup Identity. *Cancer Cell* **29**, 5-16, doi:10.1016/j.ccell.2015.12.003 (2016).

8 Hanai, J. *et al.* Interaction and functional cooperation of PEBP2/CBF with Smads. Synergistic induction of the immunoglobulin germline Calpha promoter. *J Biol Chem* **274**, 31577-31582, doi:10.1074/jbc.274.44.31577 (1999).

9 Jing, H. *et al.* A SIRT2-Selective Inhibitor Promotes c-Myc Oncoprotein Degradation and Exhibits Broad Anticancer Activity. *Cancer Cell* **29**, 297-310, doi:10.1016/j.ccell.2016.02.007 (2016).

10 Lim, K. L. *et al.* Parkin mediates nonclassical, proteasomal-independent ubiquitination of synphilin-1: implications for Lewy body formation. *J Neurosci* **25**, 2002-2009, doi:10.1523/JNEUROSCI.4474-04.2005 (2005).

11 Seeling, J. M. *et al.* Regulation of beta-catenin signaling by the B56 subunit of protein phosphatase 2A. *Science* **283**, 2089-2091, doi:10.1126/science.283.5410.2089 (1999).

12 Kitagawa, M., Fung, S. Y., Onishi, N., Saya, H. & Lee, S. H. Targeting Aurora B to the equatorial cortex by MKlp2 is required for cytokinesis. *PLoS One* **8**, e64826, doi:10.1371/journal.pone.0064826 (2013).

13 Chuang, L. S. *et al.* Aurora kinase-induced phosphorylation excludes transcription factor RUNX from the chromatin to facilitate proper mitotic progression. *Proc Natl Acad Sci U S A* **113**, 6490-6495, doi:10.1073/pnas.1523157113 (2016).
