# Supplementary material for: RUNX3 inactivates oncogenic MYC through disruption of MYC/MAX complex and subsequent recruitment of GSK3β-FBXW7 cascade
Source: Commun Biol. 2023 Jul 3;6:689. doi: 10.1038/s42003-023-05037-0 (PMC10317990; doi:10.1038/s42003-023-05037-0)
Supplement: Supplementary file 2 — Description of Additional Supplementary Files [file 42003_2023_5037_MOESM2_ESM.pdf]

Description of Additional Supplementary Files

File name: Supplementary Data 1

Description: RNA sequencing for HeLa Mock vs Dox

File name: Supplementary Data 2

Description: RNA sequencing for MKN28 Mock vs Dox

File name: Supplementary Data 3

Description: Source data for graphs
