# Supplementary material for: RUNX3 inactivates oncogenic MYC through disruption of MYC/MAX complex and subsequent recruitment of GSK3β-FBXW7 cascade
Source: Commun Biol. 2023 Jul 3;6:689. doi: 10.1038/s42003-023-05037-0 (PMC10317990; doi:10.1038/s42003-023-05037-0)
Supplement: Supplementary file 6 — Reporting Summary [file 42003_2023_5037_MOESM6_ESM.pdf]

nature portfolio

Corresponding author(s): Yoshiaki Ito

Last updated by author(s): Jun 6, 2023

Reporting Summary

Nature Portfolio wishes to improve the reproducibility of the work that we publish. This form provides structure for consistency and transparency in reporting. For further information on Nature Portfolio policies, see our Editorial Policies and the Editorial Policy Checklist.

Statistics

For all statistical analyses, confirm that the following items are present in the figure legend, table legend, main text, or Methods section.

n/a	Confirmed	
☐	☒	The exact sample size (*n*) for each experimental group/condition, given as a discrete number and unit of measurement
☐	☒	A statement on whether measurements were taken from distinct samples or whether the same sample was measured repeatedly
☐	☒	The statistical test(s) used AND whether they are one- or two-sided<br>*Only common tests should be described solely by name; describe more complex techniques in the Methods section.*
☒	☐	A description of all covariates tested
☒	☐	A description of any assumptions or corrections, such as tests of normality and adjustment for multiple comparisons
☐	☒	A full description of the statistical parameters including central tendency (e.g. means) or other basic estimates (e.g. regression coefficient) AND variation (e.g. standard deviation) or associated estimates of uncertainty (e.g. confidence intervals)
☐	☒	For null hypothesis testing, the test statistic (e.g. *F*, *t*, *r*) with confidence intervals, effect sizes, degrees of freedom and *P* value noted<br>*Give P values as exact values whenever suitable.*
☒	☐	For Bayesian analysis, information on the choice of priors and Markov chain Monte Carlo settings
☒	☐	For hierarchical and complex designs, identification of the appropriate level for tests and full reporting of outcomes
☐	☒	Estimates of effect sizes (e.g. Cohen's *d*, Pearson's *r*), indicating how they were calculated

Our web collection on statistics for biologists contains articles on many of the points above.

Software and code

Policy information about availability of computer code

Data collection

RNA samples were sent to BGI Genomics for preparation of transcriptome library and sequencing by the DNBseq™ next generation platform. Clean reads were mapped to the human reference genome hg19 using HISAT2. Data analysis was performed by the online automated platform CSI NGS Portal (in house). Western blot images were visualized by ChemiDoc MP system (Bio-Rad). Confocal immunofluorescence images were visualized with Zeiss LSM880 Airy Scan confocal microscope and analyzed with Zeiss Zen (Blue) imaging software.

Data analysis

Softwares and Algorithms
Ingenuity Pathway Analysis QIAGEN
Flowjo Tree Star https://www.flowjo.com/
Graphpad Prism 9.0 Graphpad software Graphpad Prism 9.0
Gene Set Enrichment Analysis 1
https://www.gsea-msigdb.org/gsea/index.jsp
Image Lab Bio-Rad
ImageJ ImageJ: Image Processing and analysis in Java https://imagej.nih.gov/ij/
QuantStudio™ Design and Analysis Software Applied Biosystems™
Zen 3.4 (blue edition) Zeiss

For manuscripts utilizing custom algorithms or software that are central to the research but not yet described in published literature, software must be made available to editors and reviewers. We strongly encourage code deposition in a community repository (e.g. GitHub). See the Nature Portfolio guidelines for submitting code & software for further information.

Data

Policy information about availability of data

All manuscripts must include a data availability statement. This statement should provide the following information, where applicable:

- Accession codes, unique identifiers, or web links for publicly available datasets
- A description of any restrictions on data availability
- For clinical datasets or third party data, please ensure that the statement adheres to our policy

The RNAseq data is available as Excel files in Supplementary Data 1 and 2, and at NCBI Gene Expression Omnibus (GEO) under the accession number GSE233777. Further information and requests for resources and reagents should be directed to the corresponding author, Yoshiaki Ito (yoshi_ito@nus.edu.sg)

Human research participants

Policy information about studies involving human research participants and Sex and Gender in Research.

Reporting on sex and gender	NA
Population characteristics	NA
Recruitment	NA
Ethics oversight	NA

Note that full information on the approval of the study protocol must also be provided in the manuscript.

Field-specific reporting

Please select the one below that is the best fit for your research. If you are not sure, read the appropriate sections before making your selection.

☒ Life sciences ☐ Behavioural & social sciences ☐ Ecological, evolutionary & environmental sciences

For a reference copy of the document with all sections, see nature.com/documents/nr-reporting-summary-flat.pdf

Life sciences study design

All studies must disclose on these points even when the disclosure is negative.

Sample size	We ensure that our data can be reproduced in at least two different cell lines across multiple experiments.
Data exclusions	For tumor microarray analysis,cores with insufficient tissues were excluded from the final analysis (see Methods).
Replication	All experiments have been reproduced. At least 3 replicates were obtained for RNA sequencing, proliferation assays, spheroid assays and NOD SCID gamma (NSG) mice tumorigenesis assays.
Randomization	This work is mainly in vitro studies in the described cell lines. Randomization was not a relevant feature of our work.
Blinding	Blinding is not applicable because of the nature of the experiments.

Reporting for specific materials, systems and methods

We require information from authors about some types of materials, experimental systems and methods used in many studies. Here, indicate whether each material, system or method listed is relevant to your study. If you are not sure if a list item applies to your research, read the appropriate section before selecting a response.

Materials & experimental systems

n/a	Involved in the study	
☐	☒	Antibodies
☐	☒	Eukaryotic cell lines
☒	☐	Palaeontology and archaeology
☐	☒	Animals and other organisms
☒	☐	Clinical data
☒	☐	Dual use research of concern

Methods

n/a	Involved in the study	
☒	☐	ChIP-seq
☒	☐	Flow cytometry
☒	☐	MRI-based neuroimaging

Antibodies

Antibodies used

CBFβ Rabbit mAb Abcam Cat# ab133600
c-MYC (D84C12) Rabbit mAb Cell Signaling Technology Cat# 5605
c-MYC (OTI3F2) Mouse mAb Origene Cat# TA500003
c-MYC (phospho S62) Rabbit mAb Cell Signaling Technology Cat# 13748
c-MYC (phospho T58) Rabbit mAb Abcam Cat# ab185655
E2F-1 Rabbit Antibody Cell Signaling Technology Cat# 3742
FBXW7 Rabbit Antibody proteintech Cat# 55290
FLAG M2 Mouse mAb Sigma-Aldrich Cat# F1804
FLAG Rabbit antibody Sigma-Aldrich Cat# F7425
GAPDH (14C10) Rabbit mAb Cell Signaling Technology Cat# 2118
GSK-3β (D5C5Z) XP® Rabbit mAb Cell Signaling Technology Cat# 12456
HA-Tag (C29F4) Rabbit mAb Sigma-Aldrich Cat# 3724
K48-linkage Specific Polyubiquitin (D9D5) Rabbit mAb Cell Signaling Technology Cat# 8081
MAX antibody Rabbit mAb Abcam Cat# ab199489
Miz-1 (D7E8B) Rabbit mAb Cell Signaling Technology Cat# 14300
Normal Rabbit IgG Antibody Cell Signaling Technology Cat# 2729
p21 Waf1/Cip1 (12D1) Rabbit mAb Cell Signaling Technology Cat# 2947
p53 Mouse mAb (DO-1) Santa Cruz Biotechnology Cat# sc-126
Pin1 Mouse Antibody (G-8) Santa Cruz Biotechnology Cat# sc-46660
PP2A C Subunit Rabbit Antibody Cell Signaling Technology Cat# 2038
RUNX1 (D33G6) Rabbit mAb Cell Signaling Technology Cat# 4336
RUNX2 (D1H7) Rabbit mAb Cell Signaling Technology Cat# 8486
RUNX3 (D6E2) Rabbit mAb Cell Signaling Technology Cat# 9647
RUNX3 (D9K6L) Mouse mAb Cell Signaling Technology Cat# 13089
α-Tubulin Mouse mAb Sigma-Aldrich Cat# T9026
β-Tubulin (9F3) Rabbit mAb (HRP Conjugate) Cell Signaling Technology Cat# 5346
2° Antibodies
For Immunofluorescence
Goat anti-Mouse IgG (H+L) Highly Cross-Adsorbed Secondary Antibody, Alexa Fluor 546 Invitrogen Cat# A11030
Donkey anti-Mouse IgG (H+L) Highly Cross-Adsorbed Secondary Antibody, Alexa Fluor 488 Invitrogen Cat# A21202
Donkey anti-Rabbit IgG (H+L) Highly Cross-Adsorbed Secondary Antibody, Alexa Fluor 488 Invitrogen Cat# A21206
Donkey anti-Rabbit IgG (H+L) Highly Cross-Adsorbed Secondary Antibody, Alexa Fluor 555 Invitrogen Cat# A31572
CBFβ Rabbit mAb Abcam Cat# ab133600
c-MYC (D84C12) Rabbit mAb Cell Signaling Technology Cat# 5605
c-MYC (OTI3F2) Mouse mAb Origene Cat# TA500003
c-MYC (phospho S62) Rabbit mAb Cell Signaling Technology Cat# 13748
c-MYC (phospho T58) Rabbit mAb Abcam Cat# ab185655
E2F-1 Rabbit Antibody Cell Signaling Technology Cat# 3742
FBXW7 Rabbit Antibody proteintech Cat# 55290
FLAG M2 Mouse mAb Sigma-Aldrich Cat# F1804
FLAG Rabbit antibody Sigma-Aldrich Cat# F7425
GAPDH (14C10) Rabbit mAb Cell Signaling Technology Cat# 2118
GSK-3β (D5C5Z) XP® Rabbit mAb Cell Signaling Technology Cat# 12456
HA-Tag (C29F4) Rabbit mAb Sigma-Aldrich Cat# 3724
K48-linkage Specific Polyubiquitin (D9D5) Rabbit mAb Cell Signaling Technology Cat# 8081
MAX antibody Rabbit mAb Abcam Cat# ab199489
Miz-1 (D7E8B) Rabbit mAb Cell Signaling Technology Cat# 14300
Normal Rabbit IgG Antibody Cell Signaling Technology Cat# 2729
p21 Waf1/Cip1 (12D1) Rabbit mAb Cell Signaling Technology Cat# 2947
p53 Mouse mAb (DO-1) Santa Cruz Biotechnology Cat# sc-126
Pin1 Mouse Antibody (G-8) Santa Cruz Biotechnology Cat# sc-46660
PP2A C Subunit Rabbit Antibody Cell Signaling Technology Cat# 2038
RUNX1 (D33G6) Rabbit mAb Cell Signaling Technology Cat# 4336
RUNX2 (D1H7) Rabbit mAb Cell Signaling Technology Cat# 8486
RUNX3 (D6E2) Rabbit mAb Cell Signaling Technology Cat# 9647
RUNX3 (D9K6L) Mouse mAb Cell Signaling Technology Cat# 13089
α-Tubulin Mouse mAb Sigma-Aldrich Cat# T9026
β-Tubulin (9F3) Rabbit mAb (HRP Conjugate) Cell Signaling Technology Cat# 5346
2° Antibodies

	For Immunofluorescence<br>Goat anti-Mouse IgG (H+L) Highly Cross-Adsorbed Secondary Antibody, Alexa Fluor 546 Invitrogen Cat# A11030<br>Donkey anti-Mouse IgG (H+L) Highly Cross-Adsorbed Secondary Antibody, Alexa Fluor 488 Invitrogen Cat# A21202<br>Donkey anti-Rabbit IgG (H+L) Highly Cross-Adsorbed Secondary Antibody, Alexa Fluor 488 Invitrogen Cat# A21206<br>Donkey anti-Rabbit IgG (H+L) Highly Cross-Adsorbed Secondary Antibody, Alexa Fluor 555 Invitrogen Cat# A31572
Validation	All antibodies used were validated by the manufacturer. The manufacturer website lists all the validation details.

Eukaryotic cell lines

Policy information about cell lines and Sex and Gender in Research

Cell line source(s)	AGS ATCC CRL-1739; RRID:CVCL_0139<br>HEK293T ATCC CRL-3216; RRID:CVCL_0063<br>HeLa Tet-On Clontech RRID:CVCL_IY74<br>HGC27 CellBank Australia RRID:CVCL_1279<br>MKN28 JCRB Cell Bank JCRB0253; RRID:CVCL_1416<br>MKN45 JCRB Cell Bank JCRB0254; RRID:CVCL_0434<br>MKN28-Tet-On This study
Authentication	Cell Line Authentication was done by commercial company Axil Scientific Pte. Ltd. via Fragment Analysis - STR Profiling. Also known as Genetic profiling of species specified short tandem repeat (STR) markers.
Mycoplasma contamination	All cell lines were tested and found free of mycoplasma contamination by LOOKOUT MYCOPLASMA PCR DETECTION KIT Sigma-Aldrich Cat# MP0035.
Commonly misidentified lines<br>(See ICLAC register)	There is no commonly misidentified lines used in this study.

Animals and other research organisms

Policy information about studies involving animals; ARRIVE guidelines recommended for reporting animal research, and Sex and Gender in Research

Laboratory animals	NOD SCID gamma (NSG) mice also known as NSG(JAX)-NOD.Cg-Prkdcscidll2rgtm1wjlSzJlnv from InVivos (RRID:IMSR_JAX:005557)
Wild animals	This study did not involve wild animals.
Reporting on sex	The mice used were all male. Cell line subcutaneous tumorigenesis assay were not known to be influenced by sex.
Field-collected samples	This study does not involve samples collected from the field.
Ethics oversight	All mice were handled in strict accordance with good animal practice as defined by the Institution of Animal Care and Use Committee, and the experiments were approved by the Institutional Animal Care and Use Committee and the Office of Safety, Health, and Environment at the National University of Singapore (NUS).

Note that full information on the approval of the study protocol must also be provided in the manuscript.
